# Parenting interventions to support parent/child attachment and psychosocial adjustment in foster and adoptive parents and children: A systematic review

**DOI:** 10.1002/cl2.1209

**Published:** 2022-01-05

**Authors:** Nina T. Dalgaard, Trine Filges, Bjørn C. A. Viinholt, Maiken Pontoppidan

**Affiliations:** ^1^ VIVE—The Danish Centre for Social Science Research Copenhagen Denmark; ^2^ Vive Copenhagen Denmark

## Abstract

**Background:**

Adopted children and children placed in foster care are at increased risk of developing a range of mental health, behavioural, and psychosocial adjustment problems. Previous studies suggest that due to early experiences of separation and loss some children may have difficulties forming a secure attachment relationship with the adoptive/foster parents.

**Objectives:**

The objectives of the present review were: (1) to assess the efficacy of attachment‐based interventions on measures of favourable parent/child outcomes (attachment security, dyadic interaction, parent/child psychosocial adjustment, behavioural and mental health problems, and placement breakdown) within foster and adoptive families with children aged between 0 and 17 years. (2) to identify factors that appear to be associated with more effective outcomes and factors that modify intervention effectiveness (e.g., age of the child at placement and at intervention start, programme duration, programme focus).

**Search Methods:**

Relevant studies were identified through electronic searches of bibliographic databases, governmental and grey literature repositories, hand search in specific targeted journals, citation tracking, contact to international experts and Internet search engines. The database searches were carried out to October 2020.

**Selection Criteria:**

The interventions of interest were parenting interventions aimed at helping the foster/adopted children and their parents to form or sustain a secure attachment relationship. The interventions had to be at least partly informed by attachment theory.

**Data Collection and Analysis:**

The total number of potentially relevant studies constituted 17.822 hits after duplicates were removed. A total of 44 studies (27 different populations) met the inclusion criteria and were critically appraised by the review authors. Due to critical study quality, missing numeric data and re‐use of the same data, only 24 studies analysing 16 different populations could be used in the data synthesis (children, *N* = 1302; parents, *N* = 1344). Meta‐analysis using both child and parent outcomes were conducted on each metric separately. All analyses were inverse variance weighted using random effects statistical models. Random effects weighted mean effect sizes were calculated using 95% confidence intervals (CIs). When possible, we conducted moderator analysis using meta‐regression and single factor sub group moderator analysis. Sensitivity analysis were conducted across study design and domains of the risk of bias assessment.

**Main Results:**

Ten studies analysed the effect of attachment‐based interventions on the overall psychosocial adjustment of foster or adopted children as reported by their caregivers post intervention. Measures used include the Child Behaviour Checklist, The Strengths and Difficulties Questionnaire, Brief Infant–Toddler Social and Emotional Assessment (BITSEA) and Eyberg Child Behaviour Inventory. The random effects weighted standardised mean difference (SMD) favouring the intervention group was 0.37 (95% CI, 0.10–0.65) and statistically significant. Three studies analysed the effects of attachment‐based interventions on the observed attachment security of foster and adopted children as measured by independent observation. Measures include the Strange Situation Procedure, Attachment Q‐Set, and The Emotional Availability Clinical Screener. The random effects weighted SMD was 0.59 (95% CI, −0.40–1.57) and not statistically significant. Four studies analysed the effect of attachment‐based interventions on positive child behaviour post intervention as measured by independent observation of video‐taped interaction between the child and caregivers. Measures include Disruptive Behaviour Diagnostic Observation Schedule (DB‐DOS) and Emotional Availability Scales). The random effects weighted SMD was 0.39 (95% CI, 0.14–0.64) and statistically significant. Ten studies analysed the effect of attachment‐based interventions on positive parenting behaviour post intervention as measured by independent observation of video‐taped interaction between the child and caregivers or coding of audio‐taped recordings of parental speech. Measures include Adapted Ainsworth Scales for sensitivity and noninterference, Measurement of Empathy in Adult–Child Interaction, The Dyadic Parent–Child Interaction Coding System, Reflective functioning scale, and Emotional Availability Scales. The random effects weighted SMD was 1.56 (95% CI, 0.81–2.31) and statistically significant. Nine studies analysed the effect of attachment‐based interventions on self‐reported post intervention parenting stress (Parenting Stress Index). The random effects weighted SMD was 0.24 (95% CI, 0.03–0.46.) and statistically significant. Three studies analysed the effect of attachment‐based interventions on parental post intervention self‐reported depressive symptoms (Beck Depression Inventory). The random effects weighted SMD was 0.59 (95% CI, −0.08–1.25.) and not statistically significant. Follow‐up analyses were carried out for the outcomes externalising behaviour, positive parenting, and parenting stress, but due to the low number of studies, results should be viewed with caution. Results of the single factor sub group moderator analysis suggest that it cannot be ruled out the effects differ depending on whether the interventions take place in the family home or in a clinical setting. However, it is unclear which location is associated with more positive effects as our findings differ between child and parent outcomes. Results of the sensitivity analysis showed no appreciable changes in the results following the removal of any of the studies in any of the analyses.

**Authors' Conclusions:**

Parenting interventions based on attachment theory increase positive parent/child interactional behaviours, decrease parenting stress, and increase the overall psychosocial adjustment of children in foster and adoptive families postintervention. Due to the low number of studies evidence regarding the effects of attachment‐based parenting interventions on attachment security and disorganised attachment in foster and adopted children was inconclusive. Theoretically, it is possible that child attachment security and/or attachment disorganisation cannot change within the relatively short period of time that parenting interventions typically last. It is possible that if postintervention improvements in parenting behaviours are sustained over time, it may lead to possible improvement in child attachment security and a decrease in child disorganised attachment. Thus, more longitudinal research is needed. Furthermore, evidence regarding the long‐term effects of attachment‐based parenting interventions on any outcomes was inconclusive due to too few studies, but findings suggest that attachment‐based interventions increase positive parenting behaviour at follow‐up points 3–6 months after the intervention. No study included in the present review provided a measure of placement stability or breakdown as an outcome, which could be used in the meta‐analysis. This further emphasises the need for future longitudinal research in prevention of placement breakdown.

## 1 PLAIN LANGUAGE SUMMARY

### 1.1 Parenting interventions support the overall psychosocial adjustment of children in foster and adoptive families

#### 1.1.1 The review in brief

Adopted children and children placed in foster care are at increased risk of developing a range of mental health, behavioural and psychosocial adjustment problems. Previous studies suggest that the quality of the attachment between the child and the adoptive/foster parents are important for the child's psychological development. This review finds that attachment‐based interventions for adoptive/foster parents increase child psychosocial adjustment and positive parent‐child behaviours.

For attachment security, parental depressive symptoms, and child internalising and externalising behaviour, the results were inconclusive.

#### 1.1.2 What is this review about?

Adopted children and children placed in foster care are at increased risk of developing a range of mental health, behavioural and psychosocial adjustment problems. By definition, foster and adopted children have experienced a separation from their biological parents. Furthermore, foster and adopted children have often experienced adverse events such as insufficient medical care, malnutrition, physical and emotional abuse and neglect before their initial placement. Theory therefore suggests that the attachment relationship between the child and the adoptive/foster parents may become a source of psychological vulnerability.

To prevent placement breakdown and adverse child outcomes, a number of interventions aim to support foster and adoptive families. This systematic review synthesises the research on a range of interventions commonly offered to foster and adoptive parents. Findings from the review are thus relevant for child protection and adoption agencies around the world.

#### 1.1.3 What is the aim of this review?

This Campbell systematic review assesses the effects of attachment‐based parenting interventions on parent/child attachment, psychosocial adjustment and placement stability within foster and adoptive families. It summarises findings from 27 different samples, including 19 randomised trials. The studies are primarily from the United States.

#### 1.1.4 What studies are included?

The included studies examined parenting interventions aimed at helping 0‐ to 17‐year‐old foster/adopted children and their parents to form or sustain a secure attachment relationship.

The intervention could be offered in any setting (i.e., clinic, hospital, or home) and in any format (i.e., family or multifamily therapy), but had to be at least partly based on attachment theory. Families had to be living in the Western world, defined as the OECD countries. Studies types in this review are: RCTs, QRCTs, or QES. The review does not include pre‐post studies without a control group.

This review includes 44 studies reporting on 27 different samples (19 randomised trials and eight nonrandomised studies). The meta‐analyses are based on 24 high‐quality studies reporting on 16 populations (14 randomised trials and two nonrandomised studies). The studies were from Belgium (1), Italy (2), The Netherlands (7), the UK (6) the United States (28) and were published between 1977 and 2020.

#### 1.1.5 What are the main findings of this review?

This review finds that overall, attachment‐based parenting interventions increase the psychosocial adjustment of children in foster and adoptive families and increase positive parent/child behaviours.

Evidence regarding the effects of attachment‐based parenting interventions on attachment security was inconclusive, as we did not find enough studies. Similarly, the evidence on the long‐term effects of attachment‐based parenting interventions on most outcomes was inconclusive due to very few studies, but findings suggest that attachment‐based interventions increase positive parenting behaviour three to six months after the intervention.

No study provided a measure of placement stability or breakdown that could be used in the meta‐analysis.

#### 1.1.6 What do the findings of this review mean?

Attachment‐based parenting interventions show promising short‐term effects within foster and adoptive families, which is relevant for therapists and agencies that provide support to foster and adoptive parents.

Future research should explore the long‐term effects of interventions on measures of parent/child attachment, psychosocial adjustment and most importantly on placement stability.

#### 1.1.7 How up‐to‐date is this review

The review authors searched for studies up to 2020.

## 2 BACKGROUND

### 2.1 The problem, condition, or issue

Every day, foster and adoptive parents around the globe care for children who have experienced serious adversity at a young age. From 2004 to 2010, between 29,000 and 45,000 children were internationally adopted each year (Selman, 2012). Globally, the number of inter country adoptions is declining, and in 2013 there were three times fewer international adoptions worldwide than in 2003 (Mignot, 2015). However, domestic adoption out‐number inter country adoption by far. Thus, the UN estimates that around 260,000 domestic adoptions take place worldwide each year.^1^ In addition, it is estimated that approximately 2.7 million children worldwide are currently living in residential care arrangements. That is, in either foster or institutional care (Petrowski et al., 2017).

Adopted children and children placed in foster care are at increased risk of developing a range of mental health, behavioural, and psychosocial adjustment problems (Bimmel et al., 2003; Brand & Brinich, 1999; Oswald et al., 2010; Pears & Fisher, 2005; Pecora et al., 2009). In a meta‐analysis based on 25,281 adoptees and 80,260 controls, adopted children showed more behaviour problems (effect sizes ranged between *d* 0.16–0.24) and were much more likely to be referred for mental health services than their nonadopted peers (*d*, 0.72). Furthermore, domestic adoptees showed more behaviour problems and were more likely to be referred for mental health services than international adoptees (Juffer & van IJzendoorn, 2005).

Foster children are also much more likely to experience behaviour and mental health problems than children living with their family of origin. Research on children placed in out‐of‐home care in the United States suggests that between 23% and 61% of children under the age of 5 are significantly delayed when screened for developmental problems (Stahmer et al., 2005). Similarly, a study based on 267 children in foster care aged 0–17 years found the rate of behavioural problems in the clinical range to be two and a half time the expected rate in a comparable community sample (Clausen et al., 1998). Finally, a recent study by Turney and Wildeman (2016) based on data from the 2011–2012 US National Survey of Children's Health compared parent‐reported mental and physical health outcomes of children placed in foster care to outcomes of children not placed in foster care. In this study, Turney and Wildeman (2016) conclude that children in foster care are in poor mental and physical health relative to children in the general population. Thus, children placed in foster care were twice as likely to have learning disabilities, developmental delays, and speech problems. Furthermore, children placed in foster care were three times as likely to have ADD/ADHD, five times as likely to have anxiety, six times as likely to have behavioural problems, and seven times as likely to have depression (Turney & Wildeman, 2016). When studying the health of foster children it should, however, be noted, that there may potentially be discrepancies between countries due to differences in child protection legislature regulating at what point children are placed in foster care. Placing a child in foster care may be considered an intervention in itself, however, a recent meta‐analysis based on longitudinal studies of foster children showed that foster care does neither negatively nor positively influence the developmental trajectories of foster children, meaning that the mental health and behaviour problems in foster children when they entered foster care were unlikely to decrease over time (Goemans et al., 2015). With regard to adopted children, longitudinal studies suggest a complex pattern of both vulnerability and catch‐up, in which adopted children come to resemble their none‐adopted peers. In a consensus statement, researchers from different disciplines suggest that while there are significant benefits of adoption compared to remaining in vulnerable families or institutional care, adopted children are more vulnerable than none‐adopted children and some problems are likely to to persist post adoption (Palacios, Adroher et al., 2019). This evidence points to the need for interventions to support children in both foster and adoptive families.

Mental health issues and behaviour problems in foster and adopted children are often further exacerbated in the detrimental event of a placement breakdown (Goemans et al., 2015). Within research on adoption and foster care placement, different terminology has been employed to describe placement disruptions and breakdown. In this review we use the term *placement breakdown* broadly to refer to the situation in which a foster or adopted child is either temporarily or permanently physically separated from the foster or adoptive parents regardless of the legal status. That is, whether the adoption process was finalised or not and whether the legal parental rights are terminated or not (Palacios, Rolock, et al., 2019). Placement breakdown does, however, not refer to the situation in which a foster child is returned to the biological parents due to improvements in the parenting ability or the life circumstances of the biological parents. Nonetheless, an incidence of placement breakdown is difficult to estimate precisely due to variation in terminology, research designs, measurement, and available statistics. In a recent review of the existing literature on adoption breakdown and disruption, Palacios, Adroher, et al. (2019) report incidence rates ranging from just 1%–23% in different studies. The study with the lowest incidence only measured disruptions or breakdown occurring in the timeframe when the adoption paper work was still being processed, which is not when most disruptions or breakdowns happen. In comparison, the study with the highest incidence was based on a population of children adopted at age 5–11 years. Age at adoption is known to be associated with placement breakdown, with older children being more at risk. These findings illustrate why the exact extend of the problem with adoption placement breakdown cannot be determined globally at this point. Similarly, it is estimated that between 20% and 50% of children in long term foster care will experience that their planned stay in their foster family ends prematurely (Oosterman et al., 2007). Regardless of the exact extend of the problem with placement breakdown, it is clear that it constitutes a serious risk, as placement breakdowns are both costly to the society and can have devastating consequences for vulnerable children (Newton et al., 2000; Palacios, Adroher, et al., 2019; Strijker et al., 2008).

To understand the aetiology of the problems experienced by foster and adoptive families and children, it has been suggested that the attachment relationship between the child and the adoptive/foster parents may become a source of psychological vulnerability. Bowlby's (1969) theory of attachment states that parent/child care giving is a goal‐directed behavioural system accompanied by strong motivational effects and shaped by the adaptive function of protecting the offspring. Thus, through the child's interactions with the primary caregiver and based on the caregivers' responses to the child's need of “a secure base,” inner working models of attachment are established early in life. Developing a secure attachment relationship with a parent or primary caregiver has long‐term benefits for children because of the impact on children's later adaptation and socioemotional development and thus a secure attachment style may be seen as a source of psychological resiliency (Cassidy & Shaver, 2002). Ainsworth et al. (1971, 1974) and Main and Solomon (1990) developed a typology of attachment patterns in children, which can be assessed and classified based on the child's behaviour in a laboratory exploration known as the Strange Situation Procedure. In children, attachment classification includes three categories describing organised attachment patterns; Secure, Insecure‐avoidant and Insecure‐resistant, as well as a fourth category known as disorganised‐disoriented, which may be superimposed on the existing categorisation in cases where the child exhibits behaviours characterised by breakdowns of the organised attachment pattern. Securely attached children develop basic trust in self and others, enabling them to function autonomously. In the Strange Situation Procedure securely attached toddlers are able to cope with a short separation from the caregiver and are easily comforted upon the return of the caregiver. In different ways, toddlers with insecure‐avoidant or insecure‐resistant attachment styles are less able to cope with the short separation from their primary caregiver, and they are not as easily comforted upon the return of their caregiver as the securely attached toddlers are. Some attachment scholars have proposed that toddlers in the avoidant category are frightened of appearing vulnerable. Thus, they may ignore their caregiver whilst still showing elevated signs of physiological arousal, indicating high levels of stress in response to the separation. Toddlers in the resistant category are hypothesised to perceive care giving as unpredictable and thus may show a behaviour characterised by ambivalence towards the caregiver, such as excessive crying when the caregiver leaves and an inability of be comforted upon the return (Ainsworth et al., 1978). The Disorganised‐disoriented attachment category was added to the theory based on observations of children who did not seem to fit the description of the original patterns. Main and Solomon (1990) proposed that this category was characterised by breakdowns of attachment organisation following trauma. A number of studies support the associations between disorganised‐disoriented attachment in early childhood and the subsequent development of adverse child outcome (Alpern & Repacholi, 1993; Carlson, 1998; Groh et al., 2014; Moss et al., 2006).

Adopted children and children placed in foster care share experiences of early separation from caregivers, leaving them at elevated risk for developing insecure and/or disorganised‐disoriented attachment. In a series of meta‐analyses based on 39 studies (*N* = 2912 adopted children) Van den Dries et al. (2009) found that children, who were adopted after their first birthday, showed less attachment security than their nonadopted peers did. This was similar to the attachment distributions in samples of children in foster care. Furthermore, Van de Dries et al. (2009) concluded that adopted children, regardless of age at the adoption and similarly to foster children, showed more disorganised attachment compared to their nonadopted peers (Van den Dries et al., 2009).

### 2.2 Description of the intervention

#### 2.2.1 The intervention

To prevent placement breakdown and adverse child outcomes, a number of interventions are offered to foster and adoptive families. In this review we include attachment‐based interventions aimed at helping the foster/adopted children and their parents to form or sustain a secure attachment relationship. The interventions are at least partly informed by attachment theory and aimed at enhancing parent/child attachment security and improving the psychosocial adjustment of parents and children by increasing parental sensitivity and emotional availability. Thus, included interventions all share at least one of the following treatment goals:

- Increased attachment security
- Decreased disorganised attachment
- Increased parental sensitivity
- Increased parental emotional availability
- Increased positive dyadic interaction
- Increased psychosocial adjustment of the child.

Examples of included interventions are: *Attachment and Biobehavioral Catch‐up (ABC), Video‐Feedback Intervention to Promote Positive Parenting (VIPP)*, and *Parent‐Child Interaction Therapy (PCIT)*. ABC is a manualised intervention consisting of 10 weekly 60‐min sessions delivered in the families' homes. Each session is videotaped and used for both clinical supervision of the therapists and video‐feedback for parents (Yarger et al., 2019). VIPP is a video‐feedback short‐term home‐based intervention, which exists in several different versions with and without a component that address the parental attachment representations and with an additional intervention module which seeks to promote sensitive discipline. VIPP is aimed at increasing parental sensitivity by providing parents with concrete feedback on their behaviour when interacting with the child (Juffer et al., 2008). PCIT is only partly informed by attachment theory. PCIT is a 14‐ to 20‐week manualised intervention founded on social learning, behavioural, and attachment theories. The intervention takes place in a clinical setting in which therapists coach parents from an observation room behind a two‐way mirror via a bug‐in‐the‐ear‐receiver (Allen et al., 2014).

We excluded interventions that do not involve the direct participation of at least one parent and a foster/adopted child in at least one session. Thus, we excluded individual parental or couples' therapy, parental counselling, psychoeducation, and individual child psychotherapy or adult/child support groups. Furthermore, we excluded interventions which are not based on attachment theory, such as cognitive behaviour therapy or trauma‐focused therapies.

Finally, we excluded interventions deemed unvalidated or theoretically questionable such as interventions claiming to promote “reattachment” through coercive holding, physical restraints, or re‐birthing (Chaffin et al., 2006). Unvalidated treatments refer to treatments with serious harmful side effects, treatments with no empirical evidence to support their claim of effectiveness, treatments based on ideas which fails to mesh with current accepted theory, treatments which are not discussed in professional publications such as peer reviewed journals, and treatments based exclusively on clinical observations rather than science. Please see Mercer et al. (2003) for a full description of criteria to determine if a treatment is unvalidated (Mercer et al., 2003; Zeanah et al., 2011).

### 2.3 How the intervention might work

Although the capacity for developmental change diminishes with age, change continues throughout the life cycle so that changes for better or for worse are always possible. It is this continuing potential for change that means that at no time of life is a person invulnerable to every possible adversity and also at no time of life is a person impermeable to favourable influence. It is this persisting potential for change that gives opportunity for effective therapy (Bowlby, 1988, p. 154).

By definition, foster and adopted children have experienced a separation from their biological parents. Furthermore, foster and adopted children have often experienced adverse events such as insufficient medical care, malnutrition, physical and emotional abuse, and neglect before their initial placement (Sullivan & van Zyl, 2008). Thus, the children may be physically and psychologically vulnerable at the time of the placement and this vulnerability may continue to manifest itself throughout their life course in various ways (Palacios, Rolock, et al., 2019). However, as proposed by Bowlby within the above quotation, attachment is a dynamic phenomenon, and there is continuous potential for positive changes through the child's interactions with sensitive caregivers. This potential is what attachment interventions are aimed at supporting. According to Bowlby (1988), a child will experience grief, anger and distress as a result of temporary or permanent loss of access to existing attachment figures, and this can only be resolved if the child is able to develop new attachment relationships with alternative caregivers. By supporting a child's current caregivers (e.g., the foster or adoptive parents) in meeting the needs of the child in a consistent and sensitive manner, attachment‐based interventions are proposed to be able to change the child's internal working models of attachment, leading to an increased sense of “felt security.” Attachment‐based interventions are thus designed to help foster and adoptive parents to notice and understanding subtle and overt emotional cues in their child's behaviour and to respond to these cues in a sensitive, contingent, and consistent manner. Sometimes this process may involve a reflection on the attachment history and the current “states of mind” with respect to attachment of the adoptive or foster parents themselves (Juffer et al., 2008). The reason for the assumed benefits of working with the caregiver's own state of mind with respect to attachment is that this has been identified as the strongest predictor of whether foster children will become securely attached to a foster parent or not (Dozier et al., 2001). However, in a study comparing VIPP and VIPP‐R with a control condition in a sample consisting of mothers with <14 years of education, who were classified as insecure and their first born infants, Veldenman et al. (2006) found overall differences between the two intervention groups and the control group on measures of maternal sensitivity postintervention, but no significant differences were found between the two interventions, suggesting no added benefits of working with maternal attachment representations compared to the VIPP intervention alone.

Typically, attachment‐based interventions consist of sessions in which the therapist is working with the parent(s) and children simultaneously. In subsequent sessions the parents are provided with feedback, education about attachment, and are invited to reflect upon their experiences with the child.

### 2.4 Why it is important to do this review

Based on findings on the associations between early disorganised‐disoriented attachment and subsequent adverse child outcomes, a number of interventions and programmes are aimed at supporting the development of a secure attachment relationship between parents and children (Dozier & Rutter, 2008; Dozier et al., 2001). In a meta‐analysis on the effectiveness of preventative attachment interventions on parental sensitivity and infant attachment for at risk populations 70 studies were traced, producing 88 intervention effects on sensitivity (*n* = 7636) and/or attachment (*n* = 1503). Randomised interventions appeared rather effective in changing insensitive parenting (*d* = 0.33) and infant attachment insecurity (*d *= 0.20) (Bakermans‐Kranenburg et al., 2003). In a systematic review co‐registered at Cohrane and Campbell, Barlow et al. (2015) explored the efficacy of attachment‐based parent‐infant psychotherapy on parental and infant mental health. This review focused on infants aged 0–24 months within vulnerable families (defined as families in which parents were suffering from mental health issues, drug/alcohol abuse, or were victims of domestic violence). Findings from this review suggest that parent‐infant psychotherapy is a promising approach in terms of improving infant attachment security in high‐risk families. However, there were no significant differences compared with no treatment or treatment‐as‐usual for other parent‐based or relationship‐based outcomes, and no evidence that parent‐infant psychotherapy is more effective than other ways of working with parents and infants. The review by Barlow and colleagues provides important insight into the efficacy of attachment‐based interventions, however, the findings may not be applicable to the population of the present review for two main reasons. First, the population of adoptive and foster parents are typically resourceful individuals highly motivated for participation in interventions. Second, Barlow et al. (2015) focused only on therapeutic interventions that could be described as parent‐child psychotherapy and only included infants under the age of 2 years. Juffer et al. (2017) provides a review and meta‐analysis of the effectiveness of Video‐feedback Intervention to promote Positive Parenting and Sensitive Discipline (VIPP‐SD), which is an intervention based partially on attachment theory with various populations of at‐risk parents and vulnerable children (*k* = 12). In the review positive effects of VIPP‐SD were found on measures of sensitive parenting and socio‐emotional child outcomes.

Kerr and Cossar (2014) conducted a systematic review of studies of attachment‐based interventions with foster and adoptive parents and children aged 0–17 years. This narrative review provides preliminary insights and suggests that there are positive effects of attachment‐based interventions for this population, but it lacks the methodological rigour of a Campbell review and needs to be up‐dated. Furthermore, the review by Kerr and Cossar (2014) only provides a very limited description of the quality appraisal process and it does not include meta‐analysis. Thus, the efficacy of attachmentbased interventions on measures of both attachment security and on measures of parent/child psychosocial adjustment in foster and adoptive families is yet to be thoroughly examined, which is where the present review will contribute.

Finally, a recent meta‐analytic review (*k* = 53) examined the effects of all types of parenting interventions in foster care and adoption on eight types of outcomes (Schoemaker et al., 2019). Results show positive effects on four parenting outcomes (sensitive parenting, dysfunctional discipline, parenting knowledge and attitudes and parenting stress, and on one child outcome (behaviour problems), whereas the review did not find effects for attachment security, child diurnal cortisol levels or placement disruptions. This review provides many insights, however the present review will provide an extensive risk of bias analysis of each included study, update the searches and focus exclusively on the specific effectiveness of attachment‐based interventions.

The Hague Convention of May 29, 1993 on Protection of Children and Co‐operation in Respect of Intercountry Adoption (Hague Adoption Convention) requires all signatory states to: “promote the development of adoption counselling and post‐adoption services in their States” (article 9). In Denmark, this has resulted in the 2016 establishment of national post‐adoption services providing all adoptive parents with postadoption family counselling. The present review provides knowledge of the efficacy of a range of interventions commonly offered to adoptive parents. Furthermore, the present review is relevant for child protection agencies across the world with the authority to place children in foster care.

## 3 OBJECTIVES

1) To assess the efficacy of attachment‐based interventions on measures of favourable parent/child outcomes (attachment security, dyadic interaction, parent/child psychosocial adjustment, behavioural and mental health problems and placement breakdown) within foster and adoptive families with children aged between 0 and 17 years.
2) To identify factors that appear to be associated with more effective outcomes and factors that modify intervention effectiveness (e.g., age of the child at placement and at intervention start, programme duration, programme focus).

## 4 METHODS

### 4.1 Criteria for considering studies for this review

#### 4.1.1 Types of studies

To summarise what is known about the causal effects of attachment‐based interventions on parent/child attachment and children's psychosocial adjustment, we included all studies with a well‐defined control group. Thus, the study designs eligible for inclusion were:

1. Controlled trials
  - Randomised controlled trials (RCTs)
  - Quasi‐randomised controlled trial designs (QRCTs): Here participants are allocated by means, which are not expected to influence outcomes, for example alternate allocation, participant's birth data, case number or alphabetic order.
2. Quasi‐experimental studies (QES): This category refers to both studies, where participants are allocated by other actions controlled by the researcher, or where allocation to the intervention and control group are not controlled by the researcher (e.g. by time differences or policy rules). To be included, QESs must credibly demonstrate that outcome differences between intervention and control groups are the effect of the intervention and not the result of systematic baseline differences between groups. That is, selection bias should not be driving the results. This assessment is included as part of the risk of bias tool, which we elaborate on in the Risk of bias section.
3. Studies without a control group, if they measured attachment as categorical data pre and post intervention and compare the findings with a distribution of categories in a relevant large‐scale normative nonclinical sample. The reason for this is that the purpose of attachment‐based interventions is to promote a catch‐up among adoptees/foster children post intervention. By catch‐up we mean a situation in which the distribution of attachment categories post intervention resembles that of a normative nonclinical sample (Van den Dries et al., 2009).

Studies using single group pre‐post comparisons were not be eligible.

#### 4.1.2 Types of participants

We included foster and adoptive families (both single and two‐parent families) with at least one foster or adopted child aged between 0 and 17 years at the beginning of the intervention. Families had to be residing the the Western world defined as the OECD countries.

#### 4.1.3 Types of interventions

We included attachment‐based interventions with foster and adoptive parents in any setting (i.e., clinic, hospital, or home) and in any format (i.e., family or multi‐family therapy). Interventions were all at least partly based on attachment theory and aimed at enhancing parent/child attachment security and improving the psychosocial adjustment of parents and children by increasing parental sensitivity and emotional availability.

We excluded interventions that do not involve the direct participation of at least one parent and a foster/adopted child (such as individual or couples' therapy, parental counselling, psychoeducation).

Comparison groups consist of: no treatment, treatment as usual/other interventions/treatments offered (including normal service provision), or wait‐list control.

#### 4.1.4 Types of outcome measures

##### 4.1.4.1 Primary outcomes

Studies were included in the review if they contained any of the outcomes listed below Dalgaard 2020.

Timing of outcome assessments included immediately postintervention and follow‐up time points 3–6 months after the end of the intervention.

###### 4.1.4.1.1 Child outcomes

*Overall Psychosocial Adjustment*: Child Behaviour Checklist (CBCL), The Strengths and Difficulties Questionnaire (SDQ), Brief Infant–Toddler Social and Emotional Assessment (BITSEA), and Eyberg Child Behavior Inventory (ECBI).

*Externalising problems:* CBCL, SDQ, and The Behavioral Assessment System for Children (BASC).

*Internalizing symptoms*: CBCL, SDQ, and the Anxiety sub scale from Trauma Symptoms Checklist for Young Children (TSCYC).

*Child attachment*: the Strange Situation procedure, Attachment Q‐Set and The Emotional Attachment & Emotional Availability Clinical Screener.

*Observed positive child behavior*: DB‐DOS and Emotional Availability Scales (EAS).

In some cases studies only reported subscales from CBCL and SDQ and thus we chose to extract and analyse subscale scores for internalising and externalising problems separately in addition to the overall scores. If studies reported both subscale scores and total scores we used both.

###### 4.1.4.1.2 Parent outcomes

*Observed Positive Parenting*: Adapted Ainsworth Scales for sensitivity and noninterference, Measurement of Empathy in Adult–Child Interaction, The Dyadic Parent–Child Interaction Coding System, Reflective functioning scale and EAS

*Parenting Stress*: Parenting Stress Index (PSI)

*Parental Depressive Symptoms*: Beck Depression Inventory (BDI).

###### 4.1.4.1.3 Follow‐up

Follow‐up data which could be extracted from the studies was collected between 3 and 6 months post intervention. The following follow‐up outcomes were extracted and used the the data‐synthesis: Child Externalising Behaviour, Observed Positive Parenting and Parenting Stress.

**Other outcomes:**

A list of outcomes, which could not be used in the meta‐analysis is provided in the appendices.

*Adverse outcomes*

We planned to include any adverse effects as reported in the studies as outcomes in the review as described in the protocol Dalgaard 2020

##### 4.1.4.2 Secondary outcomes

As described in the protocol for this review, we did not distinguish between primary and secondary outcomes (Dalgaard et al., 2020).

###### 4.1.4.2.1 Types of settings

This review includes attachment‐based interventions with foster and adoptive families taking place within the families' homes, in an outpatient clinic or in community‐based facilities. We excluded interventions that do not involve face‐to‐face or online one on one interaction between participants and therapists.

### 4.2 Search methods for identification of studies

We implemented a wide range of search methods and strategies to maximise coverage of relevant references, while simultaneously attempting to reduce different types of bias related to publication and dissemination systems. The different strategies and methods will be presented below.

#### 4.2.1 Selection of bibliographical databases

We selected bibliographical databases that covers journals from different academic disciplines relating to the topic of the review. We also selected databases with a general academic scope, to ensure coverage beyond the expected academic fields. We selected the follow databases:

- SocIndex via EBSCO
- EconLit via EBSCO
- ERIC via EBSCO
- CINAHL via EBSCO
- Academic Search Premier via EBSCO
- PsycINFO via EBSCO
- Science Citation Index via Web of Science
- Social Science Citation Index Web of Science
- Sociological Abstracts via Proquest search interface.

#### 4.2.2 Electronic searches

##### 4.2.2.1 Example of a search string

Below is an example of a search‐string utilised to search PsycINFO through the EBSCO‐platform. This search string was modified in according with the search interface, syntax and subject terms for each of the above standing databases.

**Table:** 

Search	Terms	Results
S19	S6 AND S10 AND S14 AND S18	4943
S18	S15 OR S16 OR S17	1,281,863
S17	DE "Intervention" OR DE "Treatment"	130,835
S16	AB (treatmen* OR intervent* OR therap* OR program*)	1,228,946
S15	TI (treatmen* OR intervent* OR therap* OR program*)	376,984
S14	S11 OR S12 OR S13	2,493,553
S13	DE "Randomized Controlled Trials" OR DE "Experimental Design" OR DE "Random Sampling" OR DE " Randomized Effectiveness Evaluation" OR DE "Clinical Trials" OR DE "Effect Size (Statistical)" OR DE “Measurement”	95,960
S12	AB (effect* OR trial* OR experiment* OR control* OR random* OR impact* OR compar*)	2,325,560
S11	TI (effect* OR trial* OR experiment* OR control* OR random* OR impact* OR compar*)	634,316
S10	S7 OR S8 OR S9	1,306,939
S9	DE "Attachment Behavior" OR DE "Attachment Disorders" OR DE "Attachment Theory" OR DE "Stress and Trauma Related Disorders" OR DE "Disinhibited Social Engagement Disorder" OR DE "Child Abuse" OR DE "Child Neglect" OR DE "Failure to Thrive" OR DE "Parent Child Relations" OR DE "Relationship Termination" OR DE "Separation Anxiety" OR DE "Separation Anxiety Disorder" OR DE "Separation Reactions OR DE "Emotional Development" OR DE "Emotional Security" OR DE "Object Relations" OR DE "Parent Child Relations" OR DE "Psychosocial Development" OR DE "Schema Therapy"	81,640
S8	AB (sensitiv* OR emoti* OR dyadic* OR attach* OR relation*)	1,201,966
S7	TI (sensitiv* OR emoti* OR dyadic* OR attach* OR relation*)	291,762
S6	S1 OR S2 OR S3 OR S4 OR S5	55,035
S5	DE "Foster Care" OR DE "Foster Children" OR DE "Foster Parents" OR DE "Adoption (Child)" OR DE "Adopted Children" OR DE "Adoptive Parents"	11,096
S4	AB out‐of‐home	2065
S3	TI out‐of‐home	589
S2	AB (adopt* OR foster*) AND AB (parent* OR child* OR famil* OR home* OR care*)	51,502
S1	TI (adopt* OR foster*) AND TI (parent* OR child* OR famil* OR home* OR care*)	7114

*Description and rationale for search terms and facets, and sensitivity of the search string*.

The search string was designed to balance sensitivity and precision. The search string contains four aspects related to the inclusion criteria of the review. To keep the search string sufficiently sensitive, we searched each aspect in either title, abstract or subject terms. Furthermore, our search terms selected for title and abstract searches were truncated (*) to enhance sensitivity.

- Search 1–6 covers the population
- Search 7–10 covers the outcome
- Search 11–14 covers the study type/methodology
- Search 15–17 covers the intervention type
- Search 18 combines the four aspects.

A full report on the search strings and results for each database search can be found in the Supporting Information Appendix.

##### 4.2.2.2 Limitations of the search string

We did not implement any language, date or other restrictions on any of the databases.

#### 4.2.3 Searching other resources

We searched a range of web‐based resources to identify references that where either unpublished, not in English, or both.

Due to the language restrictions of the review team, we selected Danish, Swedish and Norwegian as “other languages” to search in, to identify relevant unpublished literature.

Some of the resources listed contains multiple types of unpublished literature, as well as published references. The resources we searched are listed under the category of literature that is most prevalent in the resource.

*Searches for working papers and conference proceedings in English*

- OpenGrey
- Social Science Research Network
- SocArXiv.

*Searches for dissertation and theses in English*

- ProQuest Dissertation and Theses (through ProQuest interface)
- EBSCO Open Dissertations (through EBSCO interface).

*Searches for reports in English*

- Google Scholar
- Google.

*Searches for on‐going studies in English*

- Social Care Online.

*Searches for working papers, conference proceedings, dissertations and theses on other languages*

- Danish National Research Database (open access institutional repository disseminating scientific publications)
- DiVA (open access institutional repository disseminating scientific publications)
- NORA (open access institutional repository disseminating scientific publications).

*Searches for reports on other languages*

- Google.

##### 4.2.3.1 Hand search

We implemented hand searches in key journals to identify references that were poorly indexed in the bibliographical databases, as well as covering references that was published in a journal, but not yet indexed in the bibliographical databases during the search process.

Our selection of journals to hand search was based on the frequency of the journals in our pilot‐searches for designing the search‐strings in the protocol phase. Journals with the highest frequence of references in the pilot searches were selected for hand search.

Four specific journals were hand‐searched ranging from 2017 to 2020:

- Attachment & Human Development
- Adoption & Fostering
- Adoption Quarterly
- Children and Youth Services Review.

##### 4.2.3.2 Citation tracking

To identify both published studies and grey literature we utilised citation‐tracking/snowballing strategies. Our strategy was to citation‐track related identified systematic‐reviews and meta‐analyses. The review team also checked the reference lists of included primary studies. A list of references and reviews that we citation tracked are listed in the search documentation and reporting appendix.

##### 4.2.3.3 Contact with international experts

We contacted international experts to identify unpublished and ongoing studies, and provided them with the inclusion criteria for the review along with the list of included studies, and asked for any other published, unpublished or ongoing studies relevant to the review. We also contacted corresponding authors when we found references to or mentions of ongoing studies in screened publications.

### 4.3 Data collection and analysis

#### 4.3.1 Selection of studies

Independent screening and deduplication of identified records was carried out in EPPI‐Reviewer 4 version 4.12.0.0.

First, under the supervision of review authors, two team assistants independently screened titles and abstracts to exclude studies that were clearly irrelevant. Studies considered eligible by at least one assistant or studies in which there was insufficient information in the title and abstract to judge eligibility, were retrieved in full text. The full texts were screened independently by two review team assistants under the supervision of the review authors. Any disagreement of eligibility was resolved by the review authors.

#### 4.3.2 Data extraction and management

Two review authors independently coded and extracted data from included studies. Disagreements were resolved by consulting a third review author with extensive content and methods expertise. Disagreements resolved by a third reviewer can be seen in the Risk of Bias table, which is available as a Supporting Information file. Data and information was extracted on: available characteristics of participants, intervention characteristics and control conditions, research design, sample size, risk of bias and potential confounding factors, outcomes, and results.

#### 4.3.3 Assessment of risk of bias in included studies

We assessed the risk of bias in randomised studies using Cochranes revised risk of bias tool, RoB 2 (Higgins et al., 2019).

The tool is structured into five domains, each with a set of signalling questions to be answered for a specific outcome. The five domains cover all types of bias that can affect results of randomised trials.

The five domains for individually randomised trials are:

(1) Bias arising from the randomisation process;
(2) Bias due to deviations from intended interventions (separate signalling questions for effect of assignment and adhering to intervention);
(3) Bias due to missing outcome data;
(4) Bias in measurement of the outcome;
(5) Bias in selection of the reported result.

We assessed the risk of bias in nonrandomised studies using the model ROBINS–I, developed by members of the Cochrane Bias Methods Group and the Cochrane Non‐Randomised Studies Methods Group (Sterne et al., 2016). We used the latest template for completion (currently it is the version of 19 September 2016).

The ROBINS‐I tool is based on the Cochrane RoB tool for randomised trials, which was launched in 2008 and modified in 2011 (Higgins & Green, 2011).

The ROBINS‐I tool covers seven domains (each with a set of signalling questions to be answered for a specific outcome) through which bias might be introduced into nonrandomised studies:

(1) Bias due to confounding
(2) Bias in selection of participants
(3) Bias in classification of interventions
(4) Bias due to deviations from intended interventions
(5) Bias due to missing outcome data
(6) Bias in measurement of the outcome
(7) Bias in selection of the reported result.

The first two domains address issues before the start of the interventions and the third domain addresses classification of the interventions themselves. The last four domains address issues after the start of interventions and there is substantial overlap for these four domains between bias in randomised studies and bias in nonrandomised studies trials (although signalling questions are somewhat different in several places, see Sterne et al. (2016) and Higgins et al. (2019)).

Randomised study outcomes are rated on a “Low/Some concerns/High” scale on each domain; whereas nonrandomised study outcomes are rated on a “Low/Moderate/Serious/Critical/No Information” scale on each domain. The level “Critical” means: the study (outcome) is too problematic in this domain to provide any useful evidence on the effects of intervention and it is excluded from the data synthesis. The same critical level of risk of bias (excluding the result from the data synthesis) is not directly present in the RoB 2 tool, according to the guidance to the tool (Higgins et al., 2019).

We added a critical level of risk of bias to the RoB 2 tool with the same meaning as in the ROBINS‐I tool; that is, the study (outcome) is too problematic in this domain to provide any useful evidence on the effects of intervention and it is excluded from the data synthesis. We will stop the assessment of a randomised study outcome using the RoB 2 as soon as one domain is judged as “Critical.” Likewise, we will stop the assessment of a nonrandomised study outcome as soon as one domain in the ROBINS‐I is judged as “Critical.”

“High” risk of bias in multiple domains in the RoB 2 assessment tool may lead to a decision of an overall judgement of “Critical” risk of bias for that outcome and it will be excluded from the data synthesis. “Serious” risk of bias in multiple domains in the ROBINS‐I assessment tool may lead to a decision of an overall judgement of “Critical” risk of bias for that outcome and it will be excluded from the data synthesis.

##### 4.3.3.1 Confounding

An important part of the risk of bias assessment of nonrandomised studies is consideration of how the studies deal with confounding factors. Systematic baseline differences between groups can compromise comparability between groups. Baseline differences can be observable (e.g. age and gender) and unobservable (to the researcher; e.g. motivation and “ability”). There is no single nonrandomised study design that always solves the selection problem. Different designs represent different approaches to dealing with selection problems under different assumptions, and consequently require different types of data. There can be particularly great variations in how different designs deal with selection on unobservables. The “adequate” method depends on the model generating participation, that is, assumptions about the nature of the process by which participants are selected into a programme.

A major difficulty in estimating causal effects of attachment‐based interventions on child outcomes is the potential heterogeneity in the children's developmental histories. Some children have suffered extreme abuse and neglect before being placed in foster care or adopted and information about the child's experiences before the placement/adoption may not be available to the foster/adoptive parents or to the researcher. Children who have experienced physical, emotional and/or sexual abuse may not present with symptoms straight away, as there is not a straight forward causal relationship between traumatic experiences and measurable psychopathological posttraumatic symptoms. Sometimes a child may experience trauma and appear resilient at first but begin to show symptoms many years after the traumatic experiences.

Thus, differences in the children's mental health and psychosocial adjustment may appear insignificant at baseline but could potentially be an unobservable source of bias.

As there is no universal correct way to construct counterfactuals for nonrandomised designs, we looked for evidence that identification was achieved, and that the authors of the primary studies justified their choice of method in a convincing manner by discussing the assumption(s) leading to identification (e.g., the assumption(s) that make it possible to identify the counterfactual). The judgement is reflected in the assessment of the confounder unobservables in the list of confounders considered important at the outset, which can be found in the review protocol.

In addition to unobservables, we identified the following observable confounding factors to be most relevant: Age at placement/adoption and at the intervention, children's history of trauma before placement/adoption, country of origin, and socioeconomic background of foster/adoptive parents.

##### 4.3.3.2 Importance of prespecified confounding factors

The motivation for focusing on age at placement/adoption and at the intervention, children's history of trauma before placement/adoption, country of origin, and socioeconomic background of foster/adoptive parents is given below.

Children's age at placement/adoption is known to be associated with successful placement and adoption, with older children being at a higher risk of insecure attachment, behavioural problems, and placement breakdown (Oosterman et al., 2007; Palacios, Adroher, et al., 2019; Van den Dries et al., 2009). Attachment‐based interventions are most often designed to target younger children, and thus the suitability of these interventions for older children is less well established (Juffer et al., 2008). Therefore, to be sure that an effect estimate is a result from a comparison of groups with no systematic baseline differences, it is important to control for the children's age both at placement/adoption and at the intervention. For the reasons specified above, it is important to control for children's history of abuse and neglect before adoption or placement, as traumatic experiences may influence children's later developmental trajectories in a multitude of ways. We are aware, however, that in some cases the children's history of abuse and neglect will not be available to the researchers and in this case the study may still be included if there is nothing to suggest systematic differences in child abuse histories.

Specifically for adopted children, it is important to control for the country of origin, as previous research has documented systematic differences between domestic and international adoptees on measures of mental health (Juffer & van IJzendoorn, 2005), Furthermore a study comparing children adopted from US foster care, US private agencies and internationally adopted children found significant differences in mental health service utilisation use (Tan & Marn, 2013). For international adoptees, systematic differences have been found between different countries of origin, with children from countries of origin such as Romania being more at risk for later maladaptation (Marcovitch et al., 1995), whereas children adopted from China have been found to have a significantly higher parent/child relationship quality (Tan et al., 2015). A large body of research documents the impact of parental socioeconomic background on almost all aspects of children's development (Renninger et al., 2006), which is why we consider it important to control for this. Socioeconomic background factors are, e.g. adoptive/foster parents' educational level, family income, minority background, etc. A study by Tiemans et al. (2005) based on a sample of 1484 young international adoptees (aged 10–15 years) and 695 nonadopted controls in the Netherlands found that for all psychiatric diagnoses together internationally adopted children from low and middle parental economic backgrounds did not differ from comparison subjects, however internationally adopted children in families with high parental socioeconomic status were 2.17 times more likely to meet the criteria for a disorder as nonadoptees from families with high parental socioeconomic status.

##### 4.3.3.3 Effect of primary interest and important cointerventions

We are mainly interested in the effect of participating in and completion of the intended intervention, that is, the treatment on the treated effect. The risk of bias assessments was therefore carried out in relation to this specific effect. The risk of bias assessments of both randomised trials and nonrandomised studies considered adherence and differences in additional interventions (“co‐interventions”) between intervention groups. Important co‐interventions was the regular support systems available to foster/adoptive families after placement/adoption of the child.

##### 4.3.3.4 Assessment

At least two review authors independently assessed the risk of bias for each relevant outcome from the included studies. Any disagreements was resolved by a third reviewer with content and statistical expertise. See the risk of bias table, which is available as a Supporting Information file.

#### 4.3.4 Measures of treatment effect

##### 4.3.4.1 Continuous outcomes

For continuous outcomes, effects sizes with 95% confidence intervals (CIs) were calculated, where means and standard deviations were available. If means and standard deviations were not available, we calculated standardised mean differences (SMDs) from *F* ratios, *t* values, *χ* ^2^ values, and correlation coefficients, where available, using the methods suggested by Lipsey and Wilson (2001). When enough information was not yielded, we attempted to request this information from the principal investigators. Hedges' *g* was used for estimating SMDs.

##### 4.3.4.2 Dichotomous outcomes

For dichotomous outcomes (e.g., attachment classification in Juffer, Rosenboom, et al., 1997), we calculated odds ratios with 95% CIs. To calculate common metric odds, ratios were converted to SMD effect sizes using the Cox transformation.

#### 4.3.5 Unit of analysis issues

To account for possible statistical dependencies, we examined a number of issues: whether individuals had undergone multiple interventions, whether trials were cluster randomised and whether several studies were based on the same data source. We did not include any cluster‐randomised trials or trials in which individuals underwent multiple interventions.

##### 4.3.5.1 Multiple studies using the same sample of data

In total 26 studies used the same sample of data or used only a subset of a sample used in another study. We reviewed all these studies, but in the meta‐analysis we only included one estimate of the effect for each conceptual outcome from each sample of data. This was done to avoid dependencies between the “observations” (i.e., the estimates of the effect) in the meta‐analysis.

In a few cases only sub scale scores from the SDQ and CBCL was provided and in these cases we calculated a mean ES as an estimate of the total scale.

The choice of which estimate(s) to include was based on our risk of bias assessment of the studies. When there were multiple estimates of effects regarding the same/similar outcome (such as ECBI and CBCL), we extracted both outcomes, but in the analysis of child overall psychosocial adjustment we included CBCL as this was judged to provide a comprehensive measure which more closely matched the other outcomes used. When two or more studies used a subset of a sample used in another study (or studies), we included the study using the full set of participants.

##### 4.3.5.2 Multiple time points

When the results were measured at multiple time points, each outcome at each time point was analysed in a separate meta‐analysis with other comparable studies taking measurements at a similar time point. We extracted data from: (1) postintervention, (2) 3–6 months past the end of the intervention.

#### 4.3.6 Dealing with missing data

Missing data and attrition rates in the individual studies was assessed using the risk of bias tool. Studies had to permit calculation of a numeric effect size for the outcomes to be eligible for inclusion in the meta‐analysis. When studies had missing summary data, such as missing standard deviations, we derived these where possible from *F* ratios, *t* values, *χ* ^2^ values and correlation coefficients using the methods suggested by Lipsey and Wilson (2001). If these statistics were also missing, we requested the information from the study investigators, which was successful in three cases (Mersky et al., 2016; Purvis et al., 2015; Razuri et al., 2016; Van Andel et al., 2016).

When missing summary data necessary for the calculation of effect sizes could not be derived or retrieved, the study results was reported in as much detail as possible, but could not be included in the meta‐analysis.

#### 4.3.7 Assessment of heterogeneity

As the interventions deal with diverse populations of participants (both foster and adopted children within a very large age range and adopted children from different countries of origin), and we therefore expected heterogeneity among primary study outcomes, all analyses of the overall effect were inverse variance weighted using random effects statistical models that incorporate both the sampling variance and between study variance components into the study level weights. Random effects weighted mean effect sizes were calculated using 95% CIs. Heterogeneity among primary outcome studies was assessed with *χ* ^2^ (Q) tests, and *I* ^2^, and *τ* ^2^ statistics (Higgins et al., 2003). Any interpretation of Chi‐squared tests was made cautiously on account of its low statistical power.

#### 4.3.8 Assessment of reporting biases

Reporting bias refers to both publication bias and selective reporting of outcome data and results. We used funnel plots for information about possible publication bias when there were more than four studies in the analyses, which was the case with the analysis of the outcomes: child overall psychosocial adjustment, child externalising symptoms, child internalising symptoms, observed positive parenting and parenting stress.

#### 4.3.9 Data synthesis

The present review followed standard procedures for conducting systematic reviews using meta‐analysis techniques. The overall data synthesis was conducted where effect sizes were available or could be calculated, and where studies were similar in terms of the outcome measured. All meta‐analyses were performed in Revman 5.4.

All analyses were inverse variance weighted using random effects statistical models that incorporate both the sampling variance and between study variance components into the study level weights. Random effects weighted mean effect sizes were calculated using 95% CIs. Meta‐analysis of outcomes were conducted on each metric (conceptual outcomes as outlined in section “Types of outcomes measures”) and each time point (end of intervention and follow up) separately.

Studies that were coded with a “Critical” risk of bias were not included in the data synthesis.

We provided a graphical display (forest plot) of effect sizes.

Some of the trials were reported in several studies. We reviewed all such studies reporting on the same trial, but in each of the meta‐analyses we only included one estimate of the effect from each trial. This was done to avoid dependencies between the “observations” (i.e., the estimates of the effect) in the meta‐analysis.

Several studies provided results separately for two intervention groups (slight moderations of the same intervention) in trials with only one control group. As there was not a sufficient number of studies included in any of the meta analyses to use robust variance estimation (RVE) as planned, we conducted the meta analyses using a synthetic effect size (the average) to avoid dependence between effect sizes.

In a few cases only sub scale scores from the SDQ and CBCL was provided and in these cases we calculated a mean ES as an estimate of the total scale

#### 4.3.10 Subgroup analysis and investigation of heterogeneity

We planned to investigate the following factors with the aim of explaining potential observed heterogeneity: participant characteristics (e.g., adopted vs. foster, domestic vs. international adoption, child age, socioeconomic level), the duration of the intervention and intervention components (e.g., number of sessions, single vs. multifamily therapy, treatment given in the family home vs. other locations and theoretical foundation (exclusively vs. partly based on attachment theory)). However, due to either a very limited variability between studies or too few studies reporting the moderator, this was only possible for the moderators: adopted vs. foster, child age and location (in the family home vs. other locations).

For outcomes with more than five studies included in the meta analysis (child overall psychosocial adjustment, child externalising symptoms, observed positive parenting and parenting stress), we attempted to conduct moderator analysis using meta‐regression applying the RVE technique with small sample adjustment to the residuals and the Satterthwaite degrees of freedom (Satterthwaite, 1946) for tests (Tipton, 2015) in STATA. However, results were untrustworthy as degrees of freedom were <4 (as suggested by Tanner‐Smith and Tipton (2014) and Tipton (2015)).

For the outcomes child attachment, parental depression and all follow up analyses, we did not attempt to conduct meta‐regression, as the number of studies were too limited (four at most). For the outcome child internalising symptoms there was no heterogeneity.

Single factor sub group moderator analysis was used to explore the factors: adopted versus foster children, child age (<3 years of age vs. 3 years or older), and location (in the family home vs. other locations) for outcomes where there were at least two studies in each subgroup (child overall psychosocial adjustment, child externalising symptoms, observed positive parenting and parenting stress).

The subgroup analyses were inverse variance weighted using random effects statistical models that incorporate both the sampling variance and between study variance components into the study level weights. Random effects weighted mean effect sizes for each subgroup were calculated using 95% CIs.

The assessment of any difference between subgroups was based on 95% CIs. No conclusions from single factor subgroup analyses were drawn and interpretation of relationships was cautious, as they were based on subdivision of studies and indirect comparisons.

#### 4.3.11 Sensitivity analysis

A sensitivity analyses was conducted by restricting the included studies to the RCTs and subsequently restricting the remaining RCTs to the most favourable rating from the overall risk of bias judgement. As none of the studies were rated Low risk of bias overall, the most favourable overall judgement was "some concerns."

Sensitivity analysis was also conducted across domains of the risk of bias assessment, restricting studies to the most favourable rating for each domain. Furthermore, sensitivity analysis was only conducted when there were more than two studies left in the analyses.

Finally, a sensitivity analysis was performed to investigate the sensitivity of including a cluster randomised trial (Opiola & Bratton, 2016; Opiola Kristie & Bratton Sue, 2018) using an ICC equal to one.

##### 4.3.11.1 Treatment of qualitative research

We did not include qualitative research.

#### 4.3.12 Summary of findings and assessment of the certainty of the evidence

Findings of the review were summarised and the certainty of the evidence was assessed as outlined in the protocol for the review Dalgaard 2020.

## 5 RESULTS

### 5.1 Description of studies

#### 5.1.1 Results of the search

**Figure 1:**
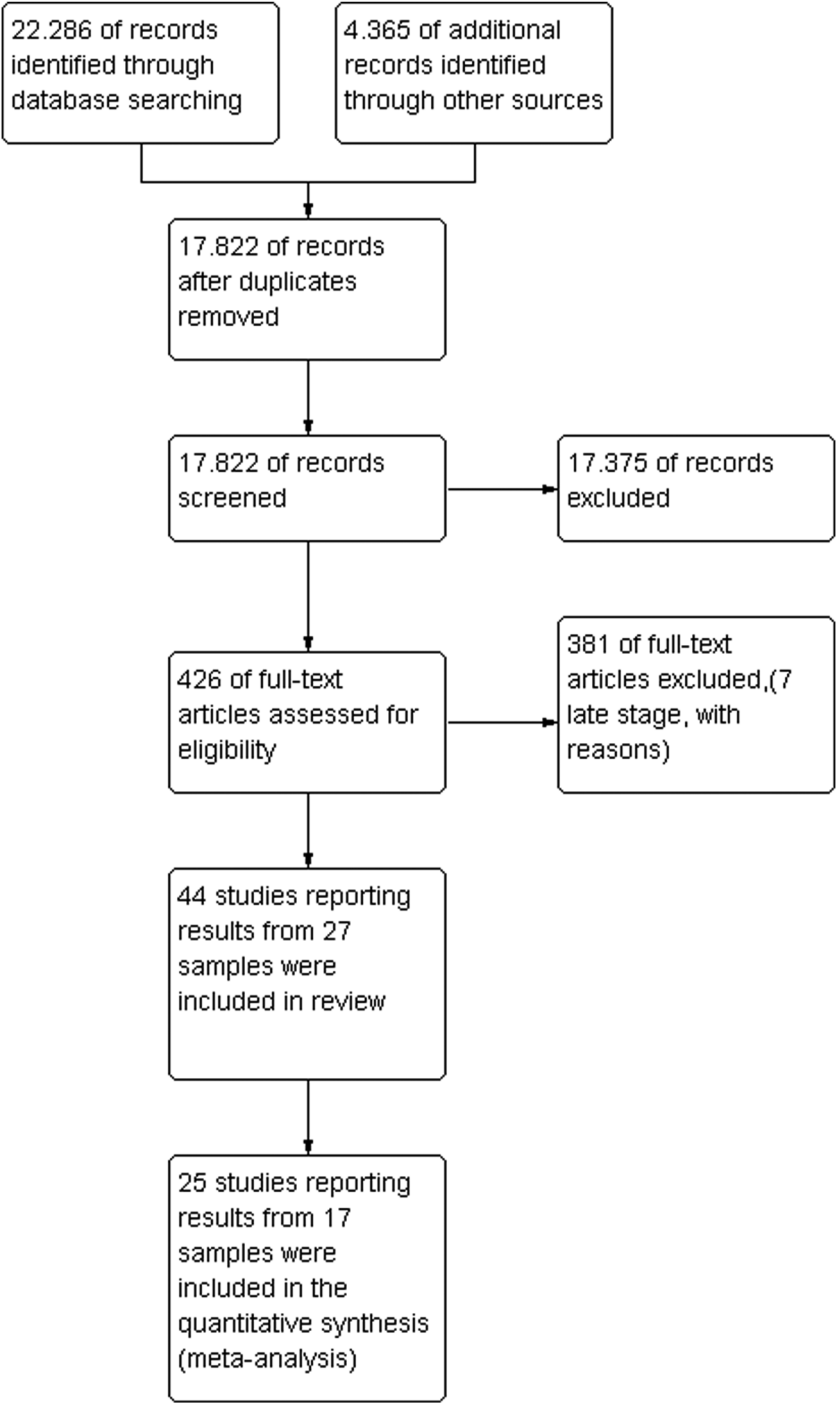
Study flow diagram

The results of the search are summarised in Figure 1. The total number of potential relevant records was 17,822 after excluding duplicates (database: 22,286 grey, hand search, snowballing and other resources: 4365). All records were screened based on title and abstract; 17,396 were excluded for not fulfilling the screening criteria, 21 records were unobtainable despite efforts to locate them through libraries and searches on the Internet and 426 records were ordered, retrieved and screened in full text. Of these, 381 did not fulfil the screening criteria and were excluded. Seven studies were excluded at a later stage, with reasons specified in the appendices under Characteristics of excluded studies. A total of 44 studies were included in the review. The references are listed in section *References to included studies*.

#### 5.1.2 Included studies

The search resulted in a final selection of 44 studies, which met the inclusion criteria for this review. In this review “studies refer to reports/papers.” Seven included studies reporting on seven samples was conducted without randomisation of participants and two studies reporting on one randomised trial was treated as nonrandomised studies as the analysis and reporting of data from this trial did not make use of the groups randomly assigned. Finally, 35 studies reported on data from 19 randomised trials.

In total the 44 studies thus analysed data from 27 different samples. Only 24 studies/publications (analysing 16 different populations) could be used in the meta‐analysis. Fiftteen studies could not be used in the data synthesis as they were judged to have too high risk of bias. Five studies did not provide enough information enabling us to calculate an effects size and standard error or did not provide results in a form enabling us to use it in the data synthesis. Finally, of the 24 studies that could be used in the data synthesis, five pairs of studies used the same two data sets and reported on the same outcome(s) and three studies reported outcomes from the same two trials.

The meta‐analysis are thus based on 24 high‐quality studies reporting on 16 samples (14 randomised trials and two nonrandomised studies).

The studies were from Belgium (1), Italy (2), The Netherlands (6), UK (7), and the United States (28) and were published between 1977 and 2020. An overview of study and trial level data by country can be found in Table 1.

**Table 1:** Summary, countries of origin

		Reduction due to		
Country	Total	Missing data	Too high risk of bias	Used in data synthesis(trials used)
Belgium	1	0	0	1 (1)
Italy	2	0	0	2 (1)
The Netherlands	7	1	1	5 (2)
UK	6	1	3	2 (2)
USA	28	3	11	14 (10)
**Total**	44	5	15	24 (16)

Among the studies, which could be used in the data synthesis, the average publication year was 2014 (range: 1997–2020), and the average number of participants in each trial or nonrandomised study was 37.73 parents (range: 7–128) and 40.46 children (range: 7–128) in the intervention groups and 33.4 parents (range: 7–128) and 36.14 children (range: 7–128) in the control groups (Tables 2 and 3).

**Table 2:** Number of participants by study

Studie	Children (control + intervention)	Parents/foster carers/adoptive parents (control + intervention)
Bywater	The intervention: 29	The intervention: 29
	The control group: 17	The control group: 17
Midgley	The intervention: 13	The intervention: 13
	Usual care: 21	Usual care: 21
Razuri/Purvis	Intervention: 128	Intervention: 128
	Control: 128	Control: 128
Mersky	129 foster/parent dyads	129 foster/parent dyads
	PCIT 14‐weeks (*N* = 35)	PCIT 14‐weeks (N = 35)
	PCIT brief 8 week (N = 48)	PCIT brief 8 week (*N* = 48)
	Waitlist control (*N* = 46)	Waitlist control (*N* = 46)
Lind	131 internationally adopted children.	124 parents
	Control: *N* = 65	
	Intervention *N* = 66	
Van Holen	Treatment group: 30	Treatment group: 30
	Control group: 33	Control group: 33
Baker	15 dyads	15 dyads
	Intervention group: 8	Intervention group: 8
	Delayed intervention group: 7	Delayed intervention group: 7
Barone (2019)	83 children	83 mothers
	Treatment group: 44	Treatment group: 44
	Control group: 39	Control group: 39
Carnes‐Holt	61 children	61 parents
	The experimental condition: 32	The experimental condition: 32
	The control condition: 29	The control condition: 29
Nilsen	18 children	18 parents
	Treatment: 11	Treatment: 11
	Control: 7	Control: 7
N'zi	14 children	14 mothers
	CDIT: 7	CDIT: 7
	Waitlist control condition: 7	Waitlist control condition: 7
Juffer 1997 (Attachment and intervention)	100 parent child dyads 60 families with only adopted children (30 treated and 30 control) and 40 families with both adopted and biological children (20 treated and 20 control)	100 parent child dyads 60 families with only adopted children (30 treated and 30 control) and 40 families with both adopted and biological children (20 treated and 20 control)
Danko	PCIT CDI only (intervention one): 7	PCIT CDI only (intervention one): 7
	PCIT + PDI: 7	PCIT + PDI: 7
	Waitlist control: 7	Waitlist control: 7
Van Andel	N = 123	N = 123
	Treatment: 65	Treatment: 65
	Control = 58	Control = 58
Schoemaker	Posttest: 27 treatment and 28 control	Posttest: 27 treatment and 28 control
Opiola	unclear	49 parents, 25: experimental group. 24: control group
Total	N = 1302	N = 1344

**Table 3:** Summary of studies used in data synthesis

Characteristic (number of studies reporting)		
Baseline year (16)	Average (SD)	2014.06 (5.81)
	Range	1997–2020
Number of participants (parents) (15)	Average (SD)	37.73 (33.05)
	Range	7–128
Number of participants, control (parents) (15)	Average (SD):	33.4 (30.90)
	Range	7–128
Number of participants (parents), total (16)	Average (SD)	74.43 (62.44)
	Range	14–256
Number of participants (children) (15)	Average (SD):	40.46 (33.61)
	Range	7–128
Number of control (children) (15)	Average (SD):	36.13 (31.80)
	Range	7–128
Number of children total (15)	Average (SD):	76.46 (64.53)
	Range	14‐256
Percent female, intervention group, children (8)	Average (SD)	52.86 (11.45)
	Range	37.5–76.5
Percent female, children, total (16)	Average (SD)	48.41 (8.035)
	Range	25.9–63.6
Mean age (years) children intervention group (8)	Average (SD)	6.24 (3.89
	Range	1.8–11.1
Mean age (years) control children (8)	Average (SD)	6.49 (3.72)
	Range	1.68–10.47
Mean age (years) children total (16)	Average (SD)	5.15 (2.86)
	Range	0.62–10.65
Mean age at placement intervention group children (years) (5)	Average (SD)	1.65 (1.69)
	Range	0.25–4.4
Mean age at placement control children (years) (3)	Average (SD)	2.15 (1.9)
	Range	0.8–3.5
Mean age at placement total children (years) (6)	Average (SD)	2.31 (1.76)
	Range	0.25–4.8
Mean number of sessions (15)	Average (SD)	10.28 (3.51)
	Range	5‐18
Mean duration of intervention in weeks (8)	Average (SD)	9.69 (2.9.8)
	Range	5–12.5

The gender distribution among the children was roughly equal with an average of 48.41% females (see Table 4).

**Table 4:** Percent, female in studies used in meta‐analysis

Study ID	Percent, female children	Mean age (years), children
Bywater (2011)	Intervention: 48.27%	Intervention: 8.86 years
	Control: 47.05%	Control: 10.47 years
Midgley (2019)	Intervention: 47%	Intervention = 11.1 years
	Control: 43%	Control: mean age = 10.2 years
Razuri (2016)/Purvis (2015)	Treatment: 50%	Treatment: mean age = 8.18 years
	Control: 50%	Control: mean age = 8.12 years
Mersky (2015)	Total sample: 56%	Total sample: 4.6 years
Lind (2020)	Intervention: 52.3% female	Intervention: 1.8 years
	Control: 50% female	Control: 1.68 years
Van Holen (2017)	Total sample: 52,4% female	Total sample: 6.14 years
Baker (2015)	Intervention group: 37.5%	Total sample: 3.5 years
	Control: 42.85%	
Barone (2019)	Total sample Female: 42%	Total sample: 3.61 years
		14–75 months
Carnes‐Holt (2014)	No information	Intervention: 5.8 years
		Control: 5.6 years
Nilsen (2007)	Intervention: 63,6%	Treatment: 8.27 years
	Control: 57,1%	Control: 7.86 years
		5–12 years
N'zi (2016)	Female: 50%	Mean age: 5.2 years
		2–7 years
Juffer (1997)	Total sample: 49%	No information
Danko (2014)	Total sample: 25.9%	Total sample: 3.56 years
Van Andel (2016)	Intervention: 51%	Intervention: 1.64 years
	Control: 49%	Control: 1.49 years
Schoemaker (2020)	Total sample: 53.3%	Total sample=3.6 years
Opiola (2018)	Total sample: 40%	Intervention:2.9 year
	Intervention group: 37.5%	Control: 4.24 year
		Total sample: 3.57 year

The mean duration of the interventions was only reported in 8 usable studies and was 9.69 week (range: 1–12.5 weeks). The average number of treatment sessions within the interventions was 10.28 (range: 5–18).

The interventions in the included studies were (number of trials):

- The Attachment and Bio‐behavioral Catch‐up (ABC) intervention (1)
- Child parent relationship therapy (CPRT) (2)
- Incredible Years (2)
- Mentalization‐Based Treatment (MBT‐foster care) (1)
- Online Emotional Attachment and Emotional Availability Intervention (1)
- Parent‐Child Interaction Therapy (PCIT) (3)
- The Foster Family Intervention (1)
- Intervention based on Social Learning Theory for foster Parents (1)
- Trust‐Based Relational Intervention (1)
- VIPP (3).

The Attachment and Bio‐behavioral Catch‐up (ABC) intervention was used in an American randomised trial with adoptive families and was reported in two publications: Yarger et al. (2020) and Lind et al. (2020).

CPRT was used in two separate randomised trials. One American trial included adoptive families and was reported in two publications: Opiola and Bratton (2016, 2018). Another American trial included adoptive families and was reported in two publications Carnes‐Holt (2010) and Carnes‐Holt and Bratton Sue (2014).

The Incredible Years intervention was used in one randomised trial in Wales including foster families (Bywater et al., 2011) and in a nonrandomised American study including foster families (Nilsen, 2007).

Intervention based on the social learning theory for foster parents was used in one randomised trial including foster children and reported in Van Holen et al. (2017). The trial took place in Belgium.

Mentalization‐Based Treatment (MBT‐foster care) was used in one randomised trial and reported in Midgley et al. (2019). The trial took place in England.

Online Emotional Attachment and Emotional Availability Intervention was used in one randomised trial and reported in: Baker et al. (2015). This randomised trial from the United States included adoptive families.

PCIT was used in three separate randomised trials. One trial took place in the United States and included foster families. It was reported in two publications: Mersky Joshua et al. (2015, 2016). The second trial also took place in the United States and included kinship foster care families. It was reported in two publications: N'zi Amanda et al. (2016) and Stevens (2011). The last American trial included foster families and was reported in one publication: Danko (2014).

The Foster Family Intervention was used in one randomised trial and reported in one publication: van Andel et al. (2016). The trial took place in Holland.

Trust‐Based Relational Intervention was used in two separate but interrelated randomised trials (used the same controls) and thus this was considered one nonrandomised study reported in Purvis et al. (2015) and Razuri et al. (2016). The randomised trial took place in the United States.

VIPP was used in three separate randomised trials that took place in Holland. One trial was originally two separate but very closely related trials, in which the same procedure and intervention was used with two samples consisting of adoptive families with and without biological children. For the purpose of this review this was considered one trial reported in five publications, which are all included in the review: Juffer, Rosenboom, et al. (1997), Juffer, Hoksbergen, et al. (1997), Stams Geert‐Jan et al. (2001), and Juffer et al. (2005, 2012). Another randomised study with adoptive families took place in Italy and was reported in two publication (Barone et al., 2019; Barone, Barone, et al., 2018). Finally VIPP was used in a randomised study in Holland using foster families, and this study was reported in Schoemaker et al. (2020).

#### 5.1.3 Excluded studies

Seven studies initially appeared eligible but were later excluded for the following reasons: one study was deemed an unvalidated treatment, one study only used pre‐test data from a trial, which is already included, one study did not report outcomes of interest in the present review, one study had an intervention which was not attachment‐based, two studies reporting on one randomised trial had an intervention which only consisted of psychoeducation and finally one study had an intervention aiming at child reunification with biological parents. A list of the late state excluded studies with reasons for exclusion can be found in Characteristics of excluded studies.

### 5.2 Risk of bias in included studies

The risk of bias coding was carried out in accordance with the protocol (Dalgaard et al., 2020) and the assessment of each of the 44 studies is available in a Supporting Information document. For a summary of the risk of bias assessments, see Tables 5 and 6.

**Table 5:** Summary of risk of bias RCT studies

Judgement	Low risk of bias	Some concerns	High risk of bias	Critical risk of bias	N.A.	Unclear	Number of studies
Risk of bias item:							
Overall judgement	0	23	3	9	0	0	35
Randomisation process	5	24	2	2	1	1	35
Deviation bias	0	31	0	2	1	1	35
Missing data	12	18	2	0	3	0	35
Measurement bias	23	9	0	0	3	0	35
Reporting bias	2	21	3	6	3	0	35

**Table 6:** Summary of the risk of bias scores for nonrandomised studies

Judgement	Low risk of bias	Moderate risk of bias	Serious risk of bias	Critical risk of bias	N.A.	Unclear	Number of studies
Risk of bias item:							
Overall judgement	0	0	3	6	0	0	9
Confounding bias	0	0	4	5	0	0	9
Selection bias	1	0	3	2	3	0	9
Classification bias	2	0	2	0	5	0	9
Deviation bias	0	3	1	0	5	0	9
Missing data	1	0	3	2	3	0	9
Measurement bias	2	2	0	0	5	0	9
Reporting bias	1	3	0	0	5	0	9

Thirty‐five studies reported the results of 19 RCTs. Of the RCT's three had a published a priori protocol or a priori analysis plan. Seven studies used nonrandomised designs and two studies reporting on the same trial used a mixture of randomisation and matching and was assessed as a nonrandomised study. None of the nonrandomised studies had an a priori protocol or an a priori analysis plan.

A total of 15 studies (six nonrandomised and nine randomised) were assessed as having an overall critical risk of bias, corresponding to a risk of bias so high that we decided not to use the studies in the data synthesis. Of these studies, five nonrandomised studies had a critical risk of confounding bias (and some of these on other risk of bias domains as well), and one nonrandomised study was rated overall Critical due to multiple ratings of Serious risk of bias on other domains. The main reason for rating them Critical risk of bias was that they failed to establish a comparison group that was balanced on important confounders and in addition, no confounders were controlled for.

Of the nine studies reporting on RCTs (five trials) and rated Critical risk of bias overall, two were rated Critical risk of bias on the Randomisation process domain (and one of them on the Deviation bias domain as well); in one study due to a high degree of self‐selection into groups despite randomised assignment of participants and in addition large pre test imbalances between groups and in the other study due to randomisation before recruitment and consent of participants, and a large part of the recruited either did not meet study criteria or declined participation and it was not possible to assess baseline imbalances.

One study was rated Critical risk of bias on the Deviation bias domain; this study was a follow up study to a trial where a wait list design was used and the study analysed a 12 months follow up outcome at a time point where those participants in the control group interested had taken up the intervention. Six studies (reporting on two trials) were rated Critical risk of bias on the Reporting bias domain. Five of these studies reported on a trial which in general was very badly reported, only the results of selected sub groups of participants as well as outcomes were reported. The last study was a very selectively reported follow up study to a trial otherwise used in the data synthesis.

As described in the protocol, we stopped the risk of bias assessment, when a study was given a judgement of “Critical risk of bias” in one domain.

No study had an overall low risk of bias. As it is not possible to blind participants or the provider of the intervention in these kinds of studies, it is not possible for a study to achieve a low risk of bias. The highest overall rating a study can acquire is Some concerns, which was aquired by 23 RCT studies.

Three nonrandomised studies had an overall rating of Serious risk of bias, and four had an overall rating of High risk of bias.

In two RCT studies, the overall risk of bias were rated differently for the reported outcomes, in these cases, the summary risk of bias scores are based on the outcomes with the most favourable rating. In N´zi Amanda et al. (2016) one outcome (Dyadic Parent‐Child Interaction Coding System) was judged to have an overall High risk of bias, whereas the remaining outcomes were judged to have an overall rating of Some concerns. In Lind et al. (2020) one outcome (child observed social‐emotional competence) was judged to have an overall High risk of bias, whereas the other outcomes were only judged to have Some concerns.

The majority of RCT studies, 24 studies, were rated Some concerns on the randomisation process (only assessed for the RCT studies), five were rated Low risk of bias, two were rated High risk of bias, two were rated Critical risk of bias and for the remaining two there were either no information (rated Unclear) or the domain was not applicable (the study had a rating of Critical risk of bias on another domain).

On the three domains only assessed for the nonrandomised studies, four were rated Serious risk of bias on the Confounding domain and five were rated Critical risk of bias; three studies were rated Serious risk of bias on the Selection bias domain, one was rated Low risk of bias, two were rated Critical risk of bias and the domain was not applicable for the remaining nonrandomised studies; two studies were rated Serious risk of bias on the Classification domain, two were rated Low risk of bias and the domain was not applicable for the remaining nonrandomised studies.

On the remaining domains (common to RCTs and nonrandomised studies) no studies were rated Low risk of bias on the Deviation domain, the majority, 34 studies, were rated Moderate risk of bias/Some concerns, one study was rated Serious risk of bias, two were rated Critical risk of bias and the remaining studies were either unclear or not applicable in this domain. On the Missing data domain the majority were rated either Low risk of bias (13 studies) or Some concerns (18 studies), five were rated High/Serious risk of bias and the remaining studies were not applicable in this domain. On the Measurement bias domain most (25 studies) were rated Low risk of bias (one of these studies in addition reported an outcome that was rated Some concerns), 11 were rated Moderate risk of bias/Some concerns (one of these studies in addition reported an outcome that was rated High risk of bias) and the remaining studies were not applicable in this domain. Finally, on the Reporting bias domain three studies were rated Low risk of bias, the majority (24 studies) were rated Moderate risk of bias/Some concerns (one of these studies in addition reported an outcome that was rated High risk of bias), three were rated High risk of bias, six were rated Critical risk of bias and the remaining studies were not applicable in this domain.

### 5.3 Effects of interventions

#### 5.3.1 Synthesis of results

The analyses presented in this section restrict attention to those studies that permitted extraction of an effect size, and were not rated Critical risk of bias (44 studies with data from 27 different samples).

All effect sizes were coded such that a larger effect size indicated better outcomes for the treated group (children or caregivers). Effect sizes that were derived from dichotomous measures were converted to a SMD using the methods outlined in section Methods. To carry out a meta‐analysis, every study must have a comparable effect size. We synthesise effects separately by type of outcome (conceptual outcomes as outlined in section “Types of outcomes measures”) and time point (end of intervention and follow up).

##### 5.3.1.1 Child outcomes at end of intervention

###### 5.3.1.1.1 Overall psychosocial adjustment

**Figure 2:**
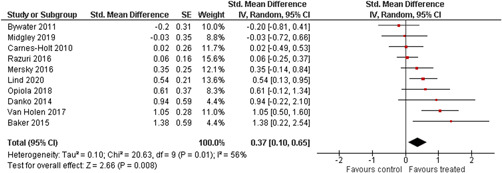
(Analysis 1.1) Child outcomes, overall psychosocial adjustment

Ten studies analysed the effect of attachment‐based interventions on the overall psychosocial adjustment of foster or adopted children as reported by their caregivers post intervention. Measures used include the Child Behavior Checklist, The Strengths and Difficulties Questionnaire, BITSEA and Eyberg Child Behavior Inventory. Two of the reported results indicate a negative effect but the weighted average is positive, favouring the treated group and statistically significant. The random effects weighted SMD is 0.37 (95% CI, 0.10–0.65). The forest plot is displayed in Figure 2. There is some heterogeneity between the studies; the estimated *τ* ^2^ is 0.10, *Q* = 20.63, df = 9 and *I* ^2^ is 56%.

###### 5.3.1.1.2 Externalising behaviour problems

**Figure 3:**
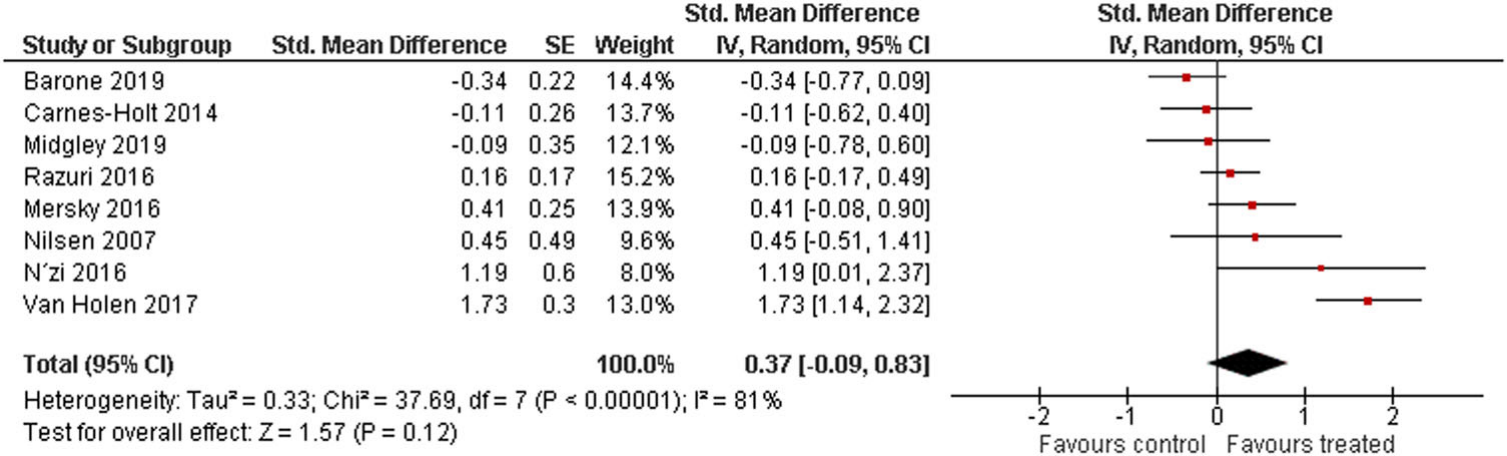
(Analysis 1.2) Child outcomes, externalising behavior

Eights studies analysed the effect of attachment‐based interventions on the externalising behaviour problems of foster and adopted children using sub scales from the Child Behavior Checklist, The Strengths and Difficulties Questionnaire and BASC as reported by their caregivers post intervention. Two of the reported results indicate a negative effect but the weighted average is positive, favouring the treated group but not statistically significant. The random effects weighted SMD is 0.37 (95% CI −0.09–0.83). The forest plot is displayed in Figure 3. There is a large degree of heterogeneity between the studies; the estimated *τ* ^2^ is 0.33, Q = 37.69, df = 7 and *I* ^2^ is 81%.

###### 5.3.1.1.3 Internalising symptoms

**Figure 4:**
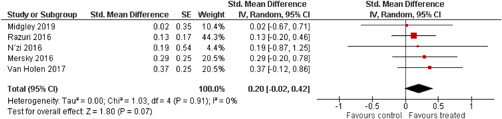
(Analysis 1.3) Child outcomes, internalising symptoms

Five studies analysed the effect of attachment‐based interventions on internalising symptoms of the foster and adopted children post intervention as reported by their caregivers. Measures include sub scales from the Child Behavior Checklist, The Strengths and Difficulties Questionnaire and the Anxiety sub scale from TSCYC. All the reported results indicate a positive effect and the weighted average is positive, favouring the treated group and statistically significant. The random effects weighted SMD is 0.20 (95% CI −0.02–0.42). The forest plot is displayed in Figure 4. There is no heterogeneity between the studies; the estimated *τ* ^2^ is 0.00, *Q* = 1.03, df = 4 and *I* ^2^ is 0%.

###### 5.3.1.1.4 Observed child attachment security

**Figure 5:**
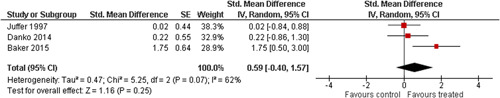
(Analysis 1.4) Child outcomes, observed attachment security

Three studies analysed the effects of attachment‐based interventions on the observed attachment security of foster and adopted children as measured by independent observation. Measures include the Strange Situation procedure, Attachment Q‐Set and The Emotional Attachment & Emotional Availability Clinical Screener. All the reported results indicate a positive effect and the weighted average is positive, favouring the treated group but not statistically significant. The random effects weighted SMD is 0.59 (95% CI, −0.40–1.57). The forest plot is displayed in Figure 5. There is some degree of heterogeneity between the studies; the estimated *τ* ^2^ is 0.47, *Q* = 5.25, df = 2 and *I* ^2^ is 62%.

###### 5.3.1.1.5 Observed positive child behaviour

**Figure 6:**
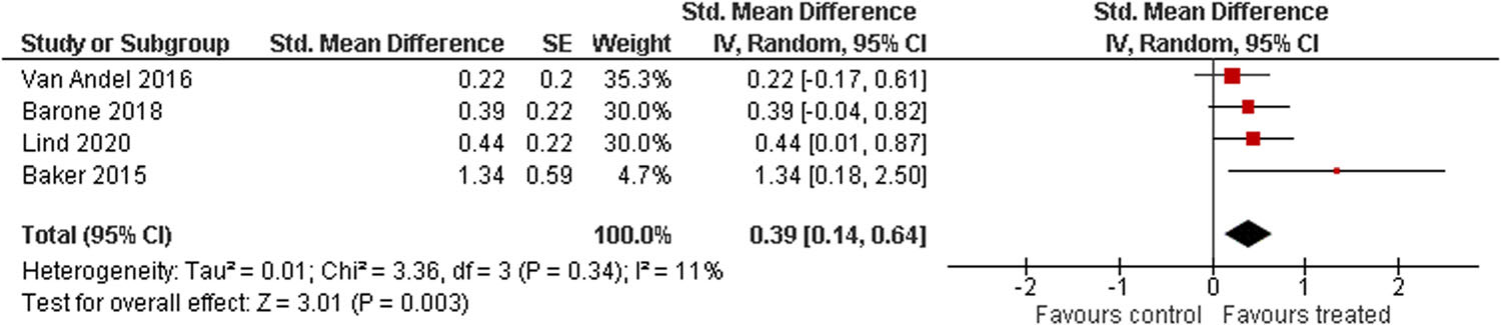
(Analysis 1.5) Child outcomes, observed positive child behaviour

Four studies analysed the effect of attachment‐based interventions on positive child behaviour post intervention as measured by independent observation of video‐taped interaction between the child and caregivers. Measures include DB‐DOS and Emotional Availability Scales. All the reported results indicate a positive effect and the weighted average is positive, favouring the treated group and statistically significant The random effects weighted SMD is 0.39 (95% CI, 0.14–0.64). The forest plot is displayed in Figure 6. There is very little heterogeneity between the studies; the estimated *τ* ^2^ is 0.01, *Q* = 3.36, df = 3 and *I* ^2^ is 11%.

#### 5.3.2 Parent outcomes at end of intervention

##### 5.3.2.1 Observed positive parenting behaviour

**Figure 7:**
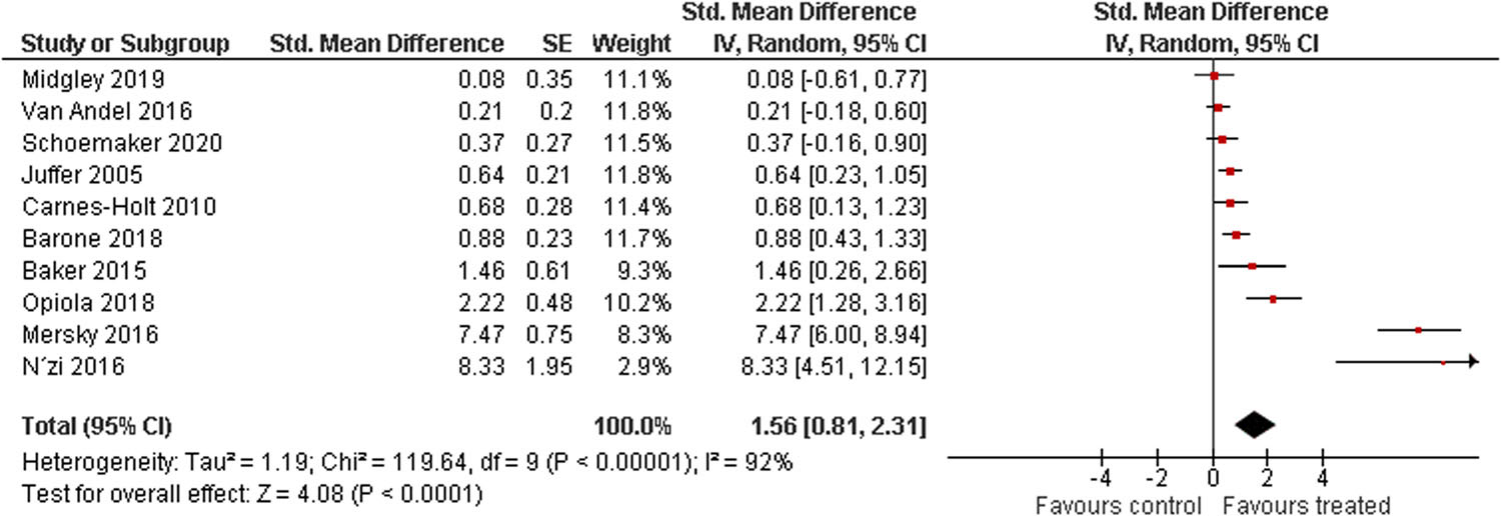
(Analysis 2.1) Parent outcomes, observed positive parent behaviour

Ten studies analysed the effect of attachment‐based interventions on positive parenting behaviour post intervention as measured by independent observation of video‐taped interaction between the child and caregivers or coding of audio‐taped recordings of parental speech. Measures include Adapted Ainsworth Scales for sensitivity and noninterference, Measurement of Empathy in Adult–Child Interaction, The Dyadic Parent–Child Interaction Coding System, Reflective functioning scale and Emotional Availability Scales. All the reported results indicate a positive effect; two of the individual study effect sizes are very large. The weighted average is large and positive, favouring the treated group and statistically significant. The random effects weighted SMD is 1.56 (95% CI, 0.81–2.31). The forest plot is displayed in Figure 7. There is a very large degree of heterogeneity between the studies; the estimated τ^2^ is 1.19, *Q* = 119.64, df = 9 and *I* ^2^ is 92%.

##### 5.3.2.2 Parenting stress

**Figure 8:**
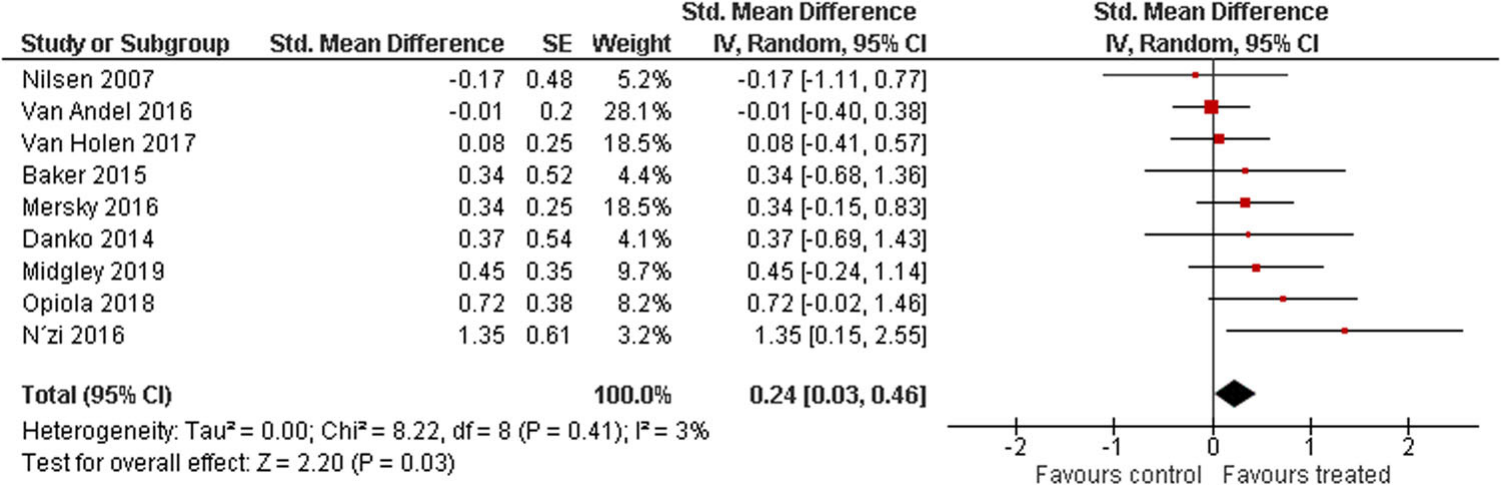
(Analysis 2.2) Parent outcomes, parenting stress

Nine studies analysed the effect of attachment‐based interventions on self‐reported post intervention parenting stress as measured by the Parenting Stress Index. Two of the reported results indicate a negative effect but the weighted average is positive, favouring the treated group and statistically significant. The random effects weighted SMD is 0.24 (95% CI, 0.03–0.46.). The forest plot is displayed in Figure 8. There is almost no heterogeneity between the studies; the estimated *τ* ^2^ is 0.00, *Q* = 8.22, df = 8 and *I* ^2^ is 3%.

Three studies reported the domain scores from the PSI in addition to the total scale score. We did not conduct meta‐analysis on each of the domain scores separately, as we deemed the construct validity of the domain scores to be unclear, and thus we chose to only use the total scale score as a comprehensive measure of parenting stress.

##### 5.3.2.3 Parental depressive symptoms

**Figure 9:**
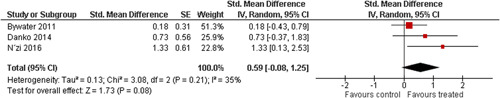
(Analysis 2.3) Parent outcomes, parental depressive symptoms

Three studies analysed the effect of attachment‐based interventions on post intervention self‐reported depressive symptoms as measured by Beck Depression Inventory. Two of the reported results indicate a negative effect but the weighted average is positive, favouring the treated group but not statistically significant. The random effects weighted SMD is 0.59 (95% CI −0.08–1.25.). The forest plot is displayed in Figure 9. There is a small degree of heterogeneity between the studies; the estimated *τ* ^2^ is 0.13, *Q* = 3.08, df = 2 and *I* ^2^ is 35%.

##### 5.3.2.4 Other outcomes

In addition a number of outcomes were reported in a single study only. The outcomes were from a variety of measures: Adult–Adolescent Parenting Inventory, Arnold Parenting Scale, Attachment Q‐Sort, Brief parental self‐efficacy scale, Child–Parent Relationship Scale, Emotional Availability Self‐Report, Erickson scale for supportive presence (based on video tapes), Eyberg Child Behavior Inventory, Indiscriminate Friendliness Interview, Questionnaire Attitudes towards Parenting, Revised child anxiety and depression scale (foster parent report), Revised child anxiety and depression scale (self report), Strengths and Difficulties Questionnaire (self‐report), The Behavioral Assessment System for Children and Trauma Symptoms Checklist for Young Children. The effect sizes and 95% CIs are reported in Table 14.

#### 5.3.3 Outcomes at follow‐up

##### 5.3.3.1 Child externalising behaviour problems

**Figure 10:**
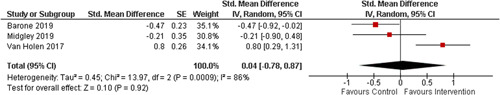
(Analysis 3.1) Follow up, child externalising behaviour

Three studies analysed the effect of attachment‐based interventions on the externalising behaviour problems of foster and adopted children using sub scales from the Child Behavior Checklist and The Strengths and Difficulties Questionnaire as reported by their caregivers 3–6 months after the intervention was finished. Two of the reported results indicate a negative effect but the weighted average is positive, favouring the treated group but not statistically significant. The random effects weighted SMD is 0.04 (95% CI, −0.78–0.87). The forest plot is displayed in Figure 10. There is a large degree of heterogeneity between the studies; the estimated *τ* ^2^ is 0.45, *Q* = 13.97, df = 2 and *I* ^2^ is 86%.

##### 5.3.3.2 Child internalising behaviour problems

**Figure 11:**
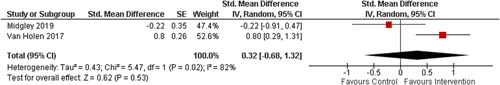
(Analysis 3.2) Forest plot of comparison: 3 follow up, child internalising behavior

Two studies analysed the effect of attachment‐based interventions on the internalising behaviour problems of foster and adopted children using sub scales from the Child Behavior Checklist and The Strengths and Difficulties Questionnaire as reported by their caregivers 3–6 months after the intervention was finished. One of the reported results indicate a negative effect but the weighted average is positive, favouring the treated group but not statistically significant. The random effects weighted SMD is 0.32 (95% CI, −0.68–1.32). The forest plot is displayed in Figure 11. There is a large degree of heterogeneity between the studies; the estimated *τ* ^2^ is 0.43, *Q* = 5.47, df = 1 and *I* ^2^ is 82%.

##### 5.3.3.3 Observed positive parenting

**Figure 12:**
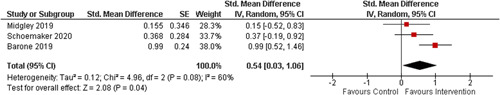
(Analysis 3.3) Follow up, observed positive parenting

Three studies analysed the effect of attachment‐based interventions on positive parenting behaviour as measured by independent observation of video‐taped interaction between the child and caregivers or in taped recordings of parental speech samples 3–6 months past the end of the intervention. Measures include Adapted Ainsworth Scales for sensitivity and noninterference, Reflective functioning scale and Emotional Availability Scales. All the reported results indicate a positive effect and the weighted average is positive, favouring the treated group and statistically significant. The random effects weighted SMD favouring the intervention group is 0.54 (95% CI, 0.03–1.06). The forest plot is displayed in Figure 12. There is some degree of heterogeneity between the studies; the estimated *τ* ^2^ is 0.12, *Q* = 4.96, df = 2 and *I* ^2^ is 60%.

##### 5.3.3.4 Parenting stress

**Figure 13:**
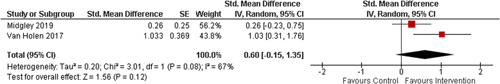
(Analysis 3.4) Follow up, parenting stress

Two studies analysed the effect of attachment‐based interventions on self‐reported post intervention parenting stress as measured by the Parenting Stress Index. Both the reported results indicate a positive effect and the weighted average is positive, favouring the treated group but not statistically significant. The random effects weighted SMD is 0.60 (95% CI, −0.15–1.35). The forest plot is displayed in Figure 13. There is some degree of heterogeneity between the studies; the estimated *τ* ^2^ is 0.20, *Q* = 3.01, df = 1 and *I* ^2^ is 67%.

#### 5.3.4 Other outcomes

In addition a number of follow up outcomes were reported in a single study only. The outcomes were from a variety of measures: Brief parental self‐efficacy scale, Emotional Availability scales, Revised child anxiety and depression scale (foster parent report), Revised child anxiety and depression scale (self report) and the Strengths and Difficulties Questionnaire (Foster care report). The effect sizes and 95% CIs are reported in Table 15.

### 5.4 Subgroup analysis and investigation of heterogeneity

The included studies differed in terms of their participants characteristics, duration of the intervention and intervention components. With between two and ten studies in a single meta‐analysis, the statistical power to detect heterogeneity of effects was quite low; nevertheless, evidence of statistical heterogeneity was found; in some analyses it was substantial. Due to missing data and invariability between studies on most of the moderators we planned to investigate, it was only possible to investigate the impact of children being adopted versus foster children, child age (<3 years of age vs. 3 years or older), and location (in the family home vs. other locations). We only performed subgroup analysis if there were at least two studies in each subgroup (child overall psychosocial adjustment, child externalising symptoms, observed positive parenting and parenting stress). All the results rely strictly on variation *between* studies, not within. Making inferences about different effect sizes among subgroups on the basis of between‐study differences entails a higher risk compared to inferences made on the basis of within study differences (Oxman & Guyatt, 1992). One should therefore be careful when interpreting estimates that rely on variation between studies. We have drawn no overall conclusion because the analysis is based on a subset of the meta‐analyses. The assessment of any difference between the subgroups is based on 95% CIs and interpretation of relationships is cautious.

#### 5.4.1 Child outcomes

##### 5.4.1.1 Overall psychosocial adjustment

###### 5.4.1.1.1 Foster/adopted

**Figure 14:**
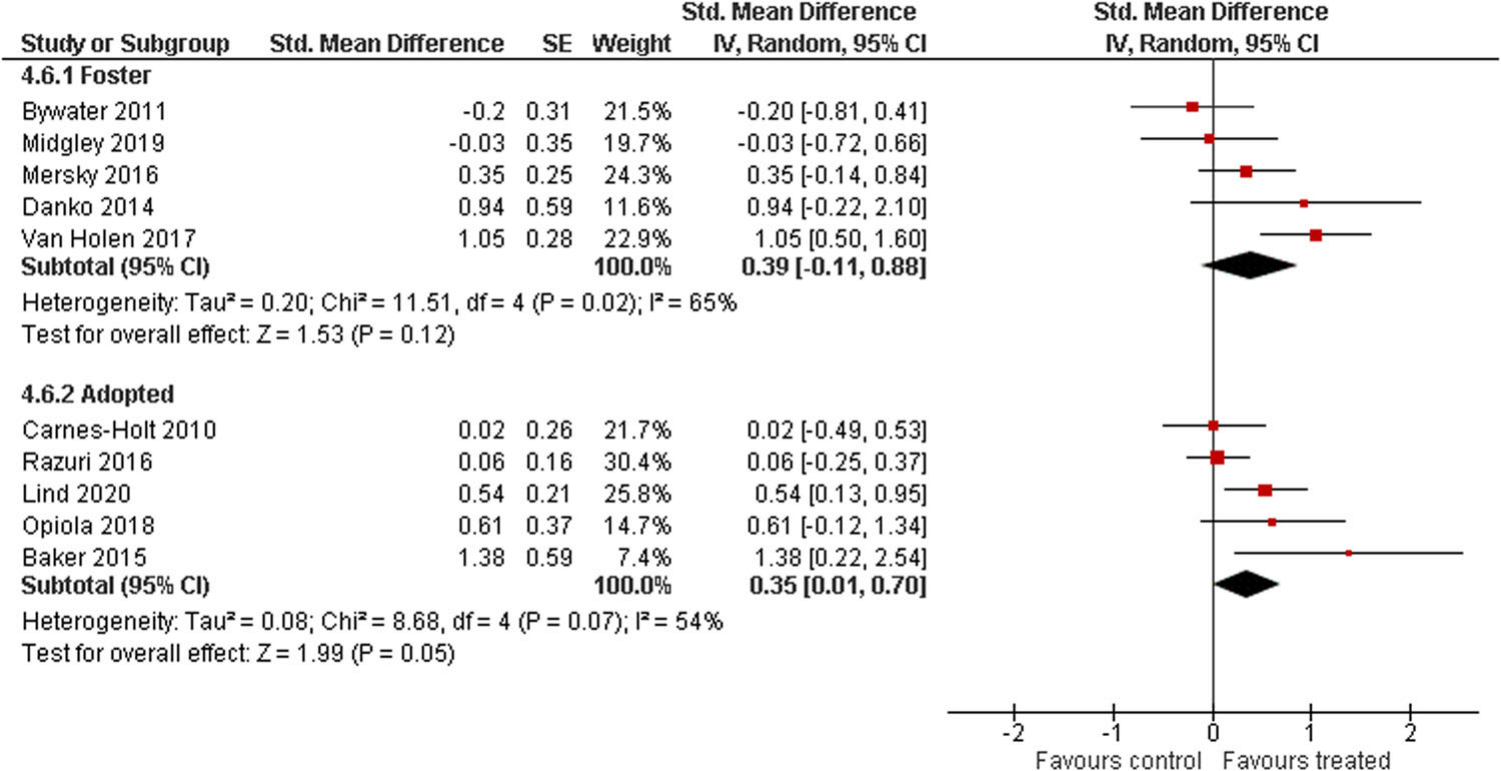
(Analysis 4.6) Forest plot of comparison: 4 subgroup child outcomes, outcome: 4.6 adopted subgroup child overall psychosocial adjustment

Of the ten studies providing effect estimates of the overall child psychosocial adjustment, five used a sample of foster children only and five used a sample of adopted children only. The forest plot for the ten effect estimates is displayed in Figure 14. Pooled results for the two subgroups showed a statistically nonsignificant positive effect of 0.39 (95% CI, −0.11–0.88) for foster children and a statistically significant positive effect of 0.35 (95% CI, 0.10–0.70) for adopted children. There was some degree of heterogeneity of effects among studies in the subgroup of foster children (*τ* ^2^ = 0.20, *I* ^2^ = 65%) and for adopted children (*τ* ^2^ = 0.08, *I* ^2^ = 54%). The CIs of the subgroups overlapped. There was no evidence to support the hypothesis that the effect differs by foster/adopted status.

###### 5.4.1.1.2 Age

**Figure 15:**
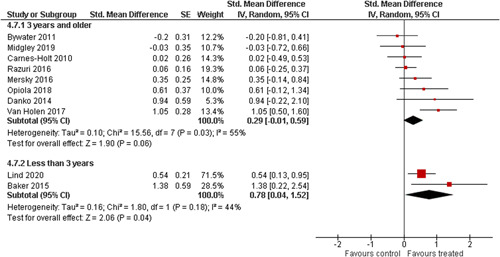
(Analysis 4.7) Forest plot of comparison: 4 subgroup child outcomes, outcome: 4.7 age subgroup child overall psychosocial adjustment

Eight studies used a sample of children on average 3 years or older and two studies used a sample of children on average <3 years. The forest plot for the ten effect estimates is displayed in Figure 15. Pooled results for the two subgroups showed a statistically nonsignificant positive effect of 0.29 (95% CI, −0.01–0.59) for children on average aged 3 years or older and a statistically significant positive effect of 0.78 (95% CI, 0.04–1.52) for children on average <3 years old. There was some degree of heterogeneity of effects among studies in the subgroup of children on average aged 3 years or older (*τ* ^2^ = 0.10, *I* ^2^ = 55%) and for children on average <3 years old (*τ* ^2^ = 0.16, *I* ^2^ = 44%). The CIs of the subgroups overlapped. There was no evidence to support the hypothesis that the effect differs by children's age.

###### 5.4.1.1.3 Location

**Figure 16:**
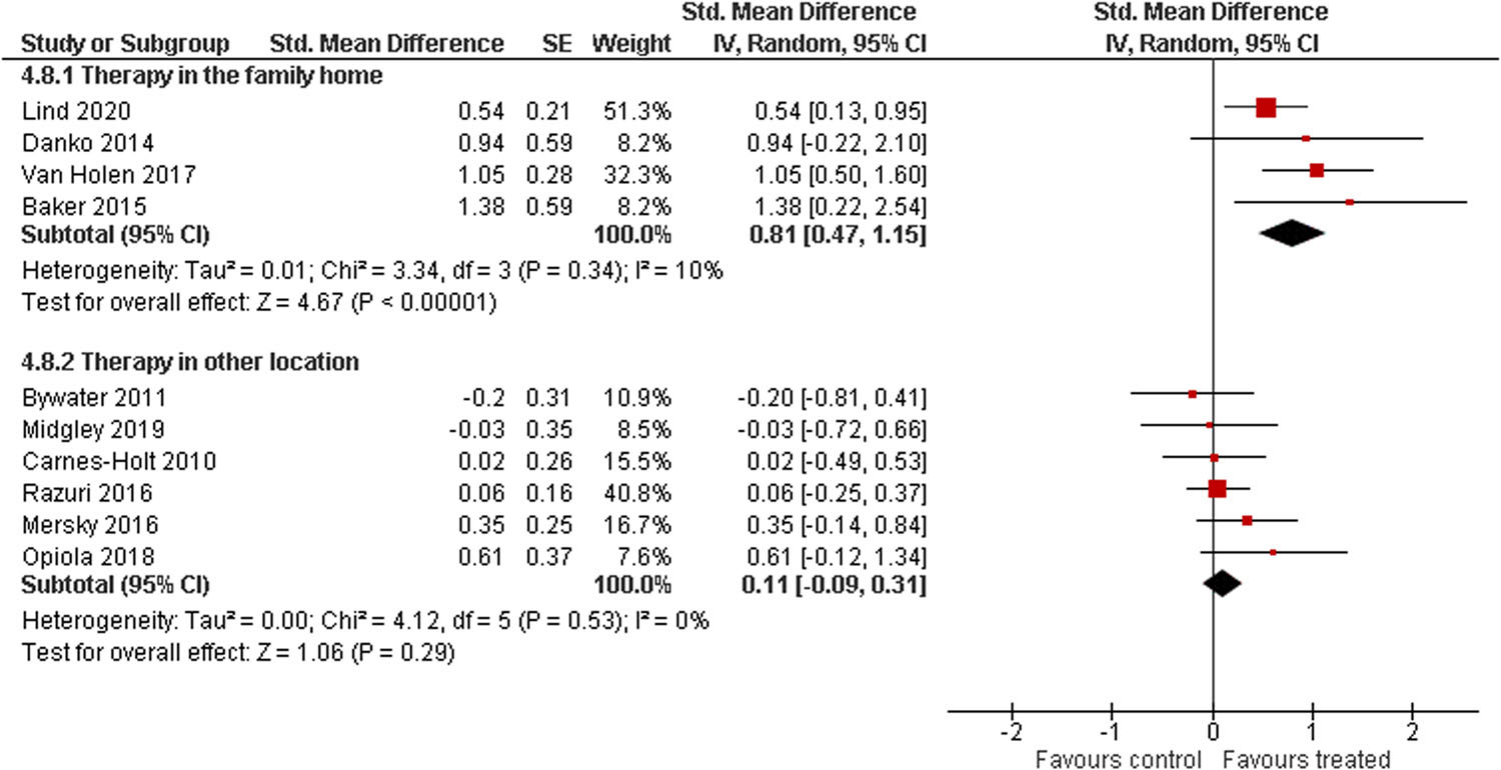
(Analysis 4.8) Forest plot of comparison: 4 subgroup child outcomes, outcome: 4.8 location subgroup child overall psychosocial adjustment

Four studies analysed interventions in the family home and six analysed interventions in another location. The forest plot for the ten effect estimates is displayed in Figure 16. Pooled results for the two subgroups showed a statistically significant positive effect of 0.81 (95% CI, 0.47–1.15) for interventions in the family home and a statistically nonsignificant positive effect of 0.11 (95% CI, −0.09–0.31) for interventions in another location. There was a small degree of heterogeneity of effects among studies in the subgroup of therapy in the family home (*τ* ^2^ = 0.01, *I* ^2^ = 10%) and no heterogeneity for therapy in another location (*τ* ^2^ = 0.00, *I* ^2^ = 0%). The CIs of the subgroups did not overlap, hence we can not rule out that the effects differ by location.

##### 5.4.1.2 Externalising behaviour problems

###### 5.4.1.2.1 Foster/adopted

**Figure 17:**
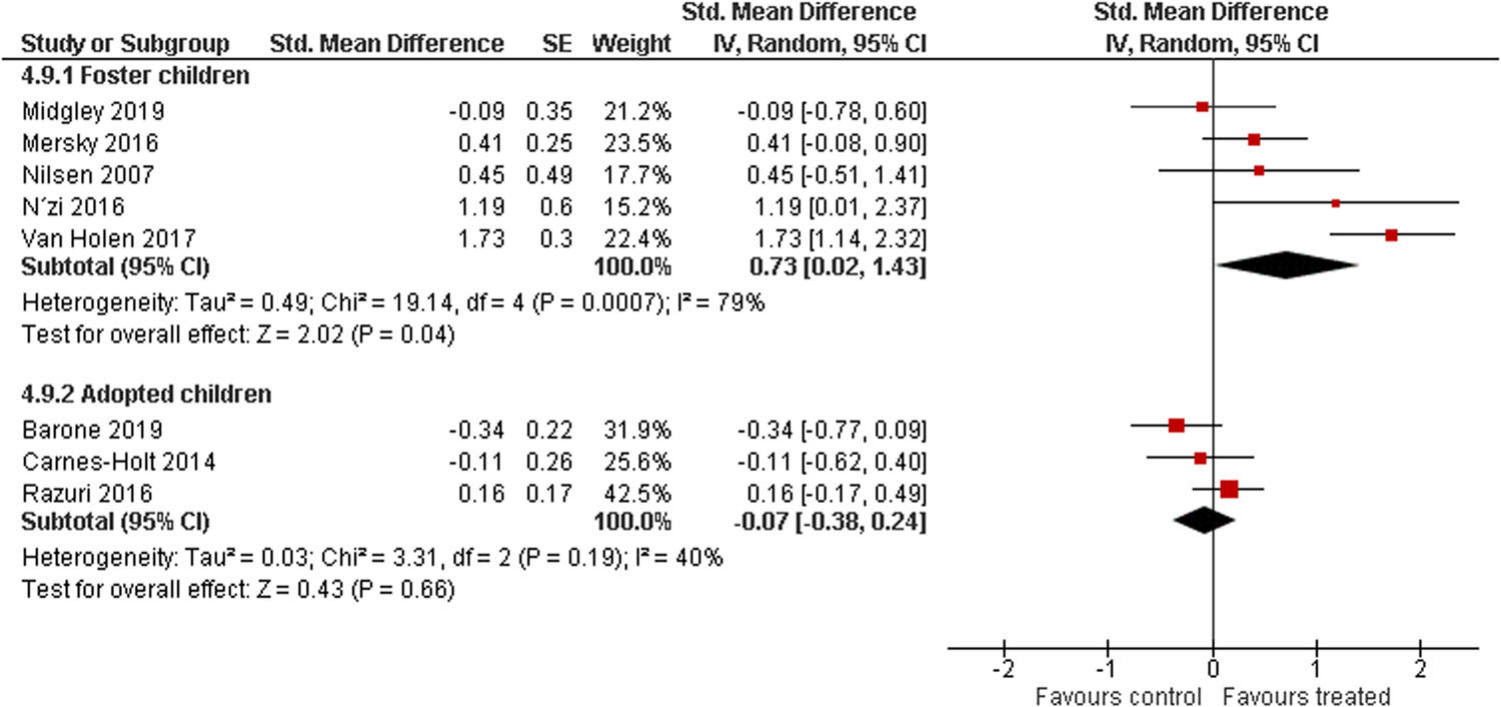
(Analysis 4.9) Forest plot of comparison: 4 subgroup child outcomes, outcome: 4.9 adopted subgroup externalising behaviour

Of the eight studies providing effect estimates of children's externalising behaviour problems, five used a sample of foster children only and three used a sample of adopted children only. The forest plot for the eight effect estimates is displayed in Figure 17. Pooled results for the two subgroups showed a statistically significant positive effect of 0.73 (95% CI, 0.02–1.43) for foster children and a statistically nonsignificant negative effect of −0.03 (95% CI, −0.38–0.24) for adopted children. There was a somewhat large degree of heterogeneity of effects among studies in the subgroup of foster children (*τ* ^2^ = 0.49, *I* ^2^ = 79%) and a smaller degree of heterogeneity for adopted children (*τ* ^2^ = 0.03, *I* ^2^ = 40%). The CIs of the subgroups overlapped. There was no evidence to support the hypothesis that the effect differs by foster/adopted status.

###### 5.4.1.2.2 Age

All of the studies used a sample of children on average 3 years or older.

###### 5.4.1.2.3 Location

**Figure 18:**
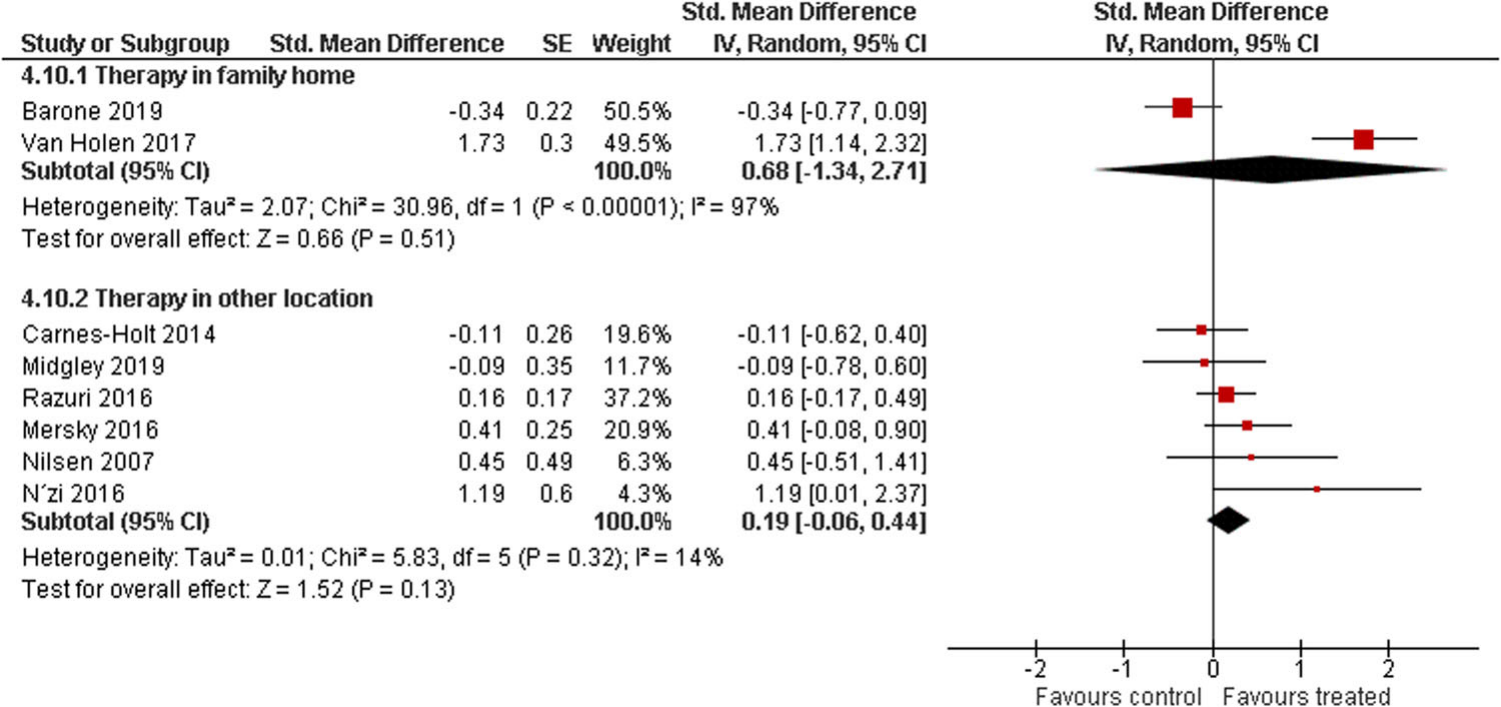
(Analysis 4.10) Forest plot of comparison: 4 subgroup child outcomes, outcome: 4.10 location subgroup externalising behaviour

Two studies analysed interventions in the family home and six analysed interventions in another location. The forest plot for the eight effect estimates is displayed in Figure 18. Pooled results for the two subgroups showed a statistically nonsignificant positive effect of 0.68 (95% CI, −1.34–2.71) for interventions in the family home and a statistically nonsignificant positive effect of 0.19 (95% CI, −0.06–0.45) for interventions in another location. There was a very large degree of heterogeneity of effects among studies in the subgroup of therapy in the family home (*τ* ^2^ = 2.07, *I* ^2^ = 97%) and a small degree of heterogeneity for therapy in another location (*τ* ^2^ = 0.02, *I* ^2^ = 15%). The CIs of the subgroups overlapped. There was no evidence to support the hypothesis that the effect differs by location.

##### 5.4.1.3 Parent outcomes

###### 5.4.1.3.1 Observed positive parenting behaviour

####### 5.4.1.3.1.1 Foster/adopted

**Figure 19:**
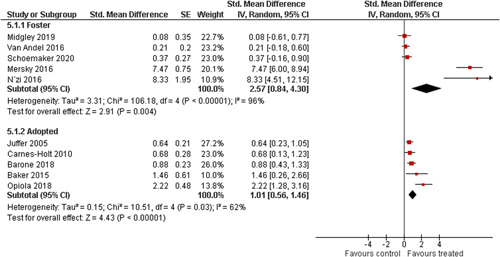
(Analysis 5.1) Forest plot of comparison: 5 subgroup parent outcomes, outcome: 5.1 adopted subgroup observed positive parent behaviour

Of the ten studies providing effect estimates of observed positive parenting behaviour, five used a sample of foster children only and five used a sample of adopted children only. The forest plot for the ten effect estimates is displayed in Figure 19. Pooled results for the two subgroups showed a statistically significant positive effect of 2.57 (95% CI, 0.84–4.30) for foster children and a statistically significant positive effect of 1.01 (95% CI, 0.56–1.46) for adopted children. There was a very large degree of heterogeneity of effects among studies in the subgroup of foster children (*τ* ^2^ = 3.31, *I* ^2^ = 96%) and a large degree of heterogeneity for adopted children (*τ* ^2^ = 0.15, *I* ^2^ = 62%). The CIs of the subgroups overlapped. There was no evidence to support the hypothesis that the effect differs by foster/adopted status.

####### 5.4.1.3.1.2 Age

**Figure 20:**
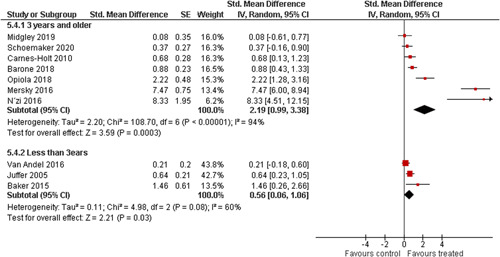
(Analysis 5.4) Forest plot of comparison: 5 subgroup parent outcomes, outcome: 5.4 age subgroup observed positive parent behaviour

Seven studies used a sample of children on average 3 years or older and three studies used a sample of children on average <3 years. The forest plot for the ten effect estimates is displayed in Figure 20. Pooled results for the two subgroups showed a statistically significant positive effect of 0.2.19 (95% CI, 0.99–3.38) for children on average aged 3 years or older and a statistically significant positive effect of 0.56 (95% CI, 0.06–1.06) for children on average <3 years old. There was a very large degree of heterogeneity of effects among studies in the subgroup of children on average aged 3 years or older (*τ* ^2^ = 2.20, *I* ^2^ = 94%) and a large degree of heterogeneity for children on average <3 years old (*τ* ^2^ = 0.11, *I* ^2^ = 60%). The CIs of the subgroups overlapped. There was no evidence to support the hypothesis that the effect differs by children's age.

####### 5.4.1.3.1.3 Location

**Figure 21:**
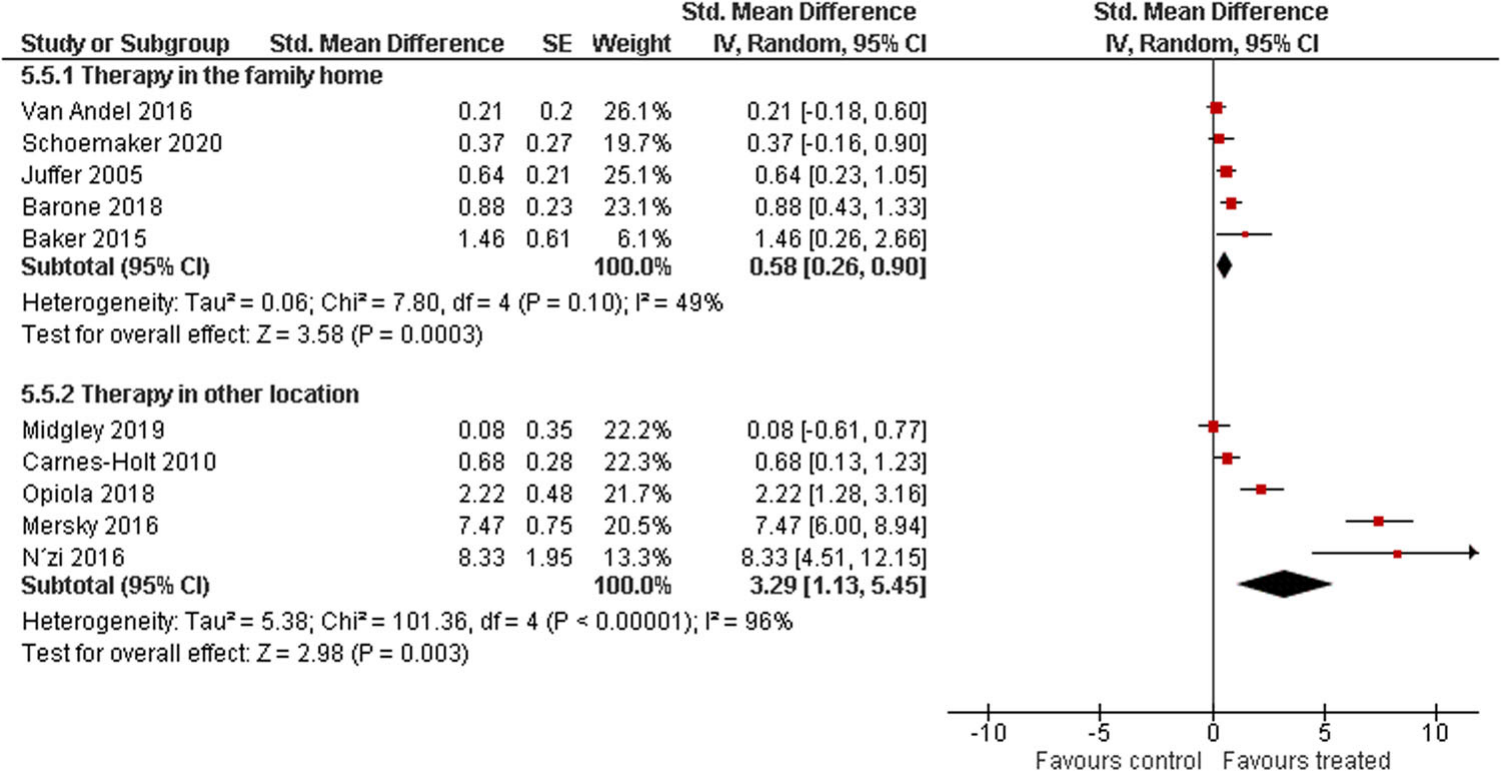
(Analysis 5.5) Forest plot of comparison: 5 subgroup parent outcomes, outcome: 5.5 location subgroup observed positive parent behaviour

Five studies analysed interventions in the family home and five analysed interventions in another location. The forest plot for the ten effect estimates is displayed in Figure 21. Pooled results for the two subgroups showed a statistically significant positive effect of 0.58 (95% CI, 0.26–0.90) for interventions in the family home and a statistically significant positive effect of 3.29 (95% CI, 1.13–5.45) for interventions in another location. There was some degree of heterogeneity of effects among studies in the subgroup of therapy in the family home (*τ* ^2^ = 0.06, *I* ^2^ = 49%) and a very large degree of heterogeneity for therapy in another location (*τ* ^2^ = 5.38, *I* ^2^ = 96%). The CIs of the subgroups did not overlap, hence we can not rule out that the effects differ by location.

###### 5.4.1.3.2 Parenting stress

####### 5.4.1.3.2.1 Foster/adopted

**Figure 22:**
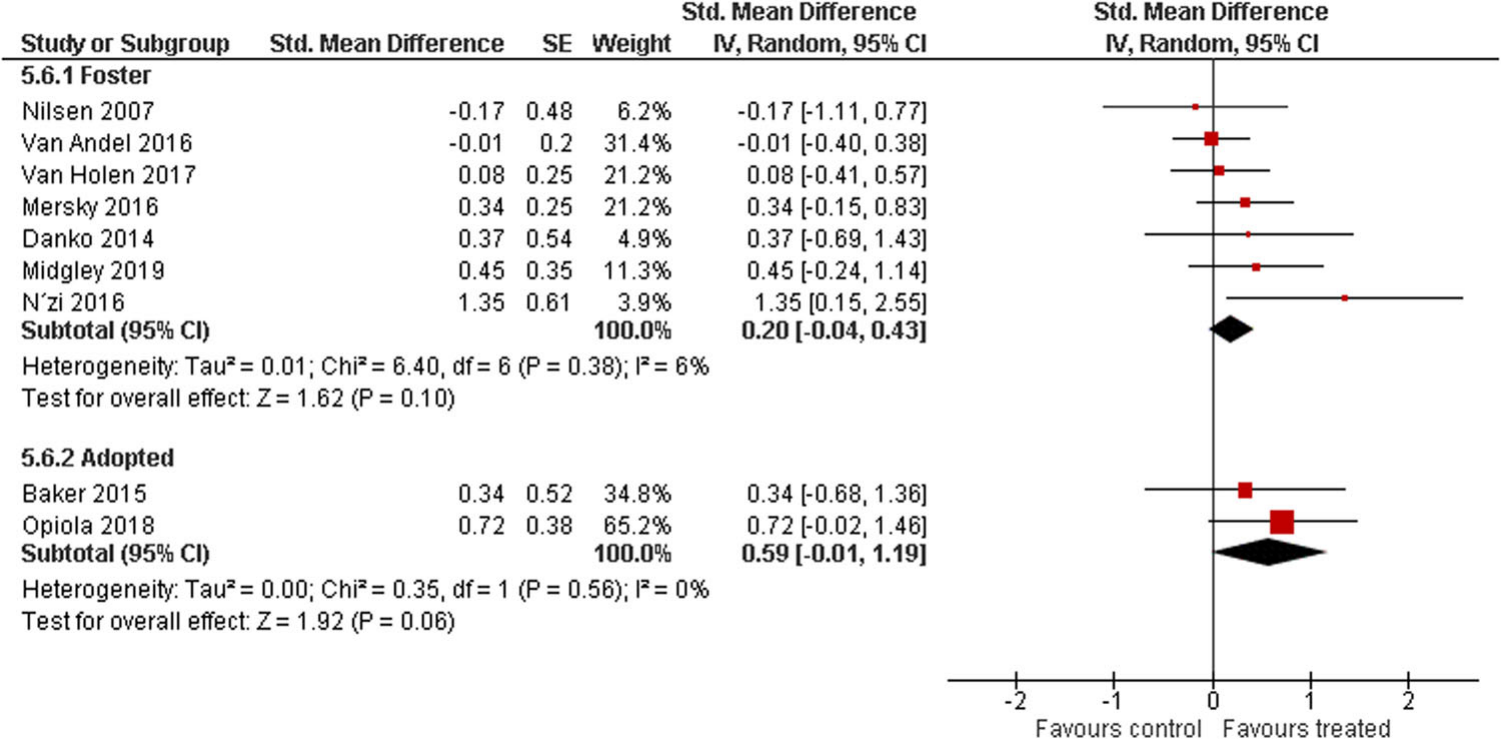
(Analysis 5.6) Forest plot of comparison: 5 subgroup parent outcomes, outcome: 5.6 adopted subgroup parenting stress

Of the nine studies providing effect estimates of parenting stress, seven used a sample of foster children only and two used a sample of adopted children only. The forest plot for the nine effect estimates is displayed in Figure 22. Pooled results for the two subgroups showed a statistically nonsignificant positive effect of 0.20 (95% CI, −0.04–0.43) for foster children and a statistically nonsignificant positive effect of 0.59 (95% CI, −0.01–1.19) for adopted children. There was a low degree of heterogeneity of effects among studies in the subgroup of foster children (*τ* ^2^ = 0.01, *I* ^2^ = 96%) and no heterogeneity for adopted children (*τ* ^2^ = 0.00, *I* ^2^ = 0%). The CIs of the subgroups overlapped. There was no evidence to support the hypothesis that the effect differs by foster/adopted status.

####### 5.4.1.3.2.2 Age

**Figure 23:**
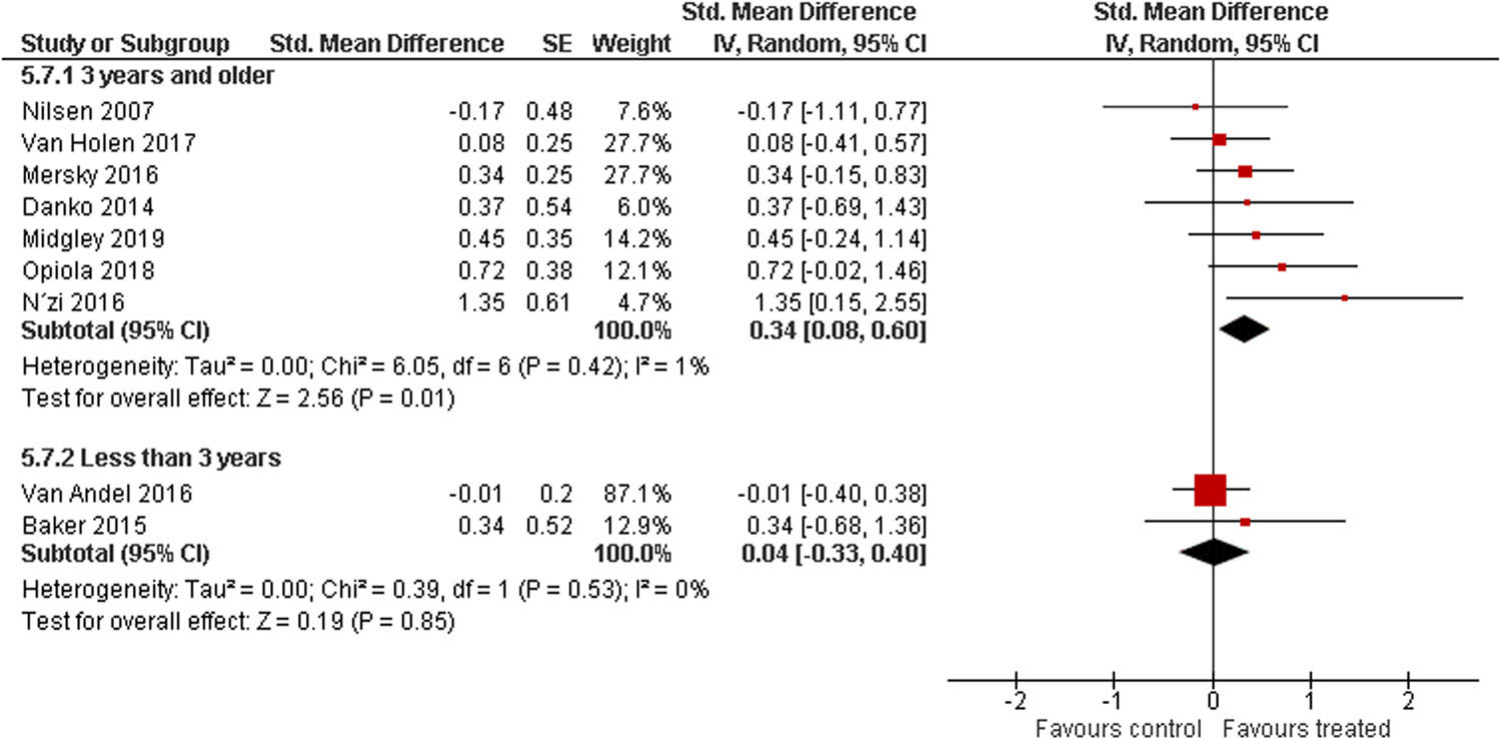
(Analysis 5.7) Forest plot of comparison: 5 subgroup parent outcomes, outcome: 5.7 age subgroup parenting stress

Seven studies used a sample of children on average 3 years or older and two studies used a sample of children on average <3 years. The forest plot for the nine effect estimates is displayed in Figure 23. Pooled results for the two subgroups showed a statistically significant positive effect of 0.34 (95% CI, 0.08–0.60) for children on average aged 3 years or older and a statistically nonsignificant positive effect of 0.04 (95% CI, −0.33–0.40) for children on average <3 years old. There was almost no heterogeneity of effects among studies in the subgroup of children on average aged 3 years or older (*τ* ^2^ = 0.00, *I* ^2^ = 1%) and no heterogeneity for children on average <3 years old (*τ* ^2^ = 0.0, *I* ^2^ = 0%). The CIs of the subgroups overlapped. There was no evidence to support the hypothesis that the effect differs by children's age.

####### 5.4.1.3.2.3 Location

**Figure 24:**
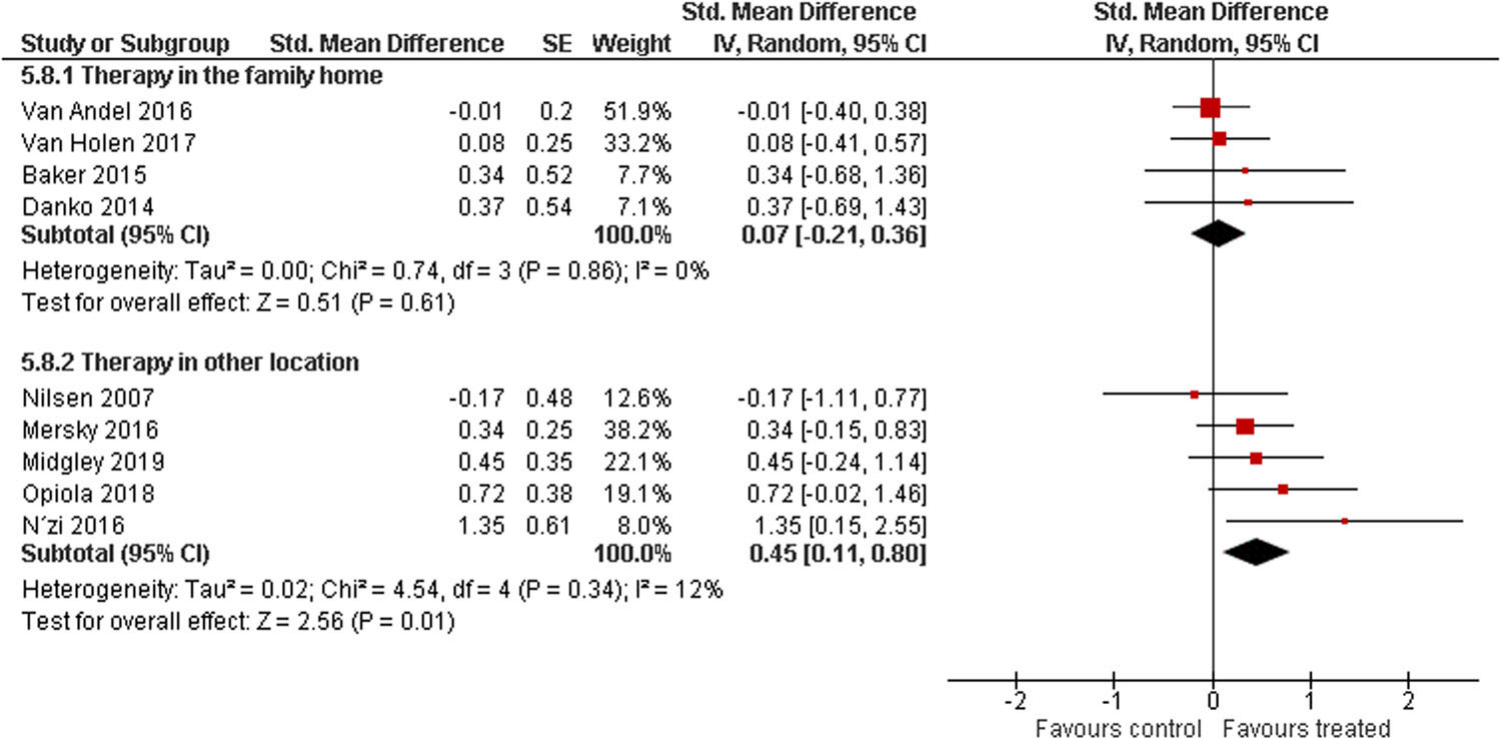
(Analysis 5.8) Forest plot of comparison: 5 subgroup parent outcomes, outcome: 5.8 location subgroup parenting stress

**Figure 25:**
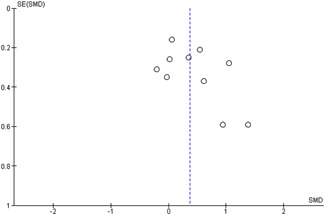
(Analysis 1.1) Funnel plot: child overall psychosocial adjustment

**Figure 26:**
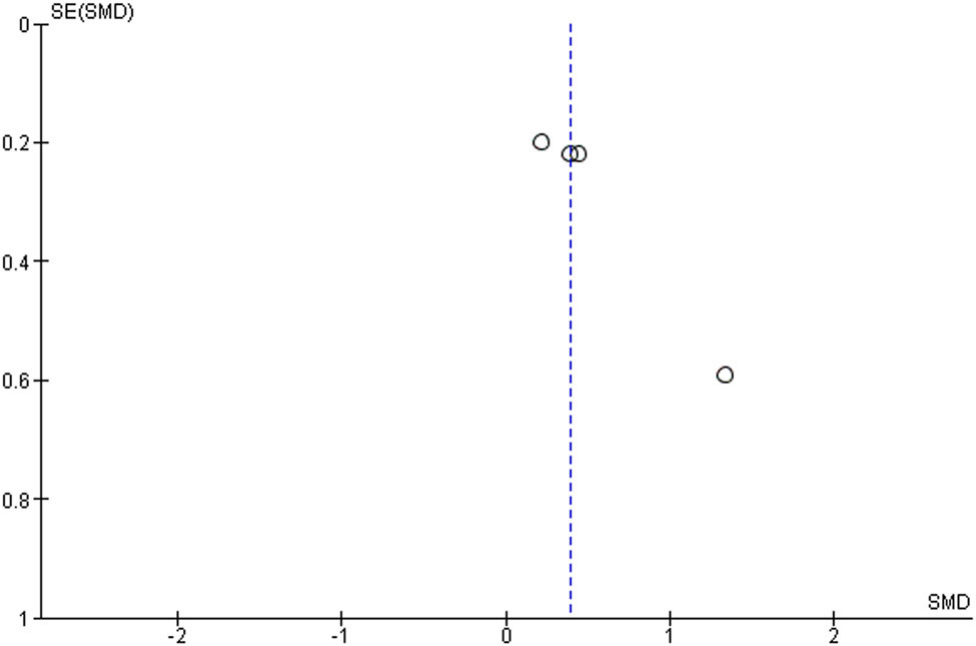
(Analysis 1.5) Funnel plot of comparison: 1 child outcomes, outcome: 1.5 observed positive child behaviour

**Figure 27:**
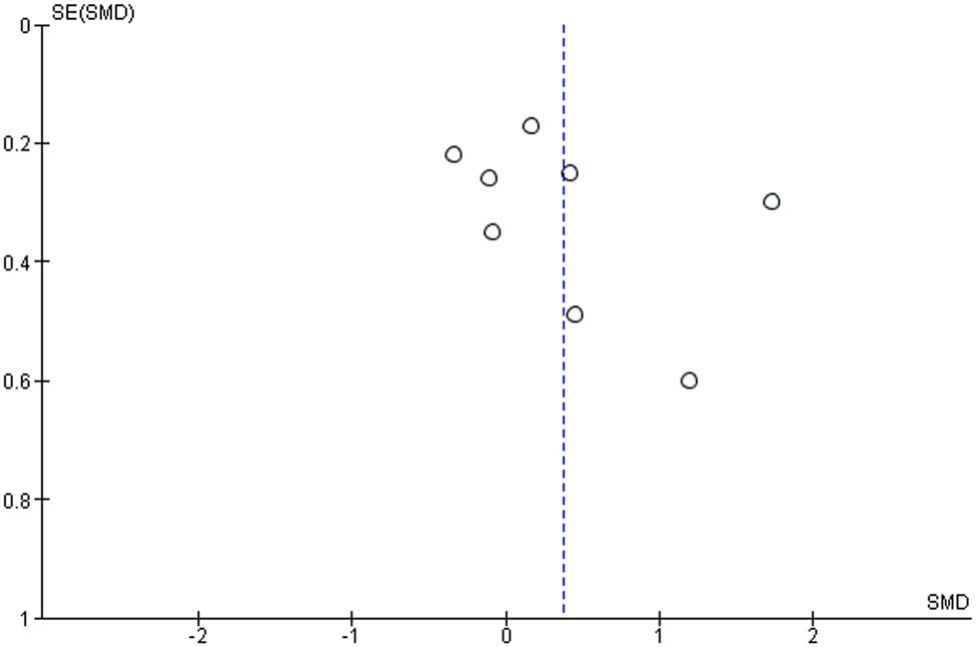
(Analysis 1.2) Funnel plot: child externalising behaviour

**Figure 28:**
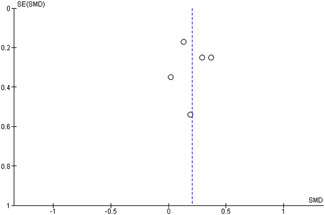
(Analysis 1.3) Funnel plot: 1 child Internalising symptoms

**Figure 29:**
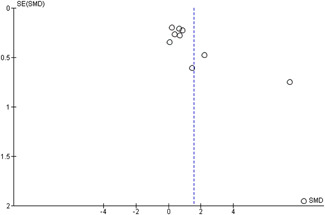
(Analysis 2.1) Funnel plot: observed positive parent behaviour

**Figure 30:**
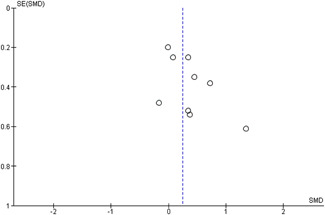
(Analysis 2.2) Funnel plot: parenting stress

Four studies analysed interventions in the family home and five analysed interventions in another location. The forest plot for the nine effect estimates is displayed in Figure 24. Pooled results for the two subgroups showed a statistically nonsignificant positive effect of 0.07 (95% CI, −0.21–0.36) for interventions in the family home and a statistically significant positive effect of 0.45 (95% CI, 0.11–0.80) for interventions in another location. There was no heterogeneity of effects among studies in the subgroup of therapy in the family home (*τ* ^2^ = 0.0, *I* ^2^ = 0%) and a very small degree of heterogeneity for therapy in another location (*τ* ^2^ = 0.02, *I* ^2^ = 12%). The CIs of the subgroups overlapped. There was no evidence to support the hypothesis that the effect differs by location (Figures 25, 26, 27, 28, 29, 30).

#### 5.4.2 Sensitivity analysis

We conduct sensitivity analyses by restricting the included studies to the RCTs and subsequently restricting the remaining RCTs to the most favourable rating for the overall risk of bias judgement. Sensitivity analysis is also conducted across domains of the risk of bias assessment, restricting studies to the most favourable rating for each domain. Sensitivity analysis is only conducted when there are more than two studies left in the analyses.

The analyses are performed separately by outcome, essentially replicating the meta‐analyses conducted in the section Effects of interventions.

##### 5.4.2.1 Child outcomes

###### 5.4.2.1.1 Overall psychosocial adjustment

**Figure 31:**
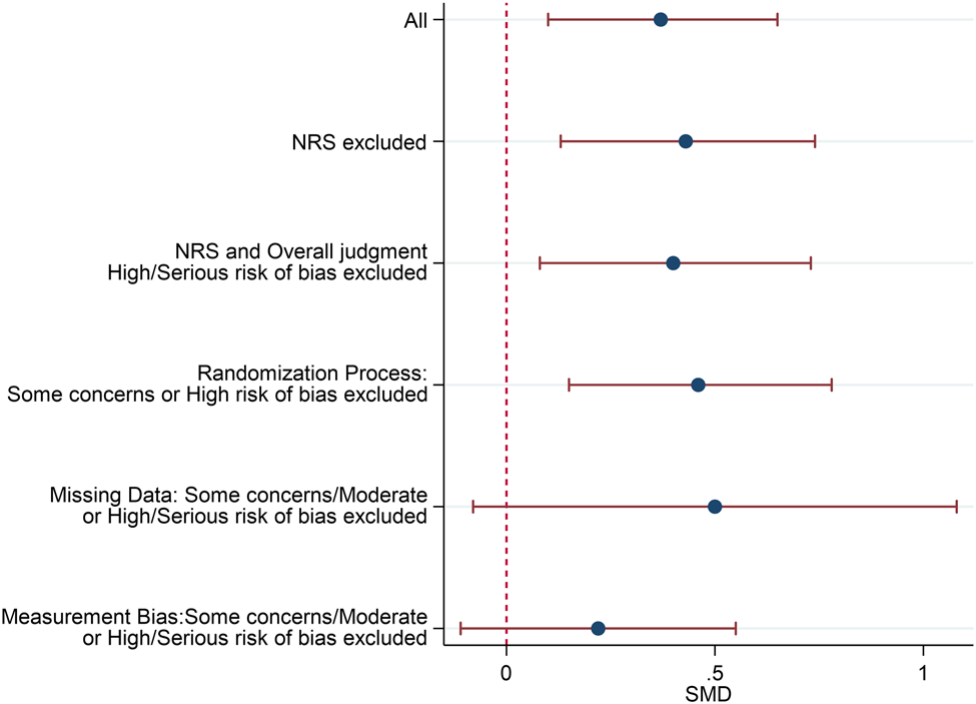
Sensitivity analysis, child overall psychosocial adjustment

The results of excluding the NRS study, and in addition the study with overall judgement High/Serious risk of bias and studies assessed Some concerns/Moderate or High/Serious risk of bias across the other domains of the risk of bias assessment are provided in Table 7 and displayed in a forest plot Figure 31. The child overall psychosocial adjustment outcome lost statistical significance when restricting the analysis to studies assessed Low risk of bias in the missing data domain and the measurement bias domain respectively. Otherwise, there were no appreciable changes in the results following removal of any of the studies.

**Table 7:** Sensitivity analysis

	Number of studies	Mean	95% CI	
Studies excluded	k	SMD	Lower	Upper
	10	0.37	0.10	0.65
NRS	9	0.43	0.13	0.74
NRS and overall judgement high/serious risk of bias	8	0.40	0.08	0.73
Randomisation process: some concerns or high risk of bias	2	0.46	0.15	0.78
Missing data: some concerns/moderate or high/serious risk of bias	5	0.50	−0.08	1.08
Measurement bias: some concerns/moderate or high/serious risk of bias	6	0.22	−0.11	0.55

*Note*: Restricting studies to RCTs, the most favourable rating on the overall risk of bias judgement and across domains of the risk of bias assessment. SMD with 95% CI. Reporting bias: some concerns/moderate or high/serious risk of bias excluded, only one study remained.

Abbreviations: CI, confidence interval; SMD, standardised mean difference.

###### 5.4.2.1.2 Externalising behaviour problems

**Figure 32:**
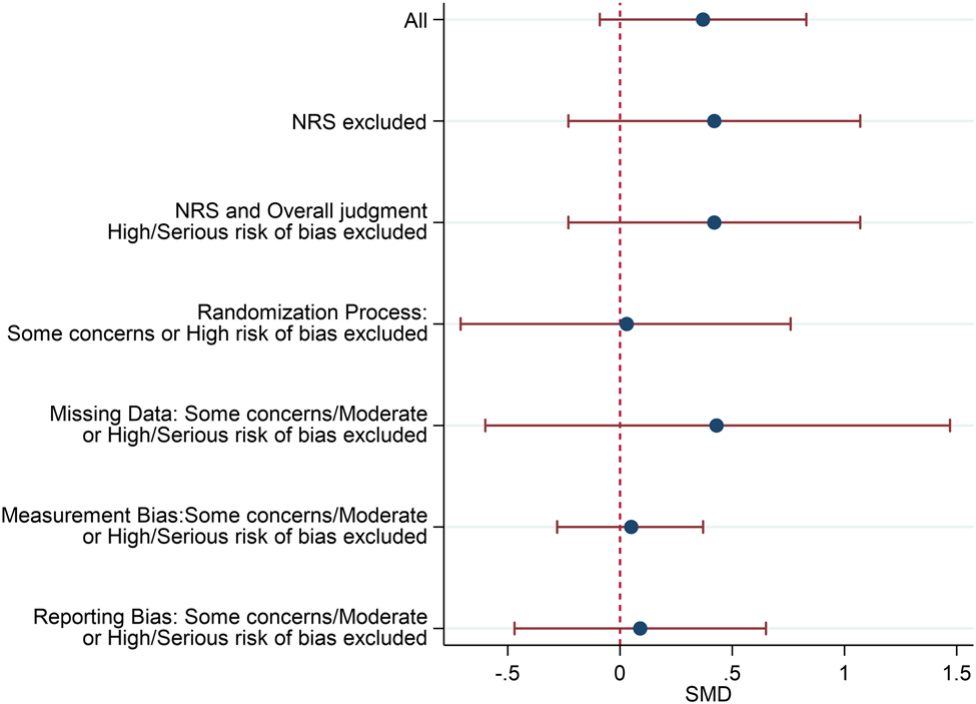
Sensitivity analysis, child externalising behaviour

The results of excluding the NRS studies (there were no RCTs with overall judgement High/Serious risk of bias) and studies assessed Some concerns/Moderate or High/Serious risk of bias across the other domains of the risk of bias assessment are provided in Table 8 and displayed in a forest plot, Figure 32. There were no appreciable changes in the results following removal of any of the studies.

**Table 8:** Sensitivity analysis

	Number of studies	Mean	95% CI	
Studies excluded	k	SMD	Lower	Upper
	8	0.37	−0.09	0.83
NRS	6	0.42	−0.23	1.07
NRS and overall judgement high/serious risk of bias	6	0.424	−0.23	1.07
Randomisation process: some concerns or high risk of bias	2	0.03	−0.71	0.76
Missing data: some concerns/moderate or high/serious risk of bias	4	0.43	−0.60	1.47
Measurement bias: some concerns/moderate or high/serious risk of bias	4	0.05	−0.28	0.37
Reporting bias: some concerns/moderate or high/serious risk of bias	2	0.09	−0.47	0.65

*Note*: Restricting studies to RCTs, the most favourable rating on the overall risk of bias judgement and across domains of the risk of bias assessment. SMD with 95% CI.

Abbreviations: CI, confidence interval; SMD, standardised mean difference.

###### 5.4.2.1.3 Internalising symptoms

**Figure 33:**
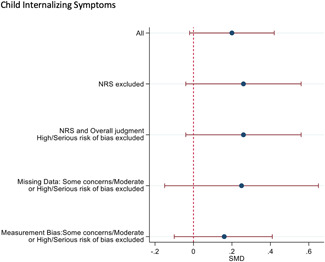
Sensitivity analysis, child internalising symptoms

The results of excluding the NRS study (there were no the studies with overall judgement High/Serious risk of bias) and studies assessed Some concerns/Moderate or High/Serious risk of bias across the other domains of the risk of bias assessment are provided in Table 9 and displayed in a forest plot, Figure 33. There were no appreciable changes in the results following removal of any of the studies.

**Table 9:** Sensitivity analysis

	Number of studies	Mean	95% CI	
Studies excluded	*k*	SMD	Lower	Upper
	5	0.20	−0.02	0.42
NRS	4	0.26	−0.04	0.56
NRS and overall judgment high/serious risk of bias	4	0.26	−0.04	0.56
Missing data: some concerns/moderate or high/serious risk of bias	2	0.25	−0.15	0.65
Measurement bias: some concerns/moderate or high/serious risk of bias	3	0.16	−0.10	0.41

*Note*: Restricting studies to RCTs, the most favourable rating on the overall risk of bias judgement and across domains of the risk of bias assessment. SMD with 95% CI. Randomisation Process: Some concerns or High risk of bias excluded, only one study remained; Deviation Bias: Some concerns/Moderate or High/Serious risk of bias excluded, only one study remained; Reporting Bias: Some concerns/Moderate or High/Serious risk of bias excluded, only one study remained.

Abbreviations: CI, confidence interval; SMD, standardised mean difference.

###### 5.4.2.1.4 Observed child attachment security

It was not possible to perform sensitivity analysis due to the limited number of studies reporting this outcome.

###### 5.4.2.1.5 Observed positive child behaviour

**Figure 34:**
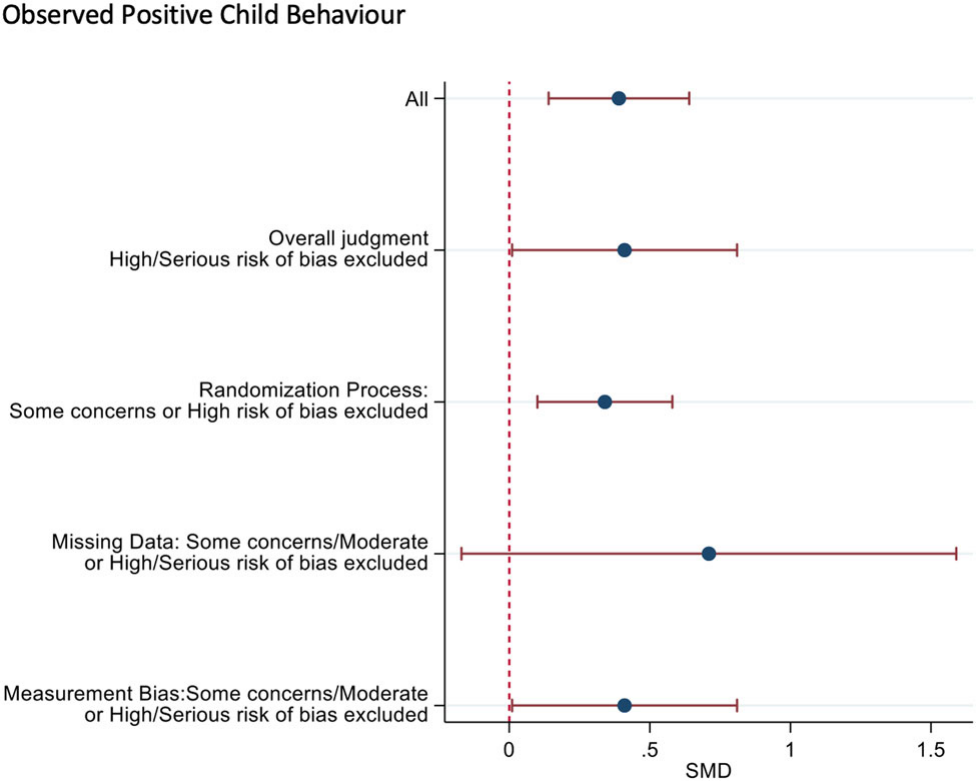
Sensitivity analysis, observed positive child behaviour

The results of excluding the studies with overall judgment High/Serious risk of bias and studies assessed Some concerns/Moderate or High/Serious risk of bias across the other domains of the risk of bias assessment are provided in Table 10 and displayed in a forest plot, Figure 34. The child observed positive behaviour outcome lost statistical significance when restricting the analysis to studies assessed Low risk of bias in the missing data domain. Otherwise, there were no appreciable changes in the results following removal of any of the studies.

**Table 10:** Sensitivity analysis

	Number of studies	Mean	95% CI	
Studies excluded	*k*	SMD	Lower	Upper
	4	0.39	0.14	0.64
Overall judgment high/serious risk of bias	3	0.41	0.01	0.81
Randomisation process: some concerns or high risk of bias	3	0.34	0.10	0.58
Missing data: some concerns/moderate or high/serious risk of bias	2	0.71	−0.17	1.59
Measurement bias: some concerns/moderate or high/serious risk of bias	3	0.41	0.01	0.81

*Note*: Restricting studies to the most favourable rating on the overall risk of bias judgment and across domains of the risk of bias assessment. SMD with 95% CI. NRS excluded, all studies were RCT; Deviation Bias: Some concerns/Moderate or High/Serious risk of bias excluded, only one study remained; Reporting Bias: Some concerns/Moderate or High/Serious risk of bias excluded, no studies remained.

Abbreviations: CI, confidence interval; SMD, standardised mean difference.

##### 5.4.2.2 Parent outcomes

###### 5.4.2.2.1 Observed positive parenting behaviour

**Figure 35:**
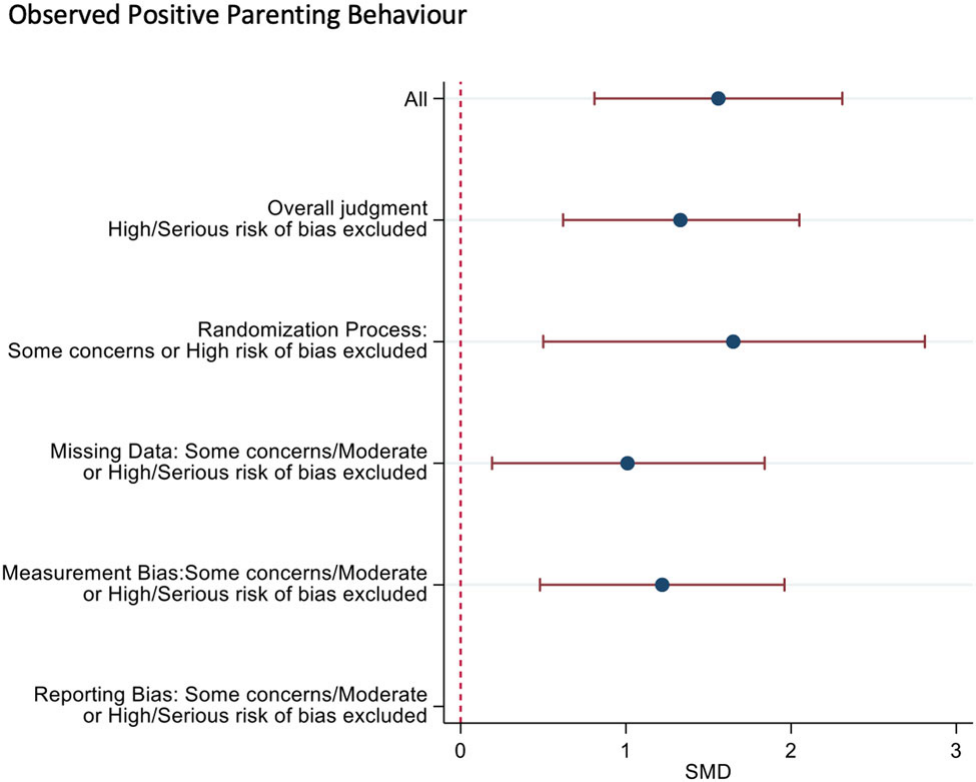
Sensitivity analysis, observed positive parenting behaviour

The results of excluding the studies with overall judgment High/Serious risk of bias and studies assessed Some concerns/Moderate or High/Serious risk of bias across the other domains of the risk of bias assessment are provided in Table 11 and displayed in a forest plot (Figure 35). There were no appreciable changes in the results following removal of any of the studies.

**Table 11:** Sensitivity analysis

	Number of studies	Mean	95% CI	
Studies excluded	k	SMD	Lower	Upper
	10	1.56	0.81	2.31
Overall judgment high/serious risk of bias	9	1.33	0.62	2.05
Randomisation process: some concerns or high risk of bias	5	1.65	0.50	2.81
Missing data: some concerns/moderate or high/serious risk of bias	4	1.01	0.19	1.84
Measurement bias: some concerns/moderate or high/serious risk of bias	8	1.22	0.48	1.96

*Note*: Restricting studies to the most favourable rating on the overall risk of bias judgment and across domains of the risk of bias assessment. SMD with 95% CI. NRS excluded, all studies were RCT; Deviation Bias: Some concerns/Moderate or High/Serious risk of bias excluded, only one study remained.

Abbreviations: CI, confidence interval; SMD, standardised mean difference.

###### 5.4.2.2.2 Parenting stress

**Figure 36:**
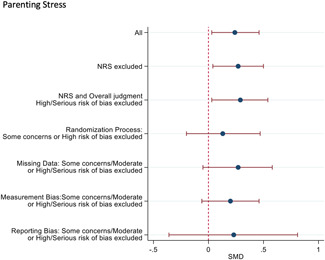
Sensitivity analysis, parenting stress

The results of excluding the NRS study, and in addition the study with overall judgment High/Serious risk of bias and studies assessed Some concerns/Moderate or High/Serious risk of bias across the other domains of the risk of bias assessment are provided in Table 12 and displayed in a forest plot (Figure 36).

**Table 12:** Sensitivity analysis

	Number of studies	Mean	95% CI	
Studies excluded	k	SMD	Lower	Upper
	9	0.24	0.03	0.46
NRS	8	0.27	0.04	0.50
NRS and overall judgment high/serious risk of bias	7	0.29	0.03	0.54
Randomisation process: some concerns or high risk of bias	2	0.13	−0.20	0.47
Missing data: some concerns/moderate or high/serious risk of bias	5	0.27	−0.05	0.58
Measurement bias: some concerns/moderate or high/serious risk of bias	5	0.20	−0.06	0.46
Reporting bias: some concerns/moderate or high/serious risk of bias	2	0.23	−0.36	0.81

*Note*: Restricting studies to RCTs, the most favourable rating on the overall risk of bias judgment and across domains of the risk of bias assessment. SMD with 95% CI.

Abbreviations: CI, confidence interval; SMD, standardised mean difference.

The parent stress outcome lost statistical significance when restricting the analysis to studies assessed Low risk of bias in the randomisation process, missing data domain, the measurement bias domain and reporting bias domain respectively. Otherwise, there were no appreciable changes in the results following removal of any of the studies.

###### 5.4.2.2.3 Parental depressive symptoms

It was not possible to perform sensitivity analysis due to the limited number of studies reporting this outcome.

##### 5.4.2.3 Follow‐up

It was not possible to perform sensitivity analyses due to the limited number of studies reporting any of the outcomes at follow up.

##### 5.4.2.4 Cluster

Finally, we investigated the sensitivity of including the cluster randomised trial (Opiola 2018) using an ICC equal to one. The results of varying the ICC are provided in Table 13. There were no appreciable changes in the results.

**Table 13:** Sensitivity analysis

Outcome	Child overall psychosocial adjustment	Observed positive parenting	Parenting stress
ICC	Pooled SMD [95% CI]		
1	0.37 [0.10, 0.65]	1.56 [0.81, 2.31]	0.24 [0.03, 0.46]
0.9	0.37 [0.10, 0.65]	1.56 [0.81, 2.31]	0.25 [0.03, 0.46]
0.8	0.37 [0.10, 0.65]	1.56 [0.81, 2.31]	0.25 [0.03, 0.47]
0.7	0.37 [0.10, 0.65]	1.56 [0.81, 2.32]	0.25 [0.03, 0.47]
0.6	0.37 [0.10, 0.65]	1.57 [0.81, 2.32]	0.25 [0.03, 0.47]
0.5	0.38 [0.10, 0.65]	1.57 [0.82, 2.32]	0.26 [0.04, 0.48]
0.0	0.38 [0.11, 0.65]	1.57 [0.82, 2.33]	0.28 [0.05, 0.51]

*Note*: Varying ICC in the cluster randomised trial.

**Table 14:** Other outcomes

Author/year	Measure	Outcome	SMD	Lower CI	Upper CI
Baker (2015)	Emotional Availability Self‐Report	Child Capacity to Involve Parent	0.84	−0.24	1.92
Baker (2015)	Emotional Availability Self‐Report	Mutual Attunement	1.57	0.35	2.79
Baker (2015)	Emotional Availability Self‐Report	Affect Quality	0.06	−0.96	1.08
Baker (2015)	Emotional Availability Self‐Report	Hostility	1.35	0.19	2.51
Baker (2015)	Emotional Availability Self‐Report	Intrusiveness	0.16	−0.86	1.18
Baker (2015)	Attachment Q‐Sort	Security	1.65	0.42	2.88
Barone (2019)	Indiscriminate Friendliness Interview	Childs indiscriminate friendliness	0.14	−0.29	0.57
Bywather (2011)	Eyberg Child Behaviour Inventory	Intensity	−0.27	−0.88	0.34
Bywather (2011)	Strengths and Difficulties Questionnaire	Hyperactive	0.22	−0.39	0.83
Bywather (2011)	Arnold, Parenting Scale		0.35	−0.26	0.96
Mersky (2016)	Eyberg Child Behavior Inventory (ECBI).	ECBI intensity	0.59	0.10	1.08
Mersky (2016)	Eyberg Child Behavior Inventory (ECBI).	ECBI problem	0.63	0.14	1.12
Midgley (2019)	Revised child anxiety and depression scale (self report)	Child anxiety and depression	−0.16	−0.85	0.53
Midgley (2019)	Revised child anxiety and depression scale (foster carer report)	Child anxiety and depression	−0.28	−0.97	0.41
Midgley (2019)	Strengths and Difficulties Questionnaire Self‐report	Externalising subscale	−0.05	−0.93	0.83
Midgley (2019)	Strengths and Difficulties Questionnaire Self‐report	Internalising subscale	0.11	−0.77	0.99
Midgley (2019)	Strengths and Difficulties Questionnaire Self‐report	Total problems	0.03	−0.85	0.91
Midgley (2019)	Brief parental self‐efficacy scale	Parental self‐efficacy	−0.39	−1.08	0.30
N'zi (2016)	Child–Parent Relationship Scale	Positive Aspects of the Relationship	0.84	−0.28	1.96
Nilsen (2007)	The Behavioral Assessment System for Children	Conduct	0.52	−0.44	1.48
Nilsen (2007)	The Behavioral Assessment System for Children	Aggression	0.15	−0.79	1.09
Nilsen (2007)	The Behavioral Assessment System for Children	Hyperactivity	0.56	−0.40	1.52
Nilsen (2007)	Adult–Adolescent Parenting Inventory.	Inappropriate expectations	0.08	−0.86	1.02
Nilsen (2007)	Adult–Adolescent Parenting Inventory.	Lack of empathy	0.02	−0.92	0.96
Nilsen (2007)	Adult–Adolescent Parenting Inventory.	Parent–child role reversal	−0.20	−1.14	0.74
Nilsen (2007)	Adult–Adolescent Parenting Inventory.	Power differential	0.27	−0.69	1.23
Razuri (2016)	Strengths and Difficulties Questionnaire	Prosocial behavior	0.08	−0.23	0.39
Razuri (2016)	Trauma Symptoms Checklist for Young Children	Anger/Aggression	−0.01	−0.32	0.30
Razuri (2016)	Trauma Symptoms Checklist for Young Children	Posttraumatic Stress—Intrusion	−0.01	−0.32	0.30
Razuri (2016)	Trauma Symptoms Checklist for Young Children	Posttraumatic Stress—Avoidance	−0.08	−0.39	0.23
Razuri (2016)	Trauma Symptoms Checklist for Young Children	Posttraumatic Stress—Arousal	0.13	−0.18	0.44
Razuri (2016)	Trauma Symptoms Checklist for Young Children	Dissociation	0.20	−0.13	0.53
Razuri (2016)	Trauma Symptoms Checklist for Young Children	Sexual concerns	−0.21	−0.54	0.12
Razuri (2016)	Trauma Symptoms Checklist for Young Children	Posttraumatic Stress—Total	0.02	−0.29	0.33
Schoemaker (2020)	Erickson scale for supportive presence (based on video tapes)	Sensitive discipline	0.43	−0.10	0.96
Schoemaker (2020)	Questionnaire Attitudes towards Parenting	Attitudes towards sensitivity	0.36	−0.17	0.89

**Table 15:** Other outcomes follow up

Author (year)	Measure	Outcome	SMD	Lower CI	Upper CI
Barone (2019)	Emotional Availability scales, 4th Edition (EAs—Biringen, 2008) by videotaping	Emotional availability: two scales of child's behaviour: responsiveness and involvement.	0.76	0.31	1.21
Barone (2019)	Indiscriminate Friendliness Interview (Chisholm, 1998; Chisholm, Carter, Ames, & Morison, 1995)	Childs indiscriminate friendliness (follow‐up)	0.07	−0.38	0.52
Midgley (2019)	Strengths and Difficulties Questionnaire (foster care report)	Total problems	−0.27	−0.96	0.42
Midgley (2019)	Brief parental self‐efficacy scale	Parental self‐efficacy	0.26	−0.43	0.95
Midgley (2019)	Revised child anxiety and depression scale (self report)	Child anxiety and depression	−0.25	−0.94	0.44
Midgley (2019)	Revised child anxiety and depression scale (foster care report)	Child anxiety and depression	−0.47	−1.16	0.22

#### 5.4.3 Reporting bias

We constructed funnel plots for analyses with more than four studies, which was the case for the outcomes: child overall psychosocial adjustment, child externalising symptoms, child internalising symptoms, observed positive parenting and parenting stress, The funnel plots were visually examined and there were no striking imbalances, except for the outcome: observed positive parenting. The imbalance in this plot may be due to two very large effect sizes, and the limited number of studies does not permit any definitive conclusions regarding publication bias.

## 6 DISCUSSION

### 6.1 Summary of main results

his review focused on the effect of parenting interventions based on attachment theory on child and parent outcomes. The review summarises findings from 44 studies reporting on 27 different samples (19 randomised trials and 8 nonrandomised studies). The meta‐analyses are based on 24 studies reporting on 16 samples (14 randomised trials and two nonrandomised studies).

#### 6.1.1 Child outcomes

The available evidence does suggest that parenting interventions based on attachment theory increase the overall psychosocial adjustment of children in foster and adoptive families at the end of the intervention. We found a statistically significant positive effect of moderate size. The weighted average effect (using the ten studies reporting this outcome) is a SMD of 0.37 (95% CI, 0.10–0.65). To ease interpretation we translate the SMD into the probability‐of‐benefit (POB) statistic. POB is also known as the Common Language effect size statistic (CL) defined in McGraw and Wong (1992). To calculate the POB for continuous data, we first compute: *Z* = SMD/√2. This *Z* statistic is normally expressed as a standard normal distribution, and the POB is computed as the probability that a randomly selected standard normal variable is less than *Z*.

Measured as the POB statistic, defined as the probability that a randomly selected score from the treated population of children would be greater than a randomly selected score from the comparison population of children, the overall psychosocial adjustment of children POB was 0.60. A SMD of 0.37, therefore, corresponds to a 60% chance that a randomly selected score of a child from the treated population is greater than the score of a randomly selected child from the comparison population. Note that this probability should be compared to a fifty‐fifty chance, which is 50%, had the intervention been ineffective. The lower and upper 95% CI corresponds to 53% and 68%, respectively, chance of a randomly selected score of the treated being higher than a score from the comparison population.

Regarding the effect of attachment‐based parenting interventions on decreasing children's externalising symptoms at the end of intervention, we found a positive effect of moderate size although not statistically significant and there is a substantial degree of heterogeneity between studies. The weighted average effect (using the eight studies reporting this outcome) is a SMD of 0.37 (95% CI, −0.09–0.83). This corresponds to a 60% chance that a randomly selected score of a child from the treated population is better (note that a higher score for this outcome means less externalising symptoms) than the score of a randomly selected child from the comparison population. The lower and upper 95% CI corresponds to 47% and 72%, respectively.

The analysis using the five studies reporting children's internalising symptoms at the end of intervention indicated that there is a small positive but statistically nonsignificant effect of attachment‐based parenting interventions (SMD = 0.20 (95% CI, −0.02–0.42)). However, given the relatively few studies, some caution is needed in making an assumption that attachment‐based parenting interventions do not decrease children's internalising symptoms. This corresponds to a 56% chance that a randomly selected score of a child from the treated population is better (note that a higher score for this outcome means fewer internalising symptoms) than the score of a randomly selected child from the comparison population. The lower and upper 95% CI corresponds to 49% and 62%, respectively.

Three studies analysed the effects of attachment‐based interventions on the observed attachment security of foster and adopted children. We were unable to conduct a meta‐analysis using attachment disorganisation as an outcome as this way of analysing attachment data was only available in one study which was already used in the meta‐analysis using attachment security as an outcome. The analysis of the three studies reporting on security indicated a positive but statistically nonsignificant effect (SMD = 0.59 (95% CI, −0.40–1.57). This corresponds to a 66% chance that a randomly selected score of a child from the treated population is better than the score of a randomly selected child from the comparison population. The lower and upper 95% CI corresponds to 39% and 87%, respectively. Thus, evidence on the effect of attachment‐based parenting interventions on attachment security in foster and adopted children was inconclusive due to too few studies.

The available evidence does suggest that attachment‐based parenting interventions increase positive child behaviours after the intervention based on independent observation. We found a statistically significant positive effect of moderate size. The weighted average effect (using the four studies reporting this outcome) is a SMD of 0.39 (95% CI, 0.14–0.64). A SMD of 0.39 corresponds to a 61% chance that a randomly selected score of a child from the treated population is greater than the score of a randomly selected child from the comparison population. The lower and upper 95% CI corresponds to 54% and 67%, respectively, chance of a randomly selected score of the treated being higher than a score from the comparison population of children.

#### 6.1.2 Parent outcomes

Attachment‐based parenting interventions also increase positive parent behaviours after the intervention based on independent observation. We found a statistically significant positive large effect. The weighted average effect (using the ten studies reporting this outcome) is a SMD of 1.56 (95% CI, 0.81–2.31). This corresponds to an 87% chance that a randomly selected score of a parent from the treated population is greater than the score of a randomly selected parent from the comparison population. The lower and upper 95% CI corresponds to 72% and 95%, respectively, chance of a randomly selected score of the treated being higher than a score from the comparison population of parents. Note that there is a substantial amount of heterogeneity between studies. Some caution is needed in making an assumption that there is a single true effect from attachment‐based parenting interventions on positive parent behaviours.

Attachment‐based parenting interventions decrease parenting stress at the end of intervention as reported by foster/and adoptive parents although the effect is small. The weighted average effect (using the nine studies reporting this outcome) is a SMD of 0.24 (95% CI, 0.03–0.46), corresponding to a 57% chance that a randomly selected score of a parent from the treated population is better (note that a higher score for this outcome means fewer symptoms of stress) than the score of a randomly selected parent from the comparison population. The lower and upper 95% CI corresponds to 51% and 63%, respectively.

Due to too few studies, evidence on the effect of attachment‐based parenting interventions on parental depressive symptoms was inconclusive.

#### 6.1.3 Follow up

Furthermore, due to too few studies evidence on the long‐term (3–6 months after the intervention was finished) effects of attachment‐based parenting interventions on any outcomes was inconclusive.

#### 6.1.4 Subgroup

It was possible to assess the impact of two child characteristics (children being adopted vs. foster children and child age <3 years vs. 3 years or older), and one intervention characteristic (therapy in the family home vs. other locations). We only performed subgroup analysis if there were at least two studies in each subgroup (child overall psychosocial adjustment, child externalising symptoms, observed positive parenting, and parenting stress).

We found no evidence to suggest that the effect of attachment‐based parenting interventions differ between the two types of child characteristics on any outcomes. Further, we found no evidence to suggest that the effect of attachment‐based parenting interventions differ between the location of the therapy on the outcomes; child externalising symptoms and parenting stress. We can not rule out that the effects differ by location for the outcomes child overall psychosocial adjustment and observed positive parenting. However, it is unclear if interventions in the family home or in other locations (typically a clinical setting) are more beneficial, as the results of the single factor subgroup analyses suggested opposite impacts for parent and child outcomes. More specificallly results of the analysis suggest, that for child outcomes therapy in the family home is more beneficial, whereas for parent outcomes therapy in othre locations than the family home is more beneficial. Further, note that as we found these differences in single factor subgroup analyses, we cannot be sure that the differences were not caused by other factors than location.

### 6.2 Overall completeness and applicability of evidence

In this review, we included a total of 24 studies in the data synthesis. This number is relatively low compared to the large number of studies (44) meeting the inclusion criteria. The reduction was caused by three different factors. Fifteen studies were judged to have a critically high risk of bias and, in accordance with the protocol, we excluded these from the data synthesis on the basis that they would be more likely to mislead than inform on the size of the effect of the intervention. Five of the 44 studies did not report effect estimates or provide data that would allow the calculation of an effect size.

If all the 44 studies had provided an effect estimate with a lower risk of bias, the final list of useable studies in the data synthesis would have been larger which, in turn, would have provided a more robust literature on which to base conclusions.

We analysed all available child and parent outcomes. However, the small number of studies reporting on child attachment makes us reluctant to draw conclusions, and thus more research is needed.

We found no strong indication of publication bias.

Although we searched for studies including children aged 0–17 most of the studies included in the analyses consisted of relatively young children. The mean age of the children in the included studies was 5.15 years ranging from 0.62 to 10.65 years. The majority of children had been placed in care at a relatively young age. The mean age of placement was 2.31 years ranging from 0.25 to 4.8 years. This is not surprising as attachment based interventions traditionally are offered to families with young children.

Findings from the present review are relevant to all clinicians working with children and parents in foster and adoptive families and suggest that using this type of intervention with this population has clinically relevant positive short‐term effects. The findings from the meta‐analysis of the outcomes: observed positive parent behaviour, observed positive child behaviour and the overall psychosocial adjustment of the children suggest, that although there is no rigorous evidence, attachment‐based interventions may also promote the longer term psychosocial adjustment of children and promote positive family coping. The fact that there were so few studies reporting on the long term effects of attachment‐based interventions on all outcomes beyond the 3–6 months follow up is not surprising as longitudinal research is costly and difficult to carry out. However, as stated in the introduction the consequences of placement breakdown to both the children, parents, and society are grim and thus more research should explore this issue further.

### 6.3 Quality of the evidence

Overall the risk of bias in the majority of included nonrandomised studies was high. Six of nine nonrandomised studies were judged overall to be at critical risk of bias and the remaining three were rated serious risk of bias overall. The included studies reporting on RCTs were of lower risk of bias; nine were judged overall to be at critical risk of bias and the majority (23) were rated Some concerns and the remaining three high risk of bias. The risk of bias in randomised studies was examined using Cochranes revised risk of bias tool, RoB 2 (Higgins et al., 2019), and the risk of bias in nonrandomised studies, using the model ROBINS‐I, developed by members of the Cochrane Bias Methods Group and the Cochrane Non‐Randomised Studies Methods Group (Sterne et al., 2016). We attempted to manage the level of the quality of the evidence in this review by excluding studies judged to be at critical risk of bias using these tools. We believe this process excluded those studies that are more likely to mislead than inform.

Six studies reported the results of an American randomized trial using the ABC intervention: Dozier et al. (2006, 2008, 2009), Bick and Mary (2013), Lewis‐Morrarty et al. (2012), and Lind et al. (2017). One publication only reported one outcome, which was not relevant to the present review, and the remaining five studies were rated as in critical risk of bias due to a number of problems primarily caused by a lack of information regarding the number of participants and time points for measurement. We attempted to contact authors, which was successful in one case. However, the author could only provide the means and SDs for a subsample and only from one time point in which the attrition from the trial as reported in the publication exceeded 50%, and thus we deemed the risk of reporting bias to be critical in these five publications.

The fact that only three of the RCT's had a published a priori protocol or a priori analysis plan is concerning, and may suggest that studies within this field are of lower quality than what would be expected, and thus points to the need for increased methodological rigour in future research. However especially for the older studies, it may also just reflect that publishing protocols is or was not standard procedure/expected in this area.

The included studies were relatively small with an average of 72 participants ranging from 14 to 256. In many of the meta‐analyses most of the individual study results have CIs crossing 0 making them insignificant. As the individual studies are relatively small and the highest number of studies included in a meta‐analysis is 10 the total number of participants is still limited.

We performed sensitivity analyses (where possible) to check whether the obtained results are robust across methodological quality. Although the statistically significant pooled outcomes lost significance in a few instances, the overall conclusions did not change.

There was overall consistency in the direction of effects at end of intervention. Very few the primary study results were negative, that is, most of the effect sizes favoured the treated group. There was a high degree of heterogeneity between studies in some of the analyses, especially in the analyses of child outcomes. In two of five analyses of child outcomes, there were no or a small degree of heterogeneity whereas in two of three analysis of parent outcomes there was no or a small degree of heterogeneity.

Concerning child outcomes, evidence was less consistent for assessments taken at follow up times, 3–6 months post intervention. In addition, for both child outcomes and parent outcomes, there was a high degree of heterogeneity between the studies at follow up and few studies reported data at follow up.

### 6.4 Potential biases in the review process

We performed a comprehensive electronic database search, combined with grey literature searching, and hand searching of key journals. All citations were screened by two independent screeners from the review team, and one review author (NTD) assessed all included studies against inclusion criteria.

We believe that all the publicly available studies on the effect of attachment‐based parenting interventions with foster and adoptive families up to the censor date were identified during the review process. However, 21 references were not obtained in full text. Despite attempts to contact the author(s) of all studies with insufficient information, we are still awaiting answers with regard to six studies.

We were unable to comment on the possibility of publication bias as at most ten studies were included in the same meta‐analysis. Thus, we cannot rule out that there are still some missing studies.

We believe that there are no other potential biases in the review process as two members of the review team (JH, TEJ, KGE, and ANDA) independently coded the included studies. Any disagreements were resolved by discussion. Further, decisions about the inclusion of studies were made by two members of the review team (TEJ, KGE, and ANDA) and at least one review author (TRF and NTD). Assessment of study quality and numeric data extraction was made by one review author (TRF/NTD/MP) or MKT and was checked by a second review author and two members of the review team (TEJ, KGE, and ANDA).^2^

### 6.5 Agreements and disagreements with other studies or reviews

In a meta‐analysis on the effectiveness of preventative attachment interventions on parental sensitivity and infant attachment for at risk populations (*k* = 70), results suggest that interventions are rather effective at increasing parental sensitivity (*d* = 0.33) and/or infant attachment (*d* = 0.20). (Bakermans‐Kranenburg et al., 2003). In a systematic review co‐registered at Cohrane and Campbell, Barlow et al. (2015) explored the effects of attachment‐based parent‐infant psychotherapy on parental and infant mental health. This review focused on infants aged 0–24 months within vulnerable families (defined as families in which parents were suffering from mental health issues, drug/alcohol abuse, or were victims of domestic violence). Findings from this review suggest that parent‐infant psychotherapy is a promising approach in terms of improving infant attachment security in high‐risk families. However, there were no significant differences compared with no treatment or treatment‐as‐usual for other parent‐based or relationship‐based outcomes.

In the present review, we found a larger effect size on positive parenting behaviour than what was reported by Bakermans‐Kranenburg et al. (2003) and, in contrast to Barlow et al. (2015) we did find other positive effects of attachment‐based interventions when compared to treatment as usual. These differences may perhaps be explained by a difference in motivation and socioemotional resources between at‐risk biological parents and the population of the present review.

The fact that the reviews by Bakermans‐Kranenburg et al. and by Barlow et al. were able report a positive effect on child attachment outcomes is interesting and emphasises the need for more research on the effects of attachment‐based interventions on attachment outcomes in foster care and adoption, given that we were only able to extract this outcome from three studies.

Juffer et al. (2017) provides a review and meta‐analysis of the effects of Video‐feedback Intervention to promote Positive Parenting and Sensitive Discipline (VIPP‐SD), which is an intervention based partially on attachment theory with various populations of at‐risk parents and vulnerable children (*k* = 12). In the review, positive effects of VIPP‐SD were found on measures of sensitive parenting and socio‐emotional child outcomes, which is similar to the findings from the present review.

Schoemaker et al conducted a meta‐analytic review (*k* = 53) examining the effects of all types of parenting interventions in foster care and adoption on eight types of outcomes (Schoemaker et al., 2019). Results show positive effects on four parenting outcomes (sensitive parenting, dysfunctional discipline, parenting knowledge and attitudes, and parenting stress, and on one child outcome (behaviour problems), whereas the review did not find effects for attachment security, child diurnal cortisol levels, or placement disruptions. The findings regarding parental outcomes are broader in scope than what we find in this review, but the finding regarding parental sensitivity is similar to the findings from the present review. These findings may be interpreted as an indication that other types of interventions are as effective as attachment‐based interventions on parental behaviour outcomes. Finally, it is also interesting to note that Schoemaker et al. did not find positive effects on child attachment outcomes, and thus it is possible that child attachment security is difficult to change within the relatively short period of time that parenting interventions typically last. It is possible that the positive effects on parenting behaviours, if sustained over time, may lead to possible improvement in child attachment security and a decrease in child disorganised attachment over time, which again highlights the need for more longitudinal research.

## 7 AUTHORS' CONCLUSIONS

### 7.1 Implications for practice

Findings from this review indicate that parenting interventions based on attachment theory increase the overall psychosocial adjustment of children in foster and adoptive families as reported by their caregivers after the intervention. Attachment‐based parenting interventions also increase positive parent/child interactional behaviours post intervention based on independent observation and decrease self‐reported parenting stress. In summary these findings suggest, that attachment‐based parenting interventions have benefits for foster and adopted children and for foster and adoptive parents.

Results from the moderator analyses tentatively suggest that the effects of attachment‐based parenting interventions may differ depending on whether the intervention takes place in the family home or in another location (typically a clinical setting). However, based on the present review it is undetermined which location is more beneficial as results were divergent for parent and child outcomes and we were not able to take into consideration other factors that may have an impact on the effect in the analysis.

The evidence regarding the effect of attachment‐based parenting interventions on decreasing parental depressive symptoms was inconclusive. Findings from a review of all types of interventions in foster care and adoption (Schoemaker et al., 2019) suggest that other types of interventions are as effective as attachment interventions on parental behaviour outcomes.

In the present review, only three studies explored the effects of attachment‐based interventions on measures of child attachment security. This is puzzling given the nature of the interventions. However, it may reflect a lack of reliable and well‐established methods for measuring attachment representations in older children, where it is not possible to use behavioral measures such as the Strange Situation Procedure. Recent research suggests that story stem measures may be a reliable clinical tool for measuring attachment representations in older children (Fairchild, 2006), which may be useful for clinicians when assessing if attachment‐based interventions increase the attachment security of foster and adopted children.

Very few studies reported on the long‐term effects of attachment‐based parenting interventions on any outcomes. Findings suggest that attachment interventions increase positive parenting behaviour at follow‐up points 3–6 months after the intervention, and this may have implications for practice, as there is a need for clinicians to continuously evaluate the sustainability of treatment results.

### 7.2 Implications for research

The evidence regarding the effects of attachment‐based parenting interventions as presented in the present review is highly encouraging from a research perspective. However, the inconclusive evidence regarding the effect of attachment‐based interventions at increasing the attachment security in foster and adopted children constitutes a serious flaw in the current evidence base. The fact that so few studies measured child attachment as an outcome was surprising given the nature of the interventions, and thus due to the low power, the results of the meta‐analysis on child attachment security should not be seen as evidence that there is no effect of attachment‐based interventions on child attachment security. Rather the results are inconclusive at this point, suggesting a need for more research meassuring attachment as an outcome. Similarly, only one usable study collected data on attachment disorganisation and thus we were unable to conduct a meta‐analysis using attachment disorganisation as an outcome, even though this would have been an extremely valuable contribution to the current knowledge base. Theoretically, it is possible that child attachment security and/or attachment disorganisation may be difficult to impact within the relatively short period of time that parenting interventions typically last. It is possible that if post‐intervention improvements in parenting behaviours are sustained over time, it may lead to possible improvement in child attachment security and a decrease in child disorganised attachment, and thus it would be particularly important for future research to explore attachment outcomes over a longer period of time.

Ten studies analysed the effect of attachment‐based interventions on positive parenting behaviour post intervention. There was a very large degree of heterogeneity between the studies and inconsistency in the magnitude of the effect sizes with two very large effect sizes. These two very large effect sizes might in part be considered as a result of the use of observations of parenting behaviour as part of a clinical assessment. It may be argued that these measures have a limited ecological validity as the behaviour displayed by highly motivated parents who know that they are being carefully observed, and who have just completed an intervention consisting in part of direct guidance on parent/child interactional behaviours, may not be representative of what their parenting is like in everyday life or in less ideal circumstances when they are tired or frustrated or when their child is resistant or displaying behaviour problems. Rather, the observational measures may be said to measure the ideal parenting which the parent is capable of under optimal circumstances, and given that foster and adoptive parents as a group consisting of resourceful individuals who voluntarily participated in interventions, it is in our view not surprising that when receiving the intervention they may be able to improve their parenting skills dramatically ‐ at least within a clinical assessment in which they know that they are being observed.

Furthermore, very few studies reported on the long‐term effects of attachment‐based parenting interventions on any outcomes, but findings suggest that attachment interventions increase positive parenting behaviour at follow‐up points 3–6 months after the intervention. Within the present review it was not possible to conduct a meta‐analysis using placement stability or breakdown as an outcome. This emphasises the need for future longitudinal research in the prevention of placement breakdown.

Finally, results of the single factor subgroup analyses suggest that the effects of interventions may differ depending on whether interventions take place in the family home or in another location. However, within the present review, we were unable to determine which location is more beneficial as firstly results were opposite for parent and child outcomes, and secondly we cannot be sure that any of the differences were not caused by other factors than location. Therefore it would be valuable for future trials to test the effects of location using the same intervention and target population.

## CONTRIBUTIONS OF AUTHORS

*Content*:

Nina T. Dalgaard is a psychologist, PhD, Nina has clinical experience with parent/child attachment interventions and has published research focusing on attachment and children's psychosocial adjustment in refugee families as well as systematic reviews focusing on family violence and trauma communication in traumatised refugee families.

Maiken Pontoppidan, PhD (public health), MA (Psychology and History), is experienced in conducting RCTs and systematic reviews of evaluating the effects of parenting interventions for parents with young children. Maiken has published two systematic reviews on parenting interventions and one has recently been submitted for publication. Maiken is currently PI of two interventions projects examining the effects of mentalization based interventions for vulnerable pregnant women and is in the trial steering group of two intervention projects aimed at vulnerable families with infants.

*Systematic review methods*:

Trine Filges, PhD (economics), is an experienced systematic reviewer and methodologist, having completed a number of systematic reviews in social welfare topic areas as well as in the field of education. Trine has published thirteen Campbell Systematic reviews, is currently the lead reviewer on three Campbell Systematic Reviews, further involved as a reviewer in two Campbell Systematic Reviews and has published systematic and meta‐analytic reviews in high‐impact journals. Trine's fields of expertise are systematic review methods and statistical analysis; and she will contribute to the quantitative data extraction, methodological quality appraisal and meta‐analysis.

Nina T. Dalgaard and Maiken Pontoppidan (see description above).

*Statistical analysis*:

Trine Filges (see description above).

*Information retrieval*:

Bjørn C. A. Viinholt (information specialist): has 4 years of experience in developing and writing systematic reviews. As a part of undertaking systematic reviews, Bjørn has experience in developing systematic search strategies and processes of reference management. Bjørn will contribute with assisting and development of the systematic search strategy, executing the searches, and assist with reference management and grey literature searches. Bjørn will also assist with aspects relating to systematic literature searches in Campbell review methodology.

## DIFFERENCES BETWEEN PROTOCOL AND REVIEW

We did not find any studies in which attachment in foster or adopted children, was compared against a normative sample, which we stated that we might include within the protocol for the review.

## CHARACTERISTICS OF STUDIES

### Characteristics of included studies

***Baker 2015***

**Table:** 

**Methods**
**Participants**
**Interventions**
**Outcomes**
**Notes**

Risk of bias table

**Table:** 

Bias	Authors' judgement	Support for judgement
Random sequence generation (selection bias)	Unclear risk	
Allocation concealment (selection bias)	Unclear risk	
Blinding of participants and personnel (performance bias)	Unclear risk	
Blinding of outcome assessment (detection bias)	Unclear risk	
Incomplete outcome data (attrition bias)	Unclear risk	
Selective reporting (reporting bias)	Unclear risk	
Other bias	Unclear risk	

***Bammens*** 2015

**Table:** 

**Methods**
**Participants**
**Interventions**
**Outcomes**
**Notes**

Risk of bias table

**Table:** 

Bias	Authors' judgement	Support for judgement
Random sequence generation (selection bias)	Unclear risk	
Allocation concealment (selection bias)	Unclear risk	
Blinding of participants and personnel (performance bias)	Unclear risk	
Blinding of outcome assessment (detection bias)	Unclear risk	
Incomplete outcome data (attrition bias)	Unclear risk	
Selective reporting (reporting bias)	Unclear risk	
Other bias	Unclear risk	

***Barone*** 2018

**Table:** 

**Methods**
**Participants**
**Interventions**
**Outcomes**
**Notes**

Risk of bias table

**Table:** 

Bias	Authors' judgement	Support for judgement
Random sequence generation (selection bias)	Unclear risk	
Allocation concealment (selection bias)	Unclear risk	
Blinding of participants and personnel (performance bias)	Unclear risk	
Blinding of outcome assessment (detection bias)	Unclear risk	
Incomplete outcome data (attrition bias)	Unclear risk	
Selective reporting (reporting bias)	Unclear risk	
Other bias	Unclear risk	

***Barone 2019***

**Table:** 

**Methods**
**Participants**
**Interventions**
**Outcomes**
**Notes**

Risk of bias table

**Table:** 

Bias	Authors' judgement	Support for judgement
Random sequence generation (selection bias)	Unclear risk	
Allocation concealment (selection bias)	Unclear risk	
Blinding of participants and personnel (performance bias)	Unclear risk	
Blinding of outcome assessment (detection bias)	Unclear risk	
Incomplete outcome data (attrition bias)	Unclear risk	
Selective reporting (reporting bias)	Unclear risk	
Other bias	Unclear risk	

***Benjamin*** 2010

**Table:** 

**Methods**
**Participants**
**Interventions**
**Outcomes**
**Notes**

Risk of bias table

**Table:** 

Bias	Authors' judgement	Support for judgement
Random sequence generation (selection bias)	Unclear risk	
Allocation concealment (selection bias)	Unclear risk	
Blinding of participants and personnel (performance bias)	Unclear risk	
Blinding of outcome assessment (detection bias)	Unclear risk	
Incomplete outcome data (attrition bias)	Unclear risk	
Selective reporting (reporting bias)	Unclear risk	
Other bias	Unclear risk	

***Bick 2013***

**Table:** 

**Methods**
**Participants**
**Interventions**
**Outcomes**
**Notes**

Risk of bias table

**Table:** 

Bias	Authors' judgement	Support for judgement
Random sequence generation (selection bias)	Unclear risk	
Allocation concealment (selection bias)	Unclear risk	
Blinding of participants and personnel (performance bias)	Unclear risk	
Blinding of outcome assessment (detection bias)	Unclear risk	
Incomplete outcome data (attrition bias)	Unclear risk	
Selective reporting (reporting bias)	Unclear risk	
Other bias	Unclear risk	

Blair 2018

**Table:** 

**Methods**
**Participants**
**Interventions**
**Outcomes**
**Notes**

Risk of bias table

**Table:** 

Bias	Authors' judgement	Support for judgement
Random sequence generation (selection bias)	Unclear risk	
Allocation concealment (selection bias)	Unclear risk	
Blinding of participants and personnel (performance bias)	Unclear risk	
Blinding of outcome assessment (detection bias)	Unclear risk	
Incomplete outcome data (attrition bias)	Unclear risk	
Selective reporting (reporting bias)	Unclear risk	
Other bias	Unclear risk	

Bywater 2011

**Table:** 

**Methods**
**Participants**
**Interventions**
**Outcomes**
**Notes**

Risk of bias table

**Table:** 

Bias	Authors' judgement	Support for judgement
Random sequence generation (selection bias)	Unclear risk	
Allocation concealment (selection bias)	Unclear risk	
Blinding of participants and personnel (performance bias)	Unclear risk	
Blinding of outcome assessment (detection bias)	Unclear risk	
Incomplete outcome data (attrition bias)	Unclear risk	
Selective reporting (reporting bias)	Unclear risk	
Other bias	Unclear risk	

Carnes‐Holt 2010

**Table:** 

**Methods**
**Participants**
**Interventions**
**Outcomes**
**Notes**

Risk of bias table

**Table:** 

Bias	Authors' judgement	Support for judgement
Random sequence generation (selection bias)	Unclear risk	
Allocation concealment (selection bias)	Unclear risk	
Blinding of participants and personnel (performance bias)	Unclear risk	
Blinding of outcome assessment (detection bias)	Unclear risk	
Incomplete outcome data (attrition bias)	Unclear risk	
Selective reporting (reporting bias)	Unclear risk	
Other bias	Unclear risk	

Carnes‐Holt 2014

**Table:** 

**Methods**
**Participants**
**Interventions**
**Outcomes**
**Notes**

Risk of bias table

**Table:** 

Bias	Authors' judgement	Support for judgement
Random sequence generation (selection bias)	Unclear risk	
Allocation concealment (selection bias)	Unclear risk	
Blinding of participants and personnel (performance bias)	Unclear risk	
Blinding of outcome assessment (detection bias)	Unclear risk	
Incomplete outcome data (attrition bias)	Unclear risk	
Selective reporting (reporting bias)	Unclear risk	
Other bias	Unclear risk	

Conn 2018

**Table:** 

**Methods**
**Participants**
**Interventions**
**Outcomes**
**Notes**

Risk of bias table

**Table:** 

Bias	Authors' judgement	Support for judgement
Random sequence generation (selection bias)	Unclear risk	
Allocation concealment (selection bias)	Unclear risk	
Blinding of participants and personnel (performance bias)	Unclear risk	
Blinding of outcome assessment (detection bias)	Unclear risk	
Incomplete outcome data (attrition bias)	Unclear risk	
Selective reporting (reporting bias)	Unclear risk	
Other bias	Unclear risk	

Danko 2014

**Table:** 

**Methods**
**Participants**
**Interventions**
**Outcomes**
**Notes**

Risk of bias table

**Table:** 

Bias	Authors' judgement	Support for judgement
Random sequence generation (selection bias)	Unclear risk	
Allocation concealment (selection bias)	Unclear risk	
Blinding of participants and personnel (performance bias)	Unclear risk	
Blinding of outcome assessment (detection bias)	Unclear risk	
Incomplete outcome data (attrition bias)	Unclear risk	
Selective reporting (reporting bias)	Unclear risk	
Other bias	Unclear risk	

Dozier 2006

**Table:** 

**Methods**
**Participants**
**Interventions**
**Outcomes**
**Notes**

Risk of bias table

**Table:** 

Bias	Authors' judgement	Support for judgement
Random sequence generation (selection bias)	Unclear risk	
Allocation concealment (selection bias)	Unclear risk	
Blinding of participants and personnel (performance bias)	Unclear risk	
Blinding of outcome assessment (detection bias)	Unclear risk	
Incomplete outcome data (attrition bias)	Unclear risk	
Selective reporting (reporting bias)	Unclear risk	
Other bias	Unclear risk	

***Dozier 2008***

**Table:** 

**Methods**
**Participants**
**Interventions**
**Outcomes**
**Notes**

Risk of bias table

**Table:** 

Bias	Authors' judgement	Support for judgement
Random sequence generation (selection bias)	Unclear risk	
Allocation concealment (selection bias)	Unclear risk	
Blinding of participants and personnel (performance bias)	Unclear risk	
Blinding of outcome assessment (detection bias)	Unclear risk	
Incomplete outcome data (attrition bias)	Unclear risk	
Selective reporting (reporting bias)	Unclear risk	
Other bias	Unclear risk	

***Dozier 2009***

**Table:** 

**Methods**
**Participants**
**Interventions**
**Outcomes**
**Notes**

Risk of bias table

**Table:** 

Bias	Authors' judgement	Support for judgement
Random sequence generation (selection bias)	Unclear risk	
Allocation concealment (selection bias)	Unclear risk	
Blinding of participants and personnel (performance bias)	Unclear risk	
Blinding of outcome assessment (detection bias)	Unclear risk	
Incomplete outcome data (attrition bias)	Unclear risk	
Selective reporting (reporting bias)	Unclear risk	
Other bias	Unclear risk	

***Guerney*** *1977*

**Table:** 

**Methods**
**Participants**
**Interventions**
**Outcomes**
**Notes**

Risk of bias table

**Table:** 

Bias	Authors' judgement	Support for judgement
Random sequence generation (selection bias)	Unclear risk	
Allocation concealment (selection bias)	Unclear risk	
Blinding of participants and personnel (performance bias)	Unclear risk	
Blinding of outcome assessment (detection bias)	Unclear risk	
Incomplete outcome data (attrition bias)	Unclear risk	
Selective reporting (reporting bias)	Unclear risk	
Other bias	Unclear risk	

***Juffer*** 1997

**Table:** 

**Methods**
**Participants**
**Interventions**
**Outcomes**
**Notes**

Risk of bias table

**Table:** 

Bias	Authors' judgement	Support for judgement
Random sequence generation (selection bias)	Unclear risk	
Allocation concealment (selection bias)	Unclear risk	
Blinding of participants and personnel (performance bias)	Unclear risk	
Blinding of outcome assessment (detection bias)	Unclear risk	
Incomplete outcome data (attrition bias)	Unclear risk	
Selective reporting (reporting bias)	Unclear risk	
Other bias	Unclear risk	

***Juffer*** 1997 ***with biological children***

**Table:** 

**Methods**
**Participants**
**Interventions**
**Outcomes**
**Notes**

Risk of bias table

**Table:** 

Bias	Authors' judgement	Support for judgement
Random sequence generation (selection bias)	Unclear risk	
Allocation concealment (selection bias)	Unclear risk	
Blinding of participants and personnel (performance bias)	Unclear risk	
Blinding of outcome assessment (detection bias)	Unclear risk	
Incomplete outcome data (attrition bias)	Unclear risk	
Selective reporting (reporting bias)	Unclear risk	
Other bias	Unclear risk	

***Juffer 2005***

**Table:** 

**Methods**
**Participants**
**Interventions**
**Outcomes**
**Notes**

Risk of bias table

**Table:** 

Bias	Authors' judgement	Support for judgement
Random sequence generation (selection bias)	Unclear risk	
Allocation concealment (selection bias)	Unclear risk	
Blinding of participants and personnel (performance bias)	Unclear risk	
Blinding of outcome assessment (detection bias)	Unclear risk	
Incomplete outcome data (attrition bias)	Unclear risk	
Selective reporting (reporting bias)	Unclear risk	
Other bias	Unclear risk	

***Juffer 2012***

**Table:** 

**Methods**
**Participants**
**Interventions**
**Outcomes**
**Notes**

Risk of bias table

**Table:** 

Bias	Authors' judgement	Support for judgement
Random sequence generation (selection bias)	Unclear risk	
Allocation concealment (selection bias)	Unclear risk	
Blinding of participants and personnel (performance bias)	Unclear risk	
Blinding of outcome assessment (detection bias)	Unclear risk	
Incomplete outcome data (attrition bias)	Unclear risk	
Selective reporting (reporting bias)	Unclear risk	
Other bias	Unclear risk	

***Lewis‐Morrarty 2012***

**Table:** 

**Methods**
**Participants**
**Interventions**
**Outcomes**
**Notes**

Risk of bias table

**Table:** 

Bias	Authors' judgement	Support for judgement
Random sequence generation (selection bias)	Unclear risk	
Allocation concealment (selection bias)	Unclear risk	
Blinding of participants and personnel (performance bias)	Unclear risk	
Blinding of outcome assessment (detection bias)	Unclear risk	
Incomplete outcome data (attrition bias)	Unclear risk	
Selective reporting (reporting bias)	Unclear risk	
Other bias	Unclear risk	

***Lind 2017***

**Table:** 

**Methods**
**Participants**
**Interventions**
**Outcomes**
**Notes**

Risk of bias table

**Table:** 

Bias	Authors' judgement	Support for judgement
Random sequence generation (selection bias)	Unclear risk	
Allocation concealment (selection bias)	Unclear risk	
Blinding of participants and personnel (performance bias)	Unclear risk	
Blinding of outcome assessment (detection bias)	Unclear risk	
Incomplete outcome data (attrition bias)	Unclear risk	
Selective reporting (reporting bias)	Unclear risk	
Other bias	Unclear risk	

***Lind 2020***

**Table:** 

**Methods**
**Participants**
**Interventions**
**Outcomes**
**Notes**

Risk of bias table

**Table:** 

Bias	Authors' judgement	Support for judgement
Random sequence generation (selection bias)	Unclear risk	
Allocation concealment (selection bias)	Unclear risk	
Blinding of participants and personnel (performance bias)	Unclear risk	
Blinding of outcome assessment (detection bias)	Unclear risk	
Incomplete outcome data (attrition bias)	Unclear risk	
Selective reporting (reporting bias)	Unclear risk	
Other bias	Unclear risk	

Martin 2013

**Table:** 

**Methods**
**Participants**
**Interventions**
**Outcomes**
**Notes**

Risk of bias table

**Table:** 

Bias	Authors' judgement	Support for judgement
Random sequence generation (selection bias)	Unclear risk	
Allocation concealment (selection bias)	Unclear risk	
Blinding of participants and personnel (performance bias)	Unclear risk	
Blinding of outcome assessment (detection bias)	Unclear risk	
Incomplete outcome data (attrition bias)	Unclear risk	
Selective reporting (reporting bias)	Unclear risk	
Other bias	Unclear risk	

***McCullough*** 2019

**Table:** 

**Methods**
**Participants**
**Interventions**
**Outcomes**
**Notes**

Risk of bias table

**Table:** 

Bias	Authors' judgement	Support for judgement
Random sequence generation (selection bias)	Unclear risk	
Allocation concealment (selection bias)	Unclear risk	
Blinding of participants and personnel (performance bias)	Unclear risk	
Blinding of outcome assessment (detection bias)	Unclear risk	
Incomplete outcome data (attrition bias)	Unclear risk	
Selective reporting (reporting bias)	Unclear risk	
Other bias	Unclear risk	

***Mersky 2015***

**Table:** 

**Methods**
**Participants**
**Interventions**
**Outcomes**
**Notes**

Risk of bias table

**Table:** 

Bias	Authors' judgement	Support for judgement
Random sequence generation (selection bias)	Unclear risk	
Allocation concealment (selection bias)	Unclear risk	
Blinding of participants and personnel (performance bias)	Unclear risk	
Blinding of outcome assessment (detection bias)	Unclear risk	
Incomplete outcome data (attrition bias)	Unclear risk	
Selective reporting (reporting bias)	Unclear risk	
Other bias	Unclear risk	

***Mersky 2016***

**Table:** 

**Methods**
**Participants**
**Interventions**
**Outcomes**
**Notes**

Risk of bias table

**Table:** 

Bias	Authors' judgement	Support for judgement
Random sequence generation (selection bias)	Unclear risk	
Allocation concealment (selection bias)	Unclear risk	
Blinding of participants and personnel (performance bias)	Unclear risk	
Blinding of outcome assessment (detection bias)	Unclear risk	
Incomplete outcome data (attrition bias)	Unclear risk	
Selective reporting (reporting bias)	Unclear risk	
Other bias	Unclear risk	

***Midgley 2019***

**Table:** 

**Methods**
**Participants**
**Interventions**
**Outcomes**
**Notes**

Risk of bias table

**Table:** 

Bias	Authors' judgement	Support for judgement
Random sequence generation (selection bias)	Unclear risk	
Allocation concealment (selection bias)	Unclear risk	
Blinding of participants and personnel (performance bias)	Unclear risk	
Blinding of outcome assessment (detection bias)	Unclear risk	
Incomplete outcome data (attrition bias)	Unclear risk	
Selective reporting (reporting bias)	Unclear risk	
Other bias	Unclear risk	

Nilsen 2007

**Table:** 

**Methods**
**Participants**
**Interventions**
**Outcomes**
**Notes**

Risk of bias table

**Table:** 

Bias	Authors' judgement	Support for judgement
Random sequence generation (selection bias)	Unclear risk	
Allocation concealment (selection bias)	Unclear risk	
Blinding of participants and personnel (performance bias)	Unclear risk	
Blinding of outcome assessment (detection bias)	Unclear risk	
Incomplete outcome data (attrition bias)	Unclear risk	
Selective reporting (reporting bias)	Unclear risk	
Other bias	Unclear risk	

***N´zi 2016***

**Table:** 

**Methods**
**Participants**
**Interventions**
**Outcomes**
**Notes**

Risk of bias table

**Table:** 

Bias	Authors' judgement	Support for judgement
Random sequence generation (selection bias)	Unclear risk	
Allocation concealment (selection bias)	Unclear risk	
Blinding of participants and personnel (performance bias)	Unclear risk	
Blinding of outcome assessment (detection bias)	Unclear risk	
Incomplete outcome data (attrition bias)	Unclear risk	
Selective reporting (reporting bias)	Unclear risk	
Other bias	Unclear risk	

***Opiola 2016***

**Table:** 

**Methods**
**Participants**
**Interventions**
**Outcomes**
**Notes**

Risk of bias table

**Table:** 

Bias	Authors' judgement	Support for judgement
Random sequence generation (selection bias)	Unclear risk	
Allocation concealment (selection bias)	Unclear risk	
Blinding of participants and personnel (performance bias)	Unclear risk	
Blinding of outcome assessment (detection bias)	Unclear risk	
Incomplete outcome data (attrition bias)	Unclear risk	
Selective reporting (reporting bias)	Unclear risk	
Other bias	Unclear risk	

***Opiola 2018***

**Table:** 

**Methods**
**Participants**
**Interventions**
**Outcomes**
**Notes**

Risk of bias table

**Table:** 

Bias	Authors' judgement	Support for judgement
Random sequence generation (selection bias)	Unclear risk	
Allocation concealment (selection bias)	Unclear risk	
Blinding of participants and personnel (performance bias)	Unclear risk	
Blinding of outcome assessment (detection bias)	Unclear risk	
Incomplete outcome data (attrition bias)	Unclear risk	
Selective reporting (reporting bias)	Unclear risk	
Other bias	Unclear risk	

***Purvis 2015***

**Table:** 

**Methods**
**Participants**
**Interventions**
**Outcomes**
**Notes**

Risk of bias table

**Table:** 

Bias	Authors' judgement	Support for judgement
Random sequence generation (selection bias)	Unclear risk	
Allocation concealment (selection bias)	Unclear risk	
Blinding of participants and personnel (performance bias)	Unclear risk	
Blinding of outcome assessment (detection bias)	Unclear risk	
Incomplete outcome data (attrition bias)	Unclear risk	
Selective reporting (reporting bias)	Unclear risk	
Other bias	Unclear risk	

***Razuri 2016***

**Table:** 

**Methods**
**Participants**
**Interventions**
**Outcomes**
**Notes**

Risk of bias table

**Table:** 

Bias	Authors' judgement	Support for judgement
Random sequence generation (selection bias)	Unclear risk	
Allocation concealment (selection bias)	Unclear risk	
Blinding of participants and personnel (performance bias)	Unclear risk	
Blinding of outcome assessment (detection bias)	Unclear risk	
Incomplete outcome data (attrition bias)	Unclear risk	
Selective reporting (reporting bias)	Unclear risk	
Other bias	Unclear risk	

***Rushton*** 2010

**Table:** 

**Methods**
**Participants**
**Interventions**
**Outcomes**
**Notes**

Risk of bias table

**Table:** 

Bias	Authors' judgement	Support for judgement
Random sequence generation (selection bias)	Unclear risk	
Allocation concealment (selection bias)	Unclear risk	
Blinding of participants and personnel (performance bias)	Unclear risk	
Blinding of outcome assessment (detection bias)	Unclear risk	
Incomplete outcome data (attrition bias)	Unclear risk	
Selective reporting (reporting bias)	Unclear risk	
Other bias	Unclear risk	

***Schoemaker*** 2020

**Table:** 

**Methods**
**Participants**
**Interventions**
**Outcomes**
**Notes**

Risk of bias table

**Table:** 

Bias	Authors' judgement	Support for judgement
Random sequence generation (selection bias)	Unclear risk	
Allocation concealment (selection bias)	Unclear risk	
Blinding of participants and personnel (performance bias)	Unclear risk	
Blinding of outcome assessment (detection bias)	Unclear risk	
Incomplete outcome data (attrition bias)	Unclear risk	
Selective reporting (reporting bias)	Unclear risk	
Other bias	Unclear risk	

***Selwyn*** 2009

**Table:** 

**Methods**
**Participants**
**Interventions**
**Outcomes**
**Notes**

Risk of bias table

**Table:** 

Bias	Authors' judgement	Support for judgement
Random sequence generation (selection bias)	Unclear risk	
Allocation concealment (selection bias)	Unclear risk	
Blinding of participants and personnel (performance bias)	Unclear risk	
Blinding of outcome assessment (detection bias)	Unclear risk	
Incomplete outcome data (attrition bias)	Unclear risk	
Selective reporting (reporting bias)	Unclear risk	
Other bias	Unclear risk	

Sprang 2009

**Table:** 

**Methods**
**Participants**
**Interventions**
**Outcomes**
**Notes**

Risk of bias table

**Table:** 

Bias	Authors' judgement	Support for judgement
Random sequence generation (selection bias)	Unclear risk	
Allocation concealment (selection bias)	Unclear risk	
Blinding of participants and personnel (performance bias)	Unclear risk	
Blinding of outcome assessment (detection bias)	Unclear risk	
Incomplete outcome data (attrition bias)	Unclear risk	
Selective reporting (reporting bias)	Unclear risk	
Other bias	Unclear risk	

***Stams 2001***

**Table:** 

**Methods**
**Participants**
**Interventions**
**Outcomes**
**Notes**

Risk of bias table

**Table:** 

Bias	Authors' judgement	Support for judgement
Random sequence generation (selection bias)	Unclear risk	
Allocation concealment (selection bias)	Unclear risk	
Blinding of participants and personnel (performance bias)	Unclear risk	
Blinding of outcome assessment (detection bias)	Unclear risk	
Incomplete outcome data (attrition bias)	Unclear risk	
Selective reporting (reporting bias)	Unclear risk	
Other bias	Unclear risk	

Stevens, 2011

**Table:** 

**Methods**
**Participants**
**Interventions**
**Outcomes**
**Notes**

Risk of bias table

**Table:** 

Bias	Authors' judgement	Support for judgement
Random sequence generation (selection bias)	Unclear risk	
Allocation concealment (selection bias)	Unclear risk	
Blinding of participants and personnel (performance bias)	Unclear risk	
Blinding of outcome assessment (detection bias)	Unclear risk	
Incomplete outcome data (attrition bias)	Unclear risk	
Selective reporting (reporting bias)	Unclear risk	
Other bias	Unclear risk	

***Van Andel 2016***

**Table:** 

**Methods**
**Participants**
**Interventions**
**Outcomes**
**Notes**

Risk of bias table

**Table:** 

Bias	Authors' judgement	Support for judgement
Random sequence generation (selection bias)	Unclear risk	
Allocation concealment (selection bias)	Unclear risk	
Blinding of participants and personnel (performance bias)	Unclear risk	
Blinding of outcome assessment (detection bias)	Unclear risk	
Incomplete outcome data (attrition bias)	Unclear risk	
Selective reporting (reporting bias)	Unclear risk	
Other bias	Unclear risk	

***Van Holen 2017***

**Table:** 

**Methods**
**Participants**
**Interventions**
**Outcomes**
**Notes**

Risk of bias table

**Table:** 

Bias	Authors' judgement	Support for judgement
Random sequence generation (selection bias)	Unclear risk	
Allocation concealment (selection bias)	Unclear risk	
Blinding of participants and personnel (performance bias)	Unclear risk	
Blinding of outcome assessment (detection bias)	Unclear risk	
Incomplete outcome data (attrition bias)	Unclear risk	
Selective reporting (reporting bias)	Unclear risk	
Other bias	Unclear risk	

Wassall, 2011

**Table:** 

**Methods**
**Participants**
**Interventions**
**Outcomes**
**Notes**

Risk of bias table

**Table:** 

Bias	Authors' judgement	Support for judgement
Random sequence generation (selection bias)	Unclear risk	
Allocation concealment (selection bias)	Unclear risk	
Blinding of participants and personnel (performance bias)	Unclear risk	
Blinding of outcome assessment (detection bias)	Unclear risk	
Incomplete outcome data (attrition bias)	Unclear risk	
Selective reporting (reporting bias)	Unclear risk	
Other bias	Unclear risk	

***Yarger 2020***

**Table:** 

**Methods**
**Participants**
**Interventions**
**Outcomes**
**Notes**

Risk of bias table

**Table:** 

Bias	Authors' judgement	Support for judgement
Random sequence generation (selection bias)	Unclear risk	
Allocation concealment (selection bias)	Unclear risk	
Blinding of participants and personnel (performance bias)	Unclear risk	
Blinding of outcome assessment (detection bias)	Unclear risk	
Incomplete outcome data (attrition bias)	Unclear risk	
Selective reporting (reporting bias)	Unclear risk	
Other bias	Unclear risk	

### Characteristics of excluded studies

**Table:** 

***Fisher*** 2008	
**Reason for exclusion**	Outcomes are not relevant for the present review
***Linares*** 2006	
**Reason for exclusion**	Biological and foster parents participated as pairs and the overall goal of treatment was family reunification
Minnis 1999	
**Reason for exclusion**	Intervention was only psychoeducation, psychoeducation lasted 3 days (4 h each day), and only one day focused on attachment
***Minnis*** 2001	
**Reason for exclusion**	Intervention was only psychoeducation, psychoeducation lasted 3 days (4 h each day), and only one day focused on attachment
*Myeroff* 1998	
**Reason for exclusion**	Intervention deemed an unvalidated treatment
***Schoemaker*** 2020	
**Reason for exclusion**	Only pre‐test data is reported
***Wilson*** 2006	
**Reason for exclusion**	Intervention is not based on attachment theory

## DATA AND ANALYSES

### Child outcomes

**Table:** 

Outcome or Subgroup Studies	Participants	Statistical Method	Effect Estimate
1.1 Child overall psychosocial adjustment	10	Std. Mean Difference (IV, Random, 95% CI)	0.37 [0.10, 0.65]
1.2 Externalizing behavior	8	Std. Mean Difference (IV, Random, 95% CI)	0.37 [−0.09, 0.83]
1.3 Internalizing symptoms	5	Std. Mean Difference (IV, Random, 95% CI)	0.20 [−0.02, 0.42]
1.4 Observed attachment security	3	Std. Mean Difference (IV, Random, 95% CI)	0.59 [−0.40, 1.57]
1.5 Observed positive child behavior	4	Std. Mean Difference (IV, Random, 95% CI)	0.39 [0.14, 0.64]

### Parent outcomes

**Table:** 

Outcome or Subgroup Studies	Participants	Statistical Method	Effect Estimate
2.1 Observed positive parent behavior	10	Std. Mean Difference (IV, Random, 95% CI)	1.56 [0.81, 2.31]
2.2 Parenting stress	9	Std. Mean Difference (IV, Random, 95% CI)	0.24 [0.03, 0.46]
2.3 Parental depressive symptoms	3	Std. Mean Difference (IV, Random, 95% CI)	0.59 [−0.08, 1.25]

### Follow Up

**Table:** 

Outcome or Subgroup Studies	Participants	Statistical Method	Effect Estimate
3.1 Child Externalising Behavior	3	Std. Mean Difference (IV, Random, 95% CI)	0.04 [−0.78, 0.87]
3.2 Child Internalising Behavior	2	Std. Mean Difference (IV, Random, 95% CI)	0.32 [−0.68, 1.32]
3.3 Observed Positive Parenting	3	Std. Mean Difference (IV, Random, 95% CI)	0.54 [0.03, 1.06]
3.4 Parenting Stress	2	Std. Mean Difference (IV, Random, 95% CI)	0.60 [−0.15, 1.35]

### Subgroup Child outcomes

**Table:** 

Outcome or Subgroup Studies	Participants	Statistical Method	Effect Estimate
4.6 Adopted Subgroup Child overall psychosocial adjustment	10	Std. Mean Difference (IV, Random, 95% CI)	Subtotals only
4.6.1 Foster	5	Std. Mean Difference (IV, Random, 95% CI)	0.39 [−0.11, 0.88]
4.6.2 Adopted	5	Std. Mean Difference (IV, Random, 95% CI)	0.35 [0.01, 0.70]
4.7 Age Subgroup Child overall psychosocial adjustment	10	Std. Mean Difference (IV, Random, 95% CI)	Subtotals only
4.7.1 3 years and older	8	Std. Mean Difference (IV, Random, 95% CI)	0.29 [−0.01, 0.59]
4.7.2 Less than 3 years	2	Std. Mean Difference (IV, Random, 95% CI)	0.78 [0.04, 1.52]
4.8 Location Subgroup Child overall psychosocial adjustment	10	Std. Mean Difference (IV, Random, 95% CI)	Subtotals only
4.8.1 Therapy in the family home	4	Std. Mean Difference (IV, Random, 95% CI)	0.81 [0.47, 1.15]
4.8.2 Therapy in other location	6	Std. Mean Difference (IV, Random, 95% CI)	0.11 [−0.09, 0.31]
4.9 Adopted Subgroup Externalizing behavior	8	Std. Mean Difference (IV, Random, 95% CI)	Subtotals only
4.9.1 Foster children	5	Std. Mean Difference (IV, Random, 95% CI)	0.73 [0.02, 1.43]
4.9.2 Adopted children	3	Std. Mean Difference (IV, Random, 95% CI)	−0.07 [−0.38, 0.24]
4.10 Location Subgroup Externalizing behavior	8	Std. Mean Difference (IV, Random, 95% CI)	Subtotals only
4.10.1 Therapy in family home	2	Std. Mean Difference (IV, Random, 95% CI)	0.68 [−1.34, 2.71]
4.10.2 Therapy in other location	6	Std. Mean Difference (IV, Random, 95% CI)	0.19 [−0.06, 0.44]

### Subgroup Parent outcomes

**Table:** 

Outcome or Subgroup Studies	Participants	Statistical Method	Effect Estimate
5.1 Adopted Subgroup Observed positive parent behavior	10	Std. Mean Difference (IV, Random, 95% CI)	Subtotals only
5.1.1 Foster	5	Std. Mean Difference (IV, Random, 95% CI)	2.57 [0.84, 4.30]
5.1.2 Adopted	5	Std. Mean Difference (IV, Random, 95% CI)	1.01 [0.56, 1.46]
5.4 Age Subgroup Observed positive parent behavior	10	Std. Mean Difference (IV, Random, 95% CI)	Subtotals only
5.4.1 3 years and older	7	Std. Mean Difference (IV, Random, 95% CI)	2.19 [0.99, 3.38]
5.4.2 Less than 3 years	3	Std. Mean Difference (IV, Random, 95% CI)	0.56 [0.06, 1.06]
5.5 Location Subgroup Observed positive parent behavior	10	Std. Mean Difference (IV, Random, 95% CI)	Subtotals only
5.5.1 Therapy in the family home	5	Std. Mean Difference (IV, Random, 95% CI)	0.58 [0.26, 0.90]
5.5.2 Therapy in other location	5	Std. Mean Difference (IV, Random, 95% CI)	3.29 [1.13, 5.45]
5.6 Adopted Subgroup Parenting stress	9	Std. Mean Difference (IV, Random, 95% CI)	Subtotals only
5.6.1 Foster	7	Std. Mean Difference (IV, Random, 95% CI)	0.20 [−0.04, 0.43]
5.6.2 Adopted	2	Std. Mean Difference (IV, Random, 95% CI)	0.59 [−0.01, 1.19]
5.7 Age Subgroup Parenting stress	9	Std. Mean Difference (IV, Random, 95% CI)	Subtotals only
5.7.1 3 years and older	7	Std. Mean Difference (IV, Random, 95% CI)	0.34 [0.08, 0.60]
5.7.2 Less than 3 years	2	Std. Mean Difference (IV, Random, 95% CI)	0.04 [−0.33, 0.40]
5.8 Location Subgroup Parenting stress	9	Std. Mean Difference (IV, Random, 95% CI)	Subtotals only
5.8.1 Therapy in the family home	4	Std. Mean Difference (IV, Random, 95% CI)	0.07 [−0.21, 0.36]
5.8.2 Therapy in other location	5	Std. Mean Difference (IV, Random, 95% CI)	0.45 [0.11, 0.80]

## Supporting information

Supporting information.

## Footnotes

^1^

http://www.europarl.europa.eu/RegData/etudes/BRIE/2016/583860/EPRS_BRI(2016)583860_EN.pdf.

^2^
Members of the review team at VIVE Campbell were: Research Analyst Morten Kjær Thomsen (MKT) the research assistants Julie Haatuft (JH) Tanne Ebert Jørgensen (TEJ), Anton Dam (ANDA) Kristine Grosen Ellerman (KGE).
