# Correction to “Parenting Interventions to Support Parent/Child Attachment and Psychosocial Adjustment in Foster and Adoptive Parents and Children: A Systematic Review”

**DOI:** 10.1002/cl2.70064

**Published:** 2025-08-20

Dalgaard, N. T., T. Filges, B. C. A. Viinholt, and M. Pontoppidan. 2021. “Parenting Interventions to Support Parent/Child Attachment and Psychosocial Adjustment in Foster and Adoptive Parents and Children: A Systematic Review.” *Campbell Systematic Reviews* 18, no. 1: e1209. https://doi.org/10.1002/cl2.1209.

The “Characteristics of Included Studies” section and tables were erroneously included. Content under the “CHARACTERISTICS OF STUDIES” up until the “Characteristics of Excluded Studies” section starts has been removed.

We apologize for this error.

