# Supplementary material for: Parenting interventions to support parent/child attachment and psychosocial adjustment in foster and adoptive parents and children: A systematic review
Source: Campbell Syst Rev. 2022 Jan 5;18(1):e1209. doi: 10.1002/cl2.1209 (PMC8732982; doi:10.1002/cl2.1209)
Supplement: Supplementary file 1 — Supporting information. [file CL2-18-e1209-s001.docx]

# Appendices

## 1 Descriptive Table

[Enter text here]

|  |  |  |  |  | **Placement** | | | **Child characteristics** | | | | **F/A parent characteristics** | **Therapist characteristics** | | **Intervention characteristics** | | | | | |  |
| --- | --- | --- | --- | --- | --- | --- | --- | --- | --- | --- | --- | --- | --- | --- | --- | --- | --- | --- | --- | --- | --- |
| **Author(s)** | **Title** | **Journal** | **Year** | **Country** | **Foster children %** | **Adopted %** | **Domestic adopted % (of the adopted)** | **Child age at the adoption/placement, average/min/max** | **Child age at the intervention, average/min/max** | **Child gender % girls** | **Child country of origin** | **F/A parent ethnicity** | **Therapist education** | **Therapist years of experience** | **Only one parent and child in each family participates %** | **Setting (in the families’ homes, hospital, outpatient clinic, community facility)** | **Duration of intervention (number of weeks/months from first to last session)** | **Number of sessions** | **Intervention based mainly/only partly on attachment theory** | **Name of intervention** | **Comparison (no treatment/TAU/other intervention/waitlist)** |
| **VIPP intervention** | |  |  |  |  |  |  |  |  |  |  |  |  |  |  |  |  |  |  |  |  |
| Femmie Juffer, René A. C. Hoksbergen, J. Marianne Riksen-Walraven & Geldolph A. Kohnstamm | Early Intervention in Adoptive Families: Supporting Maternal Sensitive Responsiveness, Infant-Mother Attachment, and Infants Competence | The Journal of Child Psychology and Psychiatry | 1997 | Holland | 0 | 100 | Only internationally adopted children | "To participate, the adopted child had to be the first child in the family and placed before 5 months of age." The children were placed for adoption at a mean age of 8 weeks (SD=3,67, range=2-18 weeks). | The study took place while the adopted infant was aged between 5 and 12 months | 46 girls and 44 boys (girls=51,1%) | 90 families with an interracially adopted infant (71 from Sri Lanka and 19 from Korea) | "The current study is restricted to Asian children placed as infants into white families." | The intervention was provided by three female researchers with a master's degree in child and family studies | Not reported | Mother-infant dyads | In the families' homes | Not reported | The book+video group consisted of the personal book (?) combined with three sessions of video feedback, implemented during home visits ---- The book group only consisted of the personal book (?) | Based on attachment theory | Two intervention groups: Book group and Book+video group | Control group (no intervention) |
| F. Juffer, L.G. Rosenboom, R.A.C. Hoksbergen, J.M.A. Riksen-Walraven & G.A. Kohnstamm | Attachment and intervention in adoptive families with and without biological children | Book chapter | 1997 | Holland | 0 | 100 | Only internationally adopted children | "The infants in study 2, adoptive families with biological children, were placed at an older age (mean age: 14 weeks) than the infants in study 1, adoptive families without biological children (mean age: 8 weeks)..." | Not reported | 51 boys and 49 girls (girls=49%) | The children were adopted from Sri Lanka (53), South Korea (34) & Colombia (13) | White | Not reported | Not reported | Mother-infant dyads | In the families' homes | Not reported | The parents in the intervention group received a Dutch booklet titled 'The First Year of Life' and the parents received three sessions with video feedback | Based on attachment theory | Intervention: Book+video intervention group | Control group (no intervention) |
| Stams, Gert-Jan J. M. et. al | Attachment-based intervention in adoptive families in infancy and children's development age 7: Two follow-up studies | British Journal of Developmental Psychology | 2001 | Holland | 0 | 100 | Internationally, transracially adopted children | Placed before 6 months of age | 7 years | Study 1: 54,28 %; study 2: 51,79 % | Study 1: Shri lanka, South Korea and Columbia; Study 2: Shri Lanka and South Korea | Study 1 and 2: caucasien white. | Not reported | Not reported | Focus on the mother of the families | Family home visits + visits at the laboratory | Home visits when the child is 5, 6, 9, 12 months and 7 years + | Unclear | Based mainly on attachment theory | Two early attachment-based intervention programmes implemented in infancy | Study 1: mixed families (small sample), study 2: all adoptive families (larger sample) |
| Femmie Juffer, Marian J. Bakermans-Kranenburg, & Marinus H. van Ijzendoorn | The importance of parenting in the development of disorganized attachment: evidence from a preventive intervention study in adoptive families | The Journal of Child Psychology and Psychiatry | 2005 | Holland | 0 | 100 | Only internationally adopted children | "All the children came into their adoptive home before the age of 6 months (M=10 weeks, SD=4,93, range 2-23 weeks)." | "Families were visited at home when the adopted infant was 5, 6, 9, and 12 months old. Mother and child came to the laboratory at 12 and 18 months. The interventions were carried out between 6 and 9 months." | 64 girls and 66 boys (girls=49,2%) | 130 families with children adopted from Sri Lanka (78), South Korea (39) and Colombia (13) | "The parents were white …" | "In the first subsample, the interventions were implemented by three female interveners (the first author, being one of them, trained the other two). In the second subsample, two (other) female interveners (trained by the first author) carried out the intervention." | Not reported | Mother-child dyads | In the families' homes | Not reported | Not reported | Two attachment-based intervention programs | Two intervention groups: Book-only intervention group and Book+video feedback intervention group | Control group (no intervention) |
| Femmie Juffer, Marinus H. van Ijzendoorn & Marian J. Bakermans-Kranenburg | Supporting adoptive families with video-feedback intervention | (Book): Promoting Positive Parenting – An Attachment-Based Intervention | 2008 | Holland | 0 | 100 | Only internationally adopted children | "All the children came into their adoptive home before the age of 6 months (M=10 weeks, SD=4,93, range 2-23 weeks)." | "Families were visited at home when the adopted infant was 5, 6, 9, and 12 months old. Mother and child came to the laboratory at 12 and 18 months. The interventions were carried out between 6 and 9 months." | 64 girls and 66 boys (girls=49,2%) | 130 families with children adopted from Sri Lanka (78), South Korea (39) and Colombia (13) | "The parents were white …" | "The interventions were implemented by five female home visitors …" | Not reported | Mother-child dyads | In the families' homes | "The video-feedback group (…) was provided with three sessions of video-feedback (…) This intervention was implemented in two home visits at six months and one at nine months, and each intervention session lasted approximately one hour." | Not reported | Two attachment-based intervention programs | Two intervention groups: Book-only intervention group and Book+video feedback intervention group | "In both samples, the control group received a booklet on adoption issues as a "dummy" intervention." |
| **VIPP intervention** | |  |  |  |  |  |  |  |  |  |  |  |  |  |  |  |  |  |  |  |  |
| Lavinia Barone, Virginia Barone, Antonio Dellagiulia, Francesca Lionetti | Testing an Attachment-Based Parenting Intervention-VIPP-FC/A in Adoptive Families with Post-institutionalized Children: Do Maternal Sensitivity and Genetic Markers | Frontiers in Psychology | 2018 | Italien | 0 | 100 | International adoption | Age at adoption was recorded but not reported | 33.18 months SD= 16.83 months | 46.75% girls | Not reported | Caucasian (95 % Italian) | Educational background was not reported but all interveners were trained and certified for adherence in VIPP | Not reported | Only adoptive mothers and children participated | In the family home | 6-7 weeks | 7 (1, assessment + 6 intervention sessions) | Based  mainly on attachment theory | Video-feedback Intervention to Promote Positive Parenting and Sensitive Discipline VIPP-FC/A | Control condition consisted of 6 telephone calls in which general issues of child development were discussed |
| Lavinia Barone, Yagmur Ozturk & Francesca Lionetti | The key role of positive parenting and children's temperament in post-institutionalized children's socio-emotional adjustment after adoption placement. A RCT study | Social Development | 2019 |  |  | 100 | Internationally adopted children | Child's age at adoption (month): Mean age=33,5, SD=17,1 | Child's age at assessment (month): Mean age=43,3, SD=15,9 | Girls=42% | Asia=29%, Europe=42%, America=12%, Africa=17% | Not reported | Not reported | Not reported | Mother-child dyads | The VIPP-FC/A is a home-visiting intervention | Not reported | "The protocol involves seven home visits: an initial session to collect a baseline video of parent-child interaction and six intervention sessions." | Based  mainly on attachment theory | The Video-feedback Intervention to promote Positive Parenting in Adoption and Foster Care (VIPP-FC/A) | Control condition: A dummy intervention (a home visit to collect a baseline video of parent-child interaction followed by six telephone calls from an intervener at every planned week). |
| **VIPP intervention** | |  |  |  |  |  |  |  |  |  |  |  |  |  |  |  |  |  |  |  |  |
| Nikita K. Schoemaker, Femmie Juffer, Ralph C.A. Rippe, Harriet J. Vermeer, Marije Stoltenborgh, Gabrine J. Jagersma, Athanasios Maras & Lenneke R.A. Alink | Positive parenting in foster care: Testing the effectiveness of a videofeedback intervention program on foster parents’ behavior and attitudes | Children and Youth Services Review, Elsevier | 2020 | Holland | 100 | 0 | Not reported | Not reported | VIPP-FC: mean age=3,6 years, SD=1,45 | BOYS %: VIPP-FC=46,7% | Not reported | Not reported | Not reported | Not reported | Only one foster parent-child dyad per family could participate | Home visits | 3-4 months | 6 intervention home visits (a biweekly interval between the first four home visits and an interval of about three to four weeks between the last two home visits) | The intervention is based on attachment theory and coercion theory | Video-feedback Intervention to promote Positive Parenting and Sensitive Discipline in Foster Care (VIPP-FC) | Control condition (The foster parents in the control group received a dummy intervention that consisted of six telephone calls) |
| **Online Emotional Attachment and Emotional Availability (EA2) Intervention** | | | | | | |  |  |  |  |  |  |  |  |  |  |  |  |  |  |  |
| Baker, Megan et. al | Emotional Attachment and Emotional Availability tele-intervention for adoptive families | Infant Mental Health Journal vol. 36 | 2015 | The United States | 0 | 100 | 47 % = domestically; 13 % = internationally; 40 % = fostercare system | Children were typically 0-3 moths when adopted, although one third were adopted at 4-52 months of age | 1,5-5 years. On average 42 months old at pretest | 40 % girls | Not reported | Most parents idenitified as Caucasian and a small number dientified as multiracial | The first author: experience working with adoptive families and trained in the EA system. The second author: A licensed clinical and developmental psychologist supervised the sessions. | Several years | Both adoptive dyads (one parent and one adoptive child) + some spouses participated in the individual sessions | In the family home through skype and video | 6 weeks | Unclear | based mainly on attachment theory | In vivo EA2 Intervention: Emotional Attachment and Emotional Availability Tele-intervention | The EA2 tele-intervention had a large effect on both groups: an immediate-intervention group and a delayed-intervention group |
| **The Family Minds (FM) psycho-educational and interactive programme** | | | | | |  |  |  |  |  |  |  |  |  |  |  |  |  |  |  |  |
| Anne-Sophie Bammens, Tina Adkins & Julia Badger | Psycho-educational intervention increases reflective functioning in foster and adoptive parents | Adoption & Fostering | 2015 | UK (study undertaken in Texas) | Not reported | Not reported | Not reported | Not reported | In the intervention group, the average age of the foster/adoptive child was 5 years 10 months … In the comparison group, the average age of the foster/adoptive child was 5 years 5 months. | Not reported | Not reported | Not reported | "The same person, who has a clinical background and training in mentalisation, taught all classes in both training conditions." | Not reported | Not reported | Not reported | "The intervention group underwent a total of nine training hours designed in three parts, each three hours in length, delivered over three sessions and spread out over several weeks to allow the parents an opportunity to practice their new skills at home and discuss with the group afterwards." | Not reported | Not reported | The Family Minds (FM) programme | "… a comparison group who experienced a 'treatment as usual' intervention comprising four hours of lecture information about trauma and attachment." |
| **Biopsychosocial-based versus behavioral-based parenting model** | | | | |  |  |  |  |  |  |  |  |  |  |  |  |  |  |  |  |  |
| Janet L. Benjamin | Biopsychosocial-Based Versus Behavioral-Based Parenting Model: A Clinical Trial for Adoptive Parents With Attachment-Challenged Children | Dissertation Abstracts International: Section B: The Sciences and Engineering | 2010 | USA | 0 | 100 | Not reported | Not reported | 6-15 years. BIPM (mean=8,15, SD=3,02, range=5-15), LLP (mean=8,20, SD=2,72, range=5-14), WLC (mean=11,50, SD=4,06, range=6-16) | BIPM: 80% female, LLP: 70% female, WLC: 55% female | Not reported | See table 1 page 54… | BIPM: Conducted by the primary researcher, who is certified as an instructor in the stress model of parenting. LLP: Conducted by a research assistant, who was certified as an instructor of this model of parenting. | Not reported | Not reported | Not reported | BIPM & LLP = 7-week programs. The duration, number of sessions, therapist education, etc. of the interventions in the two treatment groups are described in detail on page 67-69. | Not reported | Not reported | Two interventions:  The Love and Logic Parenting (LLP) & The Benjamin interactive parenting model (BIPM) | Nontreatment wait list control (WLC) group |
| **ACB Intervention (Dozier foster care)** | | |  |  |  |  |  |  |  |  |  |  |  |  |  |  |  |  |  |  |  |
| Teresa Lind, K. Lee Raby, E. B. Caron, Caroline K. P. Roben & Mary Dozier | Enhancing executive functioning among toddlers in foster care with an attachment-based intervention |  | 2017 | USA | 100 (the low risk comparison group consists of children raised by their birth parents) | Not reported | Not reported | ABC-T: Age first removed from birth parents (months): Mean=12,5, SD=13,3. Time with caregiver at postassessment (months): Mean=28,3, SD=14,2. | DEF: mean=31,8 months (SD=8,7), min=14,5 max=54,7. ABC-T: mean=29,9 months (SD=9,5), min=14,0 max=56,3. Ages also reported at follow-up (postintervention) see Table 2 | DEF = 51,7%, ABC-T = 42,9%, Low risk comparison = 51,9% | **Children were**: **DEF** (22,4% White, 58,6% African American, 1,7% Asian American, 5,2% Hispanic, 12,1% Biracial); **ABC-T** (28,6% White, 54% African American, 7,9% Hispanic, 9,5% Biracial); Low risk comparison (51,9% White, 21,2% African American, 5,8% Asian American, 11,5% Hispanic, 9,6% Biracial) | **Parents were**: **DEF** (37,5% white, 57,9% African American, 4,2% Hispanic, 10,4% Biracial); **ABC-T** (51% white, 45,1% African American, 3,9% Hispanic); Low-risk comparison group (53,8% white, 25% African American, 9,6% Asian American, 11,5% Hispanic) ... | Not reported | Not reported | Only parent-toddler dyads | Both the DEF and the ABC interventions were conducted in families' homes |  | Both interventions (DEF and ABC-T) consisted of 10 sessions |  | Intervention: The Attachment and Biobehavioral Catch-up for Toddlers (ABC-T) intervention ... Control intervention: Developmental Education for Families (DEF) | Control intervention: DEF. Low risk comparison group: Children who had never been placed in foster care and were raised by their birth parents |
| Mary Dozier, Elizabeth Peloso, Oliver Lindhiem, M. Kathleen Gordon, Melissa Manni, Sandra Sepulveda, & John Ackerman | Developing Evidence-Based Interventions for Foster Children: An Example of a Randomized Clinical Trial with Infants and Toddlers | Journal of Social Issues | 2006 | USA | 100 | Not reported | Not reported | Not reported | Children from the foster care sample ranged in age at the time of the postintervention assessments from 3.6 to 39.4 months … If looking only at the experimental group: Min:3,9 Max: 39,4 (mean=19,01) | 50% | Children were: African American (63%), White (32%) & Biracial (5%) | Not reported | Parent trainers were professional socialworkers or psychologists | They had at least 5 years clinical experience | Not reported | Sessions took place in the foster families' homes | 10 weeks (10 weekly sessions) | 10 sessions | Not reported | Experimental intervention: Attachment and Biobehavioral catch-up intervention (ABC) … Control intervention: Developmental education for families (DEF) | Control intervention: Developmental education for families (DEF) |
| Dozier, Mary et. Al | Effects of an attachment-based intervention on the cortisol production of infants and toddlers in foster care | National Institutes of Health | 2008 | The United States | 100 | Not reported | Not reported | Not reported | 15-24 months | ABC=59%, DEF=43%, Comparison group=44% | Not reported | Not reported | Parent trainers had bachelor's or master's degrees in psychology or social work | A minimum of 5 years of clinical experience |  | Three laboratories affiliated with the University of Delaware | 10 weeks | 10 sessions | Based mainly on attachment theory | Attachment and Biobehavioral Catch-up (ABC) + control intervention = Developmental Education for families (DEF) | ABC: 46 children; DEF: 47 children; Comparison group:48 children |
| Mary Dozier, Oliver Lindhiem, Erin Lewis, Johanna Bick, Kristin Bernard, Elizabeth Peloso | Effects of Foster Parent Training Program on Young Children's Attachment Behaviors: Preliminary Evidence from a Randomized Trial | Child Adolesc Soc Work J | 2009 | USA | 100 | Not reported | Not reported | Not reported | Mean age in months= 18.9 (SE= 1.8) min= 3.9, max = 39.4 | it says half of the children were boys and half of the children were girls | USA | Children were: 63 % African Amarican, 26% non-Hispanic White, 3% Hispanic  7 % biracial, only 8 children were placed with foster parents of different ethnicity p. 327 | Professional social workers and psychologists | A minimum of 5 years of clincal experience | Yes | In the family home | 10 weeks | 10 sessions | Based  mainly on attachment theory | Attachment and Biobehavioral Catch-Up Intervention (ABC) | Control intervention consisting of 10 weekly sessions focusing on child cognitive development and language |
| Johanna Bick & Mary Dozier | The effectiveness of an attachment-based intervention in promoting foster mothers' sensitivity toward foster infants | Infant Mental Health Journal | 2013 | USA | 100 | Not reported | Not reported | Not reported | At the start of the intervention: Infants ranged from 1 to 22 months of age (M=9,9, SD=6,05) - Table 1 has some different numbers… | Girls: 48% | Not reported | Foster mothers were 43% African American, 46% White non-Hispanic, 7% Hispanic, & 4% biracial | Parent trainers | "Parent trainers who had extensive experience working with parents and infants delivered intervention sessions for both programs." | Parent (foster mother)-infant dyads | In the foster mothers' homes | Not reported | 10 sessions | Based  mainly on attachment theory | Attachment and Biobehavioral Catch-up Intervention | The Developmental Education for Families intervention (control intervention) |
| Lewis-Morrarty, Erin et al. | Cognitive Flexibility and Theory of Mind Outcomes Among Foster Children: Preschool Follow-Up Results of a Randomized Clinical Trial | Journal of Adolescent Health | 2012 | USA | 100 (many different characteristics (p. 19-20) | Not reported | Not reported | 7,5 months | 61 children: 4-6 years (mean = 60,3 months) | 49,2% | Unclear | Only female parents. 57,4%=European American, 39,3 % = african american, 3,3%=Asian American. | Not reported | Not reported | Not reported | A home visit and a laboratory visit | Not reported | 10 sessions | Based mainly on attachment theory | Attachment and Biobehavioral Catch-up (ABC) | Comparison with foster care control group and non foster control group |
| **ABC Intervention (Dozier adoption)** | | |  |  |  |  |  |  |  |  |  |  |  |  |  |  |  |  |  |  |  |
| Teresa Lind, K. Lee Raby, Alison Goldstein, Kristin Bernard, EB Caron, Heather A. Yarger, Allison Wallin And Mary Dozier | Improving social-emotional competence in internationally adopted children with Attachment and Biobehavioral Catch-up intervention | Development and psychopathology | 2020 | USA | Not reported | 100 | Only internationally adopted children | 16.5 months (SD = 6.9) | ABC: mean= 21.6 months (SD = 7.1); DEF: 20.1 months (SD = 6.3) | ABC: 52.3 %; DEF: 50 % | 38.9 % chinese, 18.3 % russian, 17.6 % south korrean, 11.5 % Etheopian and 13.7 % other. (This is from the overall group; intervention specific ethnicity is avialable, but that isn't country of birth oriented). | ABC: White = 98.4 %, Asian American = 1.6 %; DEF: White = 93.7 %, African American = 1.6 %, Asian American = 4.8 % | postbaccalaureate, graduates or postdocs with 1.5 hour weekly training sessions and reviewing the protocol to become parent coaches. | At least one year of trial period under supervision of trained Parent coach. | Not reported | In familie's homes. | Not reported | 10 sessions | Based partly on attachment theory | Attachment and Biobehavioral Catch-Up (ABC) intervention | Control intervention: Developmental Education for Families (DEF) |
| Heather A. Yarger, Kristin Bernard, EB Caron, Allison Wallin & Mary Dozier | Enhancing Parenting Quality for Young Children Adopted Internationally: Results of a Randomized Controlled Trial | Journal of Clinical Child & Adolescent Psychology | 2019 | USA | Not reported | 100 | Only internationally adopted children | Age at adoption (in months): Mean=16,3, range: 4,9 to 37,8 months, SD=6,9 | Table 1: ABC intervention (mean=22,2 months, SD=8,5 months, range: 8,0-47,2 months), DEF intervention (mean=22,4 months, SD=9,7 months, range: 9,1-50,5 months). The abstract: "… children ranged in age from 6,8 months to 48,4 months (M=21,9, SD=9,0)." | Female=52,5% (ABC intervention=50,8%, DEF intervention=54,1%) | Not reported | White/Non-Hispanic=95% | Parent coaches ? | Not reported | Not reported | Delivered in the families' homes | Not reported | 10 weekly sessions | Not reported | Attachment and Biobehavioral Catch-Up (ABC) intervention | Control intervention: Developmental Education for Families (DEF) |
| **ABC-T intervention** | |  |  |  |  |  |  |  |  |  |  |  |  |  |  |  |  |  |  |  |  |
| Ginny Sprang | The Efficacy of a Relational Treatment for Maltreated Children and their Families | Child and Adolescent Mental Health | 2009 | USA | 100 | 0 | Not reported | Not reported | 0-6 years (mean age in months=42,5 (ca. 3,5 years), SD=18,6 months | 26 female and 27 male children | Not reported | 53 caregivers/adult study participants (47 were white, 6 were African American) | There were four therapists (one child psychiatrist, one psychiatric nurse practitioner, and two licensed clinical social workers) | The therapists had a minimum of five years experience providing mental health services to young children and had over 85 years of clinical experience combined | Parent-child dyads | In the caregivers' homes | Not reported | Not reported | Not reported | The Attachment and Biobehavioral Catchup Intervention (ABC) | Control condition |
| **Incredible Years** | |  |  |  |  |  |  |  |  |  |  |  |  |  |  |  |  |  |  |  |  |
| Bywather, T. et. al | Incredible Years parent training support for foster carers in Wales: a multi-centre feasibility study | Child: care, health and development (original article?) | 2010 | Wales | 100 | 0 | Not reported | Not reported | 2-17 years | 47,82% | Not reported | Not reported | Seven facilitators (p. 235) | Some experienced and some non-experienced | Foster carers who were looking after more than one child were asked to focus on one child for this study | Not reported | 12 weeks | 12 sessions | The study does not focus on attachment theory | The IY basic parenting programme (Webster-Stratton 1989) | Intervention group improvements compared to the control group |
| **Incredible Years** | |  |  |  |  |  |  |  |  |  |  |  |  |  |  |  |  |  |  |  |  |
| Conn, Anne-Marie et. al | Pilot randomized controlled trial of foster parent training: a mixed-methods evaluation of parent and child outcomes | Children and Youth Services Review | 2018 | The United States | 100 | 0 | Not reported | Table 1, p. 193 | 2-7 years | Table 1, p. 193 | Table 1, p. 193 | Table 1, p. 193 | Master-level psychologist + experienced foster parents as mentors | Master-level psychologist | Not reported | A off-site community based location and the pediatric medical home | 13 weeks | Not reported | The study does not focus on attachment theory | Incredible Years: IY: trauma-informed, evidence-based parenting program | Mixed methods. Intervention group: N=19 fosterfamilies; Control group: N=19 |
| **Incredible Years** | |  |  |  |  |  |  |  |  |  |  |  |  |  |  |  |  |  |  |  |  |
| Wendy Nilsen | Fostering Futures: A Preventive Intervention Program for School-age Children in Foster Care | Clinical Child Psychology and Psychiatry | 2007 | USA | 100 | 0 | Not reported | Not reported | Children aged 5-12 years (average age=8,11 years, SD=1,61) | 61,1% girls (treatment group=63,6%, comparison group=57,1%) | Treatment group, children: 63,6% African American, 36,4% White, 0% Hispanic | Treatment group, foster caregivers: 63,6% African American, 36,4% White, 0% Hispanic | "Foster caregiver mentor/trainers included four foster caregivers selected by a consensus panel of five local foster care experts." (p. 51) | "Mentor trainers were all female foster caregivers with between 4 and 27 years of experience in the child welfare system." (p. 51) | "When a foster family had two caregivers (n = 5) both were encouraged to participate, although one family consistently attended sessions with both caregivers." (p. 50) | Not reported | 12-week group foster caregiver-training program | Not reported | No mentioning of attachment theory - but mentioning of social learning theory | Fostering Futures Program? "This pilot study augmented an existing evidenced-based intervention (i.e. the Incredible Years) developed for birth families for use with foster caregivers." (p. 45) | Comparison group |
| **PCIT** | |  |  |  |  |  |  |  |  |  |  |  |  |  |  |  |  |  |  |  |  |
| Katelyn Blair | Parent-Child Interaction Therapy and Resilience Within Child Welfare | Dissertation, University of Wisconsin - Milwaukee | 2018 | USA | 100 | 0 | Not reported | Not reported | Average=4,6 years of age (SD=1,31) | Female=56,9% | Not reported | Foster parent participants were 48,4% white, 45,9% African-American and 5,7% were coded as 'Other' | Not reported | Not reported | Foster parent-child dyads | Not reported | Caregiver-child dyads were randomized into one of three conditions: 1) A brief condition (two days of training and eigth weeks of phone calls), 2) An extended condition (two days of training and fourteen weeks of phone calls, plus a third day of training as a booster session, and 3) A control condition (services-as-usual). | Not reported | Parent-Child Interaction Therapy (PCIT) draws on attachment and social learning theories | Parent-Child Interaction Therapy (PCIT). | Foster parent-child dyads receiving child welfare services-as-usual |
| Christina Marie Danko | The Effect of Parent-Child Interaction Therapy on Strengthening the Attachment Relationship with Foster Parents and Children in Foster Care | College of Science and Health Theses and Dissertations | 2014 | USA | 100 | 0 | Not reported | "… the foster child must have been placed with the foster parents for at least two months." | 2-5 years (mean age=3,56 years, SD=0,99, range=2,08–5,67) | Male=74,1% | Child ethnicity was 66,7% African American, 11,1% Latino, 3,7% Caucasian, 3,7% Asian & 14,8% Multiracial. | 66,7% African American, 14,8% Latino & 18,5% Caucasian. | "Home visits were conducted by observers who received training and supervision from the principal investigator." "Therapy sessions were conducted by the principal investigator and two additional doctoral students who had at least one year of training in PCIT." | Not reported | Not reported | In the families' homes | "Families assigned to the CDI-only treatment condition participated in 10-14 total 60-minutes CDI sessions of PCIT up to twice a week. Families assgined to the CDI plus PDI treatment condition participated in 5-7 CDI and 5-7 PDI sessions twice a week for a total of 10-14 sessions of 60 minutes." |  | Based on attachment and social learning theory | Parent-Child Interaction Therapy (PCIT) – CDI-only or CDI plus PDI treatment. | Waitlist bibliotherapy condition |
| **PCIT brief + extended** | |  |  |  |  |  |  |  |  |  |  |  |  |  |  |  |  |  |  |  |  |
| Joshua P. Mersky, James Topitzes, Colleen E. Janczewski & Cheryl B. McNeil | Enhancing Foster Care Parent Training with Parent-Child Interaction Therapy: Evidence from a Randomized Field Experiment | Journal of the Society for Social Work & Research | 2015 | USA | 100 | 0 | Not reported | Not reported | The total sample of children averaged 4,6 years of age at enrollment (range: 2/2,5-7 years) | Of the total sample of children, 56% were girls | Not reported | 49% of the foster parents were non-Hispanic Caucasian, 45% were African American, 5% were Hispanic | Not reported | Not reported | Foster-parent-child dyads | Foster parents and foster children assigned to one of the treatment conditions attended PCIT trainings together with six to eight families at a large, urban child welfare agency (they were compensated for transportation costs and completing the PCIT trainings). The foster parents also received periodic telephone consultation at home. | "PCIT is usually provided over 12 to 20 weekly sessions by a therapist (…)" (p. 593) … "The extended PCIT condition received 3 days of group training and 14 weeks of home-based intervention." (p. 601) … As I understand, the brief PCIT condition received 2 days of group training and 8 weeks of home-based intervention... | Not reported | No mentioning of attachment theory | Parent-child interaction therapy, two treatment groups: Brief PCIT & Extended PCIT. | Waitlist control |
| Joshua P. Mersky, James Topitzes, Stacey D. Grant-Savela, Michael J. Brondino & Cheryl B. McNeil | Adapting Parent-Child Interaction Therapy to Foster Care: Outcomes From a Randomized Trial | Research on Social Work Practice | 2016 | USA | 100 | 0 | Not reported | Not reported | Sample children averaged 4,6 years of age (children were qualified if they were between 3 and 6 years old) | 54% were female of the sample of children | Not reported | 51% were racial and ethnic minorities | Not reported | Not reported | Parent-child dyads | "Foster parents who were assigned to treatment attended PCIT trainings at a child welfare agency with their foster children and other foster parent-child dyads." | Brief PCIT group comprised of two full-fay trainings plus 8 weeks of telephone consulation and homework, whereas the extended PCIT group comprised of three full-day trainings and 14 weeks of telephone consultation and homework | Not reported | Not reported | Parent-child interaction therapy, two treatment groups: Brief PCIT & Extended PCIT. | Waitlist control |
| **Trust-Based Relational Intervention** | | |  |  |  |  |  |  |  |  |  |  |  |  |  |  |  |  |  |  |  |
| Karyn B. Purvis, Erin Becker Razuri, Amanda R. Hiles Howard, Casey D. Call, Jamie Hurst DeLuna, Jordan S. Hall & David R. Cross | Decrease in Behavioral Problems and Trauma Symptoms Among At-Risk Adopted Children Following Trauma-Informed Parent Training Intervention | Journal of Child & Adolescent Trauma | 2015 | USA | 0 | 100 | Adoption type, Domestic: Treatment (37,5%), Control (37,5%) | Age at adoption (in months): Treatment (mean=33,69), Control (mean=37,70). | Eligible children were between 5-12 years at the beginning of the study. Current age in years: Treatment (mean=7,88), Control (mean=7,88) | Treatment (female=37,5%), Control (female=37,5%) | Not reported | Treatment (Hispanic/Latino=2,1%, White/Caucasian=97,9%), Control (Hispanic/Latino=0%, White/Caucasian=100%) | Not reported | "Trainers each had approximately 2 years experience using the standardized presentations, manuals, and workbooks." | Not reported | Not reported | "Participants in the treatment group attended a 4-day TBRI parent training (6 h per day) …" | Not reported | "TBRI is a trauma-informed intervention grounded in attachment theory …" | Trust-Based Relational Intervention (TBRI) | Control group: Offered online training after the conclusion of the study. |
| Erin Becker Razuri; Amanda R. Hiles Howard; Sheri R. Parris; Casey D. Call; Jamie Hurst DeLuna; Jordan S. Hall; Karyn B. Purvis; David R. Cross. | Decrease in Behavioral Problems and Trauma Symptoms Among At-Risk Adopted Children Following Web-Based Trauma-Informed Parent Training Intervention | Journal of Evidence-Informed Social Work | 2016 | USA | 0 | 100 | Not reported, but see child ethnicity | Treatment group mean: 43.96 months (SD = 32.91); control group mean: 41.78 months (SD = 31.44) | Treatment group mean: 8.18 years (SD = 2.13); control group mean: 8.12 years (SD = 2.08) | 50 % for both treatment and control group | Only ethnicity reported: Treatment group: 18 % asian, 28.9 % black/african american, 8.6 % hispanic/latino, 40,6 % white/caucasian, 0.8 % native american, 3.1 % other; control group: 16.4 % asian, 26.6 % black/african american, 14.8 % hispanic/latino, 38.3 % white/caucasian, 1.6 % native american, 2.3 % other. | Treatment group: 0.8 % asian, 0.8 % black/african american, 0.8 % hispanic/latino, 95,3 % white/caucasian, 0.0 % native american, 2.3 % other; control group: 0.8 % asian, 0.0 % black/african american, 0.8 % hispanic/latino, 97.7 % white/caucasian, 0.8 % native american, 0.0 % other. |  |  | No | Online | 30 days | 18 | "TBRI is a trauma-informed intervention grounded in attachment theory …" | Trust-Based Relational Intervention (TBRI) | Control group |
| **Child Directed Interaction Training** | | |  |  |  |  |  |  |  |  |  |  |  |  |  |  |  |  |  |  |  |
| Monica Leah Stevens | Child Directed Interaction Training: The Impact on the Kinship Caregiver-Child Relationship and Child Externalizing and Internalizing Symptoms | Dissertation | 2011 | USA | 100 | 0 | Not reported | Not reported | 5 years and 4 months | 63% | Only ethnicity reported for families as an entirety. 64 % caucasian, 18 % african american, 9 % hispanic and 9 % biracial. | See Child country of origin | Graduate students who recieved training in PCIT (parent-child interaction thearapy) | 3-credit-hour course (with supervision on at least first two PCIT cases prior to training study participants). | Not reported | Unclear | 4 weeks | 8 | Partly | No name | Wait-list control |
| Amanda M. N'zi, Monica L. Stevens & Sheila M. Eyberg | Child Directed Interaction Training for Young Children in Kinship Care: A Pilot Study | Child Abuse & Neglect | 2016 |  | 100 | 0 | Not reported | Not reported | 2-7 years (mean=5,2, range=2,0-7,5) | 50% female | Not reported | Not reported | Not reported | Not reported | Not reported | "Training was delivered at a local, community library …" | 4 weeks | Twice weekly, 8 sessions | Not reported | Child Directed Interaction Training (CDIT) | Waitlist control condition |
| **Child Parent Relationship Therapy (CPRT)** | | |  |  |  |  |  |  |  |  |  |  |  |  |  |  |  |  |  |  |  |
| Opiola, Kristie K. | The effects of Child Parent Relationship Theory (CPRT)for Adoptive Families | Dissertation Prepared for the Degree of Doctor of Philosophy | 2016 | The United States | 0 | 100 | 84%=adopted internationally or from foster care system | 16%<1 year; 37% 12-23 months; 14 % 2-4 years; 33 % over the age of 5 years | 2,5-9 years, mean age:5,5 | 50% |  | 85 % European American, 6% Latino, 6% Asian, 2% Black | Five advanced level doctoral counseling students + one counseling faculty member with advanced training and clinical experience in CCPT, CRPT and adoption issues + one supervisor with advanced training | Not reported | Not reported | Convenient sites throughout a large metropolitan area | 10 weeks | 10 or 11 sessions (unclear). Childcare, snacks and age appropriate activities was provided | Based only partly on attachment theory. Familial stress is related to attachment and behavioral issues | Child parent relationship therapy (CRPT): a replication of Carnes-Holt and Bratton's 2014 research | 49 parents: 25: experimental group. 24: control group |
| Kristie K. Opiola & Sue C. Bratton | The Efficacy of Child Parent Relationship Therapy for Adoptive Families: A Replication Study | Journal of Counseling & Development | 2018 | USA | 0 | 100 | See Figure 1 | Age at adoption (in years): CPRT (<1 month (n=2), 1-6 months (n=0), 7-11 months (n=1), 12-23 months (n=11), 2-4 (n=2), 5-6 (n=4), 7+ (n=5)… Se figure 1 for age at adoption for TAU group… | 2,5-9 years. CPRT group (mean=5,70 years), TAU group (mean=5,23 years) | CPRT group: female (n=16), male (n=9), TAU group: female (n=8), male (n=16) | Not reported | CPRT: Caucasian (n=24), African-American (n=1), Hispanic/Latino (n=0), Asian (n=0) … Ethnicity for TAU group see figure 1… | "CPRT facilitators were five advanced doctoral counseling students and one counseling faculty member (second author) with advanced training and clinical experience in CCPT, CPRT, and adoption and attachment issues." | Not reported | "The experimental (CPRT) group was composed of 25 parents, of whom 20 participated as couples and five as individuals." | "Group sessions were held at convenient sites throughout a large metropolitan area." | Experimental condition (CPRT): "… parents participated in CPRT once per week for 2 hours for 10 weeks and in a 2-hour pretreatment session …" | Not reported | Not reported | Child parent relationship therapy (CPRT) | Control group: Treatment as usual (TAU) |
| **Child Parent Relationship Therapy (CPRT)** | | |  |  |  |  |  |  |  |  |  |  |  |  |  |  |  |  |  |  |  |
| Kara Carnes-Holt | Child-parent relationship therapy (CPRT) with adoptive families: Effects on child behavior, parent-child relationship stress, and parental empathy | Dissertation Abstracts International: Section B: The Sciences and Engineering | 2010 | USA | 0 | 100 | Experimental group (32 children): Child adopted by means of "Domestic/Agency"=4 children | Experimental group (32 children): <1 month=2, 1-6 months=3, 7 months-1 year=3, 1-3 years=10, 4-5 years=7, 5-7 years=5, 8+ years=2 | Current age of child (?) in the experimental group (32 children): 2-4 years=11, 5-7 years=13, 8-10 years=8 (mean age=5,8) | Not reported | Not reported | 54 European American, 3 Black American, 3 Hispanic/Latino & 1 who chose not to indicate ethnicity (total for the experimental and control group) | Not reported | Not reported | "The study included 23 couples and 15 individual mothers." | Not reported | From the Informed Consent Form: "The entire project should take approximately twenty-nine hours. There will be twenty-four hours of training plus time to complete paperwork for pre and post-tests. You will meet weekly with the trainer for two hours in a group with other parents. You will also do seven weekly play sessions with your child at home. Each play session will be about thirty minutes." | 10 sessions | Not reported | Intervention: Child-parent relationship therapy (CPRT) | Wait list control group |
| Carnes-Holt, Kara; Bratton, Sue C. | The Efficacy of Child Parent Relationship Therapy for Adopted Children With Attachement Disruptions | Journal of Counseling & Development Vol. 92 | 2014 | The United States | 0 | 100 | 47 % = European Americans; 42 % = international | 14 children: younger than 1 year of age, 38 children: 1-5 years of age, 9 children: over the age of 5 years. | 2-10 years. Mean age: 5.8 for the experimental group and 5.6 for the control group. | Not reported | Not reported | 88,5 % of parents reported European American. | A licensed professional counselor-supervisor and registered play therapist-supervisor with extensice training in play therapy and CPRT protocol | Not reported | Parents participating with a spouse (n=23 pairs) + individuals particapating without a spouse (n=15) | Adoption agency facilities, churches, community clinic + recorded  video play sessions in the family homes | 10 weeks | 10 sessions | Based mainly on attachment theory: a parent's ability to communicate empathy is essential for developing secure attachments | CRPT: A randomized control group design to examine the effets of child parent relationship therapy: group sessions with other parents + play sessions with parent and child | The experimental group and the controlgroup (waitlist) was compared after 10 weeks : the experimental group showed statistical signifcant improvements looking at empathy and the childrens behavior problems |
| **Skills Training Program for Foster Parents** | | |  |  |  |  |  |  |  |  |  |  |  |  |  |  |  |  |  |  |  |
| Louise Guerney | A Description and Evaluation of a Skills Training Program for Foster Parents | American Journal of Community Psychology | 1977 | USA | 100 | 0 | Not reported | Not reported | 5-12 years | Not reported | Not reported | "The profile of the typical foster parent involved at all points in this study was as follows: (…) white (with about 25% black) …" | "Group leaders were 12 graduate students from the Pennsylvania State University specializing in interpersonal relationships training." | Not reported | Not reported | "All groups met in agency offices except one which met at a nearby suburban campus of The Pensylvania State University." | 10 weeks (= 20-hour program) | "Meeting were held once a week for 10 weeks." | Not reported | Foster-Parent Training Program (no mentioning of a specific name…) | Control group (no treatment) |
| **3-5-7 Model** | |  |  |  |  |  |  |  |  |  |  |  |  |  |  |  |  |  |  |  |  |
| Felicia Martin | Comparing the 3-5-7 model, cognitive behavioral therapy, and treatment as usual for children and youth in long term foster care |  | 2013 | USA | 100 | 0 | Not reported | "The participants of this study consisted of foster children that have been in placement for at least five years …" | 8-16 years (mean=12) | 3-5-7 model-intervention = 50%. CBT = 50%. Treatment as usual = 48%. | Not reported | Not reported | "The therapists used in this study were employed by Canyon Acres Children and Family Services and were all post master’s degree clinicians. All therapists providing therapy were trained in the same manner." | Not reported | Not reported | Not reported | 6 months | Maximum 24 sessions | Not reported | Intervention: The 3-5-7 model; Cognitive behavioral therapy (CBT); Treatment as usual | Treatment as usual (and CBT?) |
| **Neuro-Physiological Psychotherapy (NPP)** | | |  |  |  |  |  |  |  |  |  |  |  |  |  |  |  |  |  |  |  |
| Elaine McCullough; Amy Mathura | A comparsion between a Neuro-Physiological Psychotherapy (NPP) treatment group and a control group for children adopted from care: Support for a neurodevelopmentally informed approach to therapeutic intervention with maltreated children | Child Abuse & Neglect | 2019 | UK | 0 | 100 | Not reported | Not reported but for intervention group: Child age at intervention (M = 9.47 years) - years child had lived with adoptive family prior to assessment (M = 5.54 years) = 3.93 years; for control group:   Child age at intervention (M = 9.92 years) - years child had lived with adoptive family prior to assessment (M = 6.71 years) = 3.21 years | Intervention group: M = 9.47 years ( SD = 2.74); control group: M = 9.92 years (SD = 3.69 years) | Intervention group: 53%; Control group: 32 % | Only ethnicity reported. Intervention group: 60 % white british, 10 % white other, 3 % central asian, 3 % asian, 7 % mixed white and black african, 3 % central america, 7 % mixed white and black caribbean, and 7 % mixed white and south american; control group: 68 % white white british, 27 % white other,  5 % central asian | Not reported | Therapy was provided by teams typically made up of one therapist per child and one per parent/s. Child and adult therapists  typically were Psychotherapists, Social Workers or Clinical Psychologists with master’s or doctoral level training. Parents were also provided support by adoptive parent mentors. Training and supervision were provided to ensure model fidelity | Not reported | 100 | Not reported | M = 5.54 years (SD = 2.09) | 47 | Partly | Neuro-Physiological Psychotherapy (NPP) | The control group consisted of adoptive families who were evaluated using the protocol assessment tools and recommended the full model but did not receive the intervention due to the family or the local funding authority choosing alternatives which were not comparable to NPP. |
| **MBT-foster care** | |  |  |  |  |  |  |  |  |  |  |  |  |  |  |  |  |  |  |  |  |
| Nick Midgley, Sarah Jane Besser, Pasco Fearon, Solange Wyatt, Sarah Byford & David Wellsted | The Herts and Minds study: feasibility of a randomised controlled trial of Mentalization-Based Treatment versus usual care to support the wellbeing of children in foster care. | BMC Psychiatry | 2019 | UK | 100 | 0 | Not reported | First in care (both groups (UCC&MBT): mean age=4,8, SD=3,3) … Usual care (mean age=5,2, SD=3,3), MBT (mean age=4,4, SD=3,3). | 5-16 years (both groups (MBT&UCC): mean age=10,6 years, SD=2,7) … Usual care (mean age=10,2, SD=3,0), MBT (mean age=11,1, SD=2,2). | BOYS %: All = 56% ... Usual care = 57%, MBT = 53% | Children were: All (89% White British), Usual care (86% White British), MBT (93% White British) | Not reported | "The study was conducted in a Child and Adolescent Mental Health Services (CAMHS) Targeted team within a single NHS Trust." The Targeted CAMHS team was made up of Clinical Psychologists, Social Workers and Play Therapists. | The Targeted CAMHS team had specific expertise in working with children in care and foster carers | Not reported | Not reported | Not reported | "MBT is a short-term manualized treatment, offering up to 12 weekly sessions …" | Not reported | Mentalization-based treatment (MBT) ... (Or Usual Clinical Care (UCC)) | Usual Clinical Care (UCC) |
| **The Educational Programme** | |  |  |  |  |  |  |  |  |  |  |  |  |  |  |  |  |  |  |  |  |
| Alan Rushton, Elizabeth Monck, Morven Leese, Paul McCrone, and Jessica Sharac | Enhancing adoptive parenting: A randomized controlled trial | Clinical Child Psychology and Psychiatry | 2010 | UK | 0 | 100 | Not reported, but child ethnicity is: Intervention group: 84 % white; Control group: 88 % white. | Intervention group mean age in months: 68 (SD = 19); Control group: 65 (SD = 17). | Range: 3-8 years | Intervention group: 53 %; control group: 55 % | Not reported | Not reported | "Experienced child and family social workers familiar with adoption were enlisted to act as parent advisers. They were trained to use one of the interventions and were provided with the manual and guidance on its use. Supervision was available from one of the respective practice consultants." | Not reported | Does not seem to include children | In-home based | Within nine months | 10 | Partly | The "Educational" programme (article also includes a programme called The cognital behavioural programme) | Control group |
| **The Foster Family Intervention** | | |  |  |  |  |  |  |  |  |  |  |  |  |  |  |  |  |  |  |  |
| Hans Van Andel, Lucres Jansen, Wendy Post, Rutger Jan Van der Gaag, Erik Knorth & Hans Grietens | Optimizing Foster Family Placement for Infants and Toddlers: A Randomized Controlled Trial on the Effect of the Foster Family Intervention | American Journal of Orthopsychiatry | 2016 | Holland | 100 | 0 | Not reported | Not reported | Foster children below the age of 5. Foster Family Intervention (FFI): Mean age (months)=19,7, SD=14,4 … Care As Usual (CAU): Mean age (months)=17,9, SD=14,7 | BOYS %: FFI=49%, CAU=51% | Not reported | Not reported | Trained foster care workers | Not reported | Not reported | In the families' homes | The home visits take place once every 2 weeks, covering a period of maximum 3 months | 6 sessions | The intervention is designed with inclusion of principles from attachment theory, psycho-education, mindfulness therapy, and video reflection | Foster Carer-Foster Child Intervention / Foster Family Intervention (FFI) | The Foster Family Intervention (FFI) was compared to a control group receiving care as usual (CAU) (regular foster care support) |
| **Intervention based on The Social Learning Theory for Foster Parents** | | | | | |  |  |  |  |  |  |  |  |  |  |  |  |  |  |  |  |
| Van Holen, Frank; Vanschoonlandt, Femke; Vanderfaeillie, Johan | Evaluation of a foster parent intervention for foster children with externalizing problem behaviour |  | 2017 | Belgium | 100 (children with externalizing  problem behaviour) | 0 | Not reported | Not reported | 3-12. Average: 6,14 years old | Not reported | Not reported | Not reported | Foster care workers | Special training = six 4-hour sessions | Two-parent families: 73 %, single-parent households (n=17) | Family homes | Not reported | 10 sessions | Based on socia learning theory | Intervention for foster parents who take care of children with behavioral problems (also focus on parent stress) | No effects on breakdowns looking at the control and experimental group |
| **CAKE Adopter Training** | |  |  |  |  |  |  |  |  |  |  |  |  |  |  |  |  |  |  |  |  |
| Julie Selwyn, Sarah del Tufo & Lesley Frazer | It's a Piece of Cake? An evaluation of an adopter training programme | Adoption & Fostering | 2009 | UK | 100 | 0 | Not reported | Intervention group mean: 8.6 years; Control group mean: 7.2 years | Not reported | Not reported | Not reported | "Most, but not all, participants were white" | No therapists are involved, however, the training is delivered by "trainers", which are either adopters themself or volunteers in one of two agencies. They recieve training from Adoption UK and Evaluation Trust. | Amount of training isn't mentioned in regards to years. | No | Not reported - this article is, however, an evalution of the intervention, which is why some details are left out. Should maybe find something original about the programme. | Only described in modules; six modules | Duration of sessions not reported; see 'number of sessions'. Duration of modules were at least five hours. | Having issues finding more detailed information on the programme. Should be further explored for more detailed information. | The Cake Programme | Wait-list (consisted of families that were interested in the Cake programme, but had not done so yet). |
| **Parenting Group Programmes** | |  |  |  |  |  |  |  |  |  |  |  |  |  |  |  |  |  |  |  |  |
| Sarah Wassall | Evaluation of an Attachment Theory Based Parenting Programme for Forster Carers and Adoptive Parents | University of Birmingham Research Arcive | 2011 | UK | 40% | 60% | Not reported | Not reported | Mean = 8.31 years (SD = 4.67), range 0-15.5 years. | 50% | Not reported, but ethnicity is: 83,33 % british white, 11,11 % british asian, 5,55 % other. | 88 % british white; 8 % asian british; 4 % other. | Not reported | Not reported | Mixed | Not reported | Not reported | 18 sessions | Mainly | Fostering attachment | Control group by waitlist |

## 2 Electronic searches

*SocIndex*

*Searched through EBSCO-host. Search performed 13/11/2019.*

| **Search** | **Search Terms** | **Results** |
| --- | --- | --- |
| S18 | S6 AND S10 AND S14 AND S17 | 1,565 |
| S17 | S15 OR S16 | 364,817 |
| S16 | AB (treatmen* OR intervent* OR therap* OR program*) | 346,010 |
| S15 | TI (treatmen* OR intervent* OR therap* OR program*) | 95,071 |
| S14 | S11 OR S12 OR S13 | 711,122 |
| S13 | DE "CLINICAL trials" OR DE "RANDOMIZED controlled trials" OR DE "REGRESSION discontinuity design" | 5,309 |
| S12 | AB (effect* OR trial* OR experiment* OR control* OR random* OR impact* OR compar*) | 673,562 |
| S11 | TI (effect* OR trial* OR experiment* OR control* OR random* OR impact* OR compar*) | 154,581 |
| S10 | S7 OR S8 OR S9 | 451,079 |
| S9 | DE "ATTACHMENT behavior" OR DE "ATTACHMENT behavior in children" OR DE "PRENATAL bonding" OR DE “PSYCHOLOGY of foster children" OR DE “SOCIAL conditions of foster children” OR DE "PARENT-child relationship" OR DE "PATHOLOGICAL psychology" OR DE "AFFECTIVE disorders" OR DE "ATTACHMENT disorder" OR DE "BEHAVIOR disorders in children" OR DE "POST-traumatic stress disorder" OR DE "CHILD abuse" OR DE "BATTERED child syndrome" OR DE "CHILD sexual abuse" OR DE "PSYCHOLOGICAL child abuse" OR DE “SEPARATION (Psychology)” OR DE “ABANDONMENT (Psychology)” OR DE “TREATMENT of emotional trauma” OR DE “EMOTIONAL trauma” OR DE “ADVERSE childhood experiences” OR DE “DEVELOPMENTAL psychopathology” | 46,716 |
| S8 | AB (sensitiv* OR emoti* OR dyadic* OR attach* OR relation*) | 406,781 |
| S7 | TI (sensitiv* OR emoti* OR dyadic* OR attach* OR relation*) | 78,170 |
| S6 | S1 OR S2 OR S3 OR S4 OR S5 | 27,957 |
| S5 | DE “ADOPTION” OR DE “ADOPTIVE parents” OR DE “ADOPTEES” OR DE ”FOSTER children” OR DE “FOSTER parents” OR DE ”FOSTER mothers” OR DE “FOSTER home care” OR DE “KINSHIP care” OR DE “PERMANENCY planning” | 7,945 |
| S4 | AB out-of-home | 1,379 |
| S3 | TI out-of-home | 396 |
| S2 | AB (adopt* OR foster*) AND AB (parent* OR child* OR famil* OR home* OR care*) | 24,700 |
| S1 | TI (adopt* OR foster*) AND TI (parent* OR child* OR famil* OR home* OR care*) | 4,638 |

*Econlit*

*Searched through EBSCO-host. Search performed 13/11/2019.*

| **Search** | **Search Terms** | **Results** |
| --- | --- | --- |
| S15 | S5 AND S8 AND S11 AND S14 | 112 |
| S14 | S12 OR S13 | 97,964 |
| S13 | AB (treatmen* OR intervent* OR therap* OR program*) | 84,843 |
| S12 | TI (treatmen* OR intervent* OR therap* OR program*) | 25,245 |
| S11 | S9 OR S10 | 544,837 |
| S10 | AB (effect* OR trial* OR experiment* OR control* OR random* OR impact* OR compar*) | 473,486 |
| S9 | TI (effect* OR trial* OR experiment* OR control* OR random* OR impact* OR compar*) | 169,540 |
| S8 | S6 OR S7 | 172,237 |
| S7 | AB (sensitiv* OR emoti* OR dyadic* OR attach* OR relation*) | 156,767 |
| S6 | TI (sensitiv* OR emoti* OR dyadic* OR attach* OR relation*) | 29,400 |
| S5 | S1 OR S2 OR S3 OR S4 | 4,535 |
| S4 | AB out-of-home | 131 |
| S3 | TI out-of-home | 23 |
| S2 | AB (adopt* OR foster*) AND AB (parent* OR child* OR famil* OR home* OR care*) | 4,343 |
| S1 | TI (adopt* OR foster*) AND TI (parent* OR child* OR famil* OR home* OR care*) | 205 |

*ERIC*

*Searched through EBSCO-host. Search performed 11/11/2019.*

| **Search** | **Search Terms** | **Results** |
| --- | --- | --- |
| S19 | S6 AND S10 AND S14 AND S18 | 2,002 |
| S18 | S15 OR S16 OR S17 | 509,389 |
| S17 | DE "Intervention" OR DE "Early Intervention" OR DE "Therapy" | 56,135 |
| S16 | AB (treatmen* OR intervent* OR therap* OR program*) | 484,518 |
| S15 | TI (treatmen* OR intervent* OR therap* OR program*) | 127,592 |
| S14 | S11 OR S12 OR S13 | 636,277 |
| S13 | DE "Randomized Controlled Trials" OR DE "Control Groups" OR DE "Evidence" OR DE "Evidence Based Practice" OR DE "Experimental Groups" OR DE "Experimental Programs" OR DE "Effect Size" OR DE "Measurement" | 37,361 |
| S12 | AB (effect* OR trial* OR experiment* OR control* OR random* OR impact* OR compar*) | 589,697 |
| S11 | TI (effect* OR trial* OR experiment* OR control* OR random* OR impact* OR compar*) | 148,595 |
| S10 | S7 OR S8 OR S9 | 328,600 |
| S9 | DE "Attachment Behavior" OR DE "Affective Behavior" OR DE "Behavior Development" OR DE "Child Behavior" OR DE "Intimacy" OR DE "Separation Anxiety" OR DE "Anxiety" OR "Anxiety Disorders" OR DE "Posttraumatic Stress Disorder" OR DE "Child Care" OR DE "Sexual Abuse" OR DE "Child Abuse" OR DE "Caregiver Child Relationship" OR DE "Emotional Disturbances" OR DE "Emotional Adjustment" OR DE "Emotional Problems" OR DE "Psychological Needs" OR DE "Emotional Response" OR DE "Emotional Intelligence" OR DE "Emotional Experience" OR DE "Emotional Development" OR DE "Mental Health" OR DE "Affective Behavior" OR DE "Trauma" OR DE "Development" | 83,585 |
| S8 | AB (sensitiv* OR emoti* OR dyadic* OR attach* OR relation*) | 271,942 |
| S7 | TI (sensitiv* OR emoti* OR dyadic* OR attach* OR relation*) | 50,831 |
| S6 | S1 OR S2 OR S3 OR S4 OR S5 | 28,439 |
| S5 | DE “Foster Care” OR DE “Social Services” OR DE “Adoption” | 9,089 |
| S4 | AB out-of-home | 658 |
| S3 | TI out-of-home | 164 |
| S2 | AB (adopt* OR foster*) AND AB (parent* OR child* OR famil* OR home* OR care*) | 21,421 |
| S1 | TI (adopt* OR foster*) AND TI (parent* OR child* OR famil* OR home* OR care*) | 2,068 |

*Cinahl*

*Searched through EBSCO-host. Search performed 8/11/2019.*

| **Search** | **Search Terms** | **Results** |
| --- | --- | --- |
| S19 | S6 AND S10 AND S14 AND S18 | 2,809 |
| S18 | S15 OR S16 OR S17 | 1,479,961 |
| S17 | MH “Intervention Trials” OR MH “Early Childhood Intervention” | 12,081 |
| S16 | AB (treatmen* OR intervent* OR therap* OR program*) | 1,196,820 |
| S15 | TI (treatmen* OR intervent* OR therap* OR program*) | 570,373 |
| S14 | S11 OR S12 OR S13 | 1,996,654 |
| S13 | MH “Randomized Controlled Trials” OR MH “Clinical Trials” OR MH “Experimental Studies” OR MH “Quasi-Experimental Studies” OR MH “Effect Size” | 277,133 |
| S12 | AB (effect* OR trial* OR experiment* OR control* OR random* OR impact* OR compar*) | 1,652,449 |
| S11 | TI (effect* OR trial* OR experiment* OR control* OR random* OR impact* OR compar*) | 671,682 |
| S10 | S7 OR S8 OR S9 | 632,502 |
| S9 | MH “Attachment Behavior” OR MH “Behavioral and Mental Disorders” OR MH “Reactive Attachment Disorder” OR MH “Separation Anxiety” OR MH “Parent-Child Relations” OR MH “Child Abuse, Sexual” OR MH “Child Development” OR MH “Child Development Disorders” OR MH “Child Development Disorders, Pervasive” OR MH “Anxiety Disorders” OR MH “Anxiety” OR MH “Separation Anxiety” | 95,219 |
| S8 | AB (sensitiv* OR emoti* OR dyadic* OR attach* OR relation*) | 508,487 |
| S7 | TI (sensitiv* OR emoti* OR dyadic* OR attach* OR relation*) | 120,041 |
| S6 | S1 OR S2 OR S3 OR S4 OR S5 | 38,177 |
| S5 | MH "Foster Home Care" OR MM "Child, Adopted" OR MM "Child, Foster" OR MM “Foster Parents” | 5,736 |
| S4 | AB out-of-home | 829 |
| S3 | TI out-of-home | 433 |
| S2 | AB (adopt* OR foster*) AND AB (parent* OR child* OR famil* OR home* OR care*) | 32,949 |
| S1 | TI (adopt* OR foster*) AND TI (parent* OR child* OR famil* OR home* OR care*) | 4,411 |

*Academic Search Premier*

*Searched through EBSCO-host. Search performed 13/11/2019.*

| **Search** | **Search Terms** | **Results** |
| --- | --- | --- |
| S19 | S6 AND S10 AND S14 AND S18 | 3,970 |
| S18 | S15 OR S16 OR S17 | 4,296,848 |
| S17 | DE “TREATMENT effectiveness” | 91,685 |
| S16 | AB (treatmen* OR intervent* OR therap* OR program*) | 4,046,917 |
| S15 | TI (treatmen* OR intervent* OR therap* OR program*) | 922,112 |
| S14 | S11 OR S12 OR S13 | 10,972,734 |
| S13 | DE "Randomized Controlled Trials" OR DE "Experimental Design" OR DE "STATISTICAL sampling" OR DE "Clinical Trials" OR DE "Effect Size (Statistical)" OR DE “Measurement” OR DE “CONTROL groups” OR DE “CASE-control method” OR DE “MATCHED groups” | 336,741 |
| S12 | AB (effect* OR trial* OR experiment* OR control* OR random* OR impact* OR compar*) | 10,415,826 |
| S11 | TI (effect* OR trial* OR experiment* OR control* OR random* OR impact* OR compar*) | 2,543,180 |
| S10 | S7 OR S8 OR S9 | 3,010,270 |
| S9 | DE "ATTACHMENT behavior" OR DE "ATTACHMENT disorder in children" OR DE "ATTACHMENT behavior in infants" OR DE "ATTACHMENT disorder" OR DE "ATTACHMENT behavior in children" OR DE “ATTACHMENT theory (Psychology)” OR DE “ADOPTION & psychology” OR DE "STRESS (Psychology)" OR DE "POST-traumatic stress" OR DE "POST-traumatic stress disorder" OR DE "SECONDARY traumatic stress" OR DE "CHILD abuse" OR DE "PARENT-child relationship" OR DE "SEPARATION (Psychology) in children" OR DE "CHILD psychology" OR DE “CHILD development” | 164,765 |
| S8 | AB (sensitiv* OR emoti* OR dyadic* OR attach* OR relation*) | 2,781,506 |
| S7 | TI (sensitiv* OR emoti* OR dyadic* OR attach* OR relation*) | 430,161 |
| S6 | S1 OR S2 OR S3 OR S4 OR S5 | 84,717 |
| S5 | DE "FOSTER home care" OR DE "ADOPTION" OR DE "KINSHIP care" OR DE "FOSTER parents" OR DE "FOSTER mothers" OR DE "ADOPTIVE parents" OR DE "FOSTER children” OR DE "ADOPTEES" OR DE "ADOPTION" | 12,300 |
| S4 | AB out-of-home | 2,319 |
| S3 | TI out-of-home | 696 |
| S2 | AB (adopt* OR foster*) AND AB (parent* OR child* OR famil* OR home* OR care*) | 77,801 |
| S1 | TI (adopt* OR foster*) AND TI (parent* OR child* OR famil* OR home* OR care*) | 6,844 |

*PsycINFO*

*Searched through EBSCO-host. Search performed 14/11/2019.*

| **Search** | **Terms** | **Results** |
| --- | --- | --- |
| S19 | S6 AND S10 AND S14 AND S18 | 4,943 |
| S18 | S15 OR S16 OR S17 | 1,281,863 |
| S17 | DE "Intervention" OR DE "Treatment" | 130,835 |
| S16 | AB (treatmen* OR intervent* OR therap* OR program*) | 1,228,946 |
| S15 | TI (treatmen* OR intervent* OR therap* OR program*) | 376,984 |
| S14 | S11 OR S12 OR S13 | 2,493,553 |
| S13 | DE "Randomized Controlled Trials" OR DE "Experimental Design" OR DE "Random Sampling" OR DE " Randomized Effectiveness Evaluation" OR DE "Clinical Trials" OR DE "Effect Size (Statistical)" OR DE “Measurement” | 95,960 |
| S12 | AB (effect* OR trial* OR experiment* OR control* OR random* OR impact* OR compar*) | 2,325,560 |
| S11 | TI (effect* OR trial* OR experiment* OR control* OR random* OR impact* OR compar*) | 634,316 |
| S10 | S7 OR S8 OR S9 | 1,306,939 |
| S9 | DE "Attachment Behavior" OR DE "Attachment Disorders" OR DE "Attachment Theory" OR DE "Stress and Trauma Related Disorders" OR DE "Disinhibited Social Engagement Disorder" OR DE "Child Abuse" OR DE "Child Neglect" OR DE "Failure to Thrive" OR DE "Parent Child Relations" OR DE "Relationship Termination" OR DE "Separation Anxiety" OR DE "Separation Anxiety Disorder" OR DE "Separation Reactions OR DE "Emotional Development" OR DE "Emotional Security" OR DE "Object Relations" OR DE "Parent Child Relations" OR DE "Psychosocial Development" OR DE "Schema Therapy" | 81,640 |
| S8 | AB (sensitiv* OR emoti* OR dyadic* OR attach* OR relation*) | 1,201,966 |
| S7 | TI (sensitiv* OR emoti* OR dyadic* OR attach* OR relation*) | 291,762 |
| S6 | S1 OR S2 OR S3 OR S4 OR S5 | 55,035 |
| S5 | DE "Foster Care" OR DE "Foster Children" OR DE "Foster Parents" OR DE "Adoption (Child)" OR DE "Adopted Children" OR DE "Adoptive Parents" | 11,096 |
| S4 | AB out-of-home | 2,065 |
| S3 | TI out-of-home | 589 |
| S2 | AB (adopt* OR foster*) AND AB (parent* OR child* OR famil* OR home* OR care*) | 51,502 |
| S1 | TI (adopt* OR foster*) AND TI (parent* OR child* OR famil* OR home* OR care*) | 7,114 |

*Science Citation Index*

Searched through Web of Science: Thomson Reuters. Search performed 13/11/2019.

| **Search** | **Results** | **Search Terms** | |
| --- | --- | --- | --- |
| # 14 | 2,790 | | #13 AND #10 AND #7 AND #4  *Indexes=SCI-EXPANDED Timespan=All years* |
| # 13 | [6,656,128](http://apps.webofknowledge.com/summary.do?product=WOS&doc=1&qid=76&SID=D5k2JCtc1vWgZki4Rfi&search_mode=AdvancedSearch&update_back2search_link_param=yes) | | #12 OR #11  *Indexes=SCI-EXPANDED Timespan=All years* |
| # 12 | [5,302,646](http://apps.webofknowledge.com/summary.do?product=WOS&doc=1&qid=73&SID=D5k2JCtc1vWgZki4Rfi&search_mode=AdvancedSearch&update_back2search_link_param=yes) | | AB=(treatmen* OR intervent* OR therap* OR program*)  *Indexes=SCI-EXPANDED Timespan=All years* |
| # 11 | [2,322,613](http://apps.webofknowledge.com/summary.do?product=WOS&doc=1&qid=70&SID=D5k2JCtc1vWgZki4Rfi&search_mode=AdvancedSearch&update_back2search_link_param=yes) | | TI=(treatmen* OR intervent* OR therap* OR program*)  *Indexes=SCI-EXPANDED Timespan=All years* |
| # 10 | [19,325,728](http://apps.webofknowledge.com/summary.do?product=WOS&doc=1&qid=68&SID=D5k2JCtc1vWgZki4Rfi&search_mode=AdvancedSearch&update_back2search_link_param=yes) | | #9 OR #8  *Indexes=SCI-EXPANDED Timespan=All years* |
| # 9 | [15,980,525](http://apps.webofknowledge.com/summary.do?product=WOS&doc=1&qid=65&SID=D5k2JCtc1vWgZki4Rfi&search_mode=AdvancedSearch&update_back2search_link_param=yes) | | AB=(effect* OR trial* OR experiment* OR control* OR random* OR impact* OR compar*)  *Indexes=SCI-EXPANDED Timespan=All years* |
| # 8 | [6,679,549](http://apps.webofknowledge.com/summary.do?product=WOS&doc=1&qid=62&SID=D5k2JCtc1vWgZki4Rfi&search_mode=AdvancedSearch&update_back2search_link_param=yes) | | TI=(effect* OR trial* OR experiment* OR control* OR random* OR impact* OR compar*)  *Indexes=SCI-EXPANDED Timespan=All years* |
| # 7 | [4,238,696](http://apps.webofknowledge.com/summary.do?product=WOS&doc=1&qid=60&SID=D5k2JCtc1vWgZki4Rfi&search_mode=AdvancedSearch&update_back2search_link_param=yes) | | #6 OR #5  *Indexes=SCI-EXPANDED Timespan=All years* |
| # 6 | [3,638,587](http://apps.webofknowledge.com/summary.do?product=WOS&doc=1&qid=57&SID=D5k2JCtc1vWgZki4Rfi&search_mode=AdvancedSearch&update_back2search_link_param=yes) | | AB=(sensitiv* OR emoti* OR dyadic* OR attach* OR relation*)  *Indexes=SCI-EXPANDED Timespan=All years* |
| # 5 | [956,181](http://apps.webofknowledge.com/summary.do?product=WOS&doc=1&qid=54&SID=D5k2JCtc1vWgZki4Rfi&search_mode=AdvancedSearch&update_back2search_link_param=yes) | | TI=(sensitiv* OR emoti* OR dyadic* OR attach* OR relation*)  *Indexes=SCI-EXPANDED Timespan=All years* |
| # 4 | [56,988](http://apps.webofknowledge.com/summary.do?product=WOS&doc=1&qid=52&SID=D5k2JCtc1vWgZki4Rfi&search_mode=AdvancedSearch&update_back2search_link_param=yes) | | #3 OR #2 OR #1  *Indexes=SCI-EXPANDED Timespan=All years* |
| # 3 | [681](http://apps.webofknowledge.com/summary.do?product=WOS&doc=1&qid=49&SID=D5k2JCtc1vWgZki4Rfi&search_mode=AdvancedSearch&update_back2search_link_param=yes) | | TI=out-of-home OR AB= out-of-home  *Indexes=SCI-EXPANDED Timespan=All years* |
| # 2 | [54,758](http://apps.webofknowledge.com/summary.do?product=WOS&doc=1&qid=46&SID=D5k2JCtc1vWgZki4Rfi&search_mode=AdvancedSearch&update_back2search_link_param=yes) | | AB=(adopt* OR foster*) AND AB=(parent* OR child* OR famil* OR home* OR care*)  *Indexes=SCI-EXPANDED Timespan=All years* |
| # 1 | [2,731](http://apps.webofknowledge.com/summary.do?product=WOS&doc=1&qid=43&SID=D5k2JCtc1vWgZki4Rfi&search_mode=AdvancedSearch&update_back2search_link_param=yes) | | TI=(adopt* OR foster*) AND TI=(parent* OR child* OR famil* OR home* OR care*)  *Indexes=SCI-EXPANDED Timespan=All years* |

*Social Science Citation Index*

Searched through Web of Science: Thomson Reuters. Search performed 14/11/2019.

| **Search** | **Results** | **Search Terms** |
| --- | --- | --- |
| # 14 | 2,939 | #13 AND #10 AND #7 AND #4  *Indexes=SSCI Timespan=All years* |
| # 13 | 1,042,164 | #12 OR #11  *Indexes=SSCI Timespan=All years* |
| # 12 | 809,204 | AB=(treatmen* OR intervent* OR therap* OR program*)  *Indexes=SSCI Timespan=All years* |
| # 11 | 437,186 | TI=(treatmen* OR intervent* OR therap* OR program*)  *Indexes=SSCI Timespan=All years* |
| # 10 | 2,335,107 | #9 OR #8  *Indexes=SSCI Timespan=All years* |
| # 9 | 1,921,210 | AB=(effect* OR trial* OR experiment* OR control* OR random* OR impact* OR compar*)  *Indexes=SSCI Timespan=All years* |
| # 8 | 836,160 | TI=(effect* OR trial* OR experiment* OR control* OR random* OR impact* OR compar*)  *Indexes=SSCI Timespan=All years* |
| # 7 | 1,034,412 | #6 OR #5  *Indexes=SSCI Timespan=All years* |
| # 6 | 875,449 | AB=(sensitiv* OR emoti* OR dyadic* OR attach* OR relation*)  *Indexes=SSCI Timespan=All years* |
| # 5 | 300,259 | TI=(sensitiv* OR emoti* OR dyadic* OR attach* OR relation*)  *Indexes=SSCI Timespan=All years* |
| # 4 | 46,946 | #3 OR #2 OR #1  *Indexes=SSCI Timespan=All years* |
| # 3 | 1,768 | TI=out-of-home OR AB=out-of-home  *Indexes=SSCI Timespan=All years* |
| # 2 | 42,196 | AB=(adopt* OR foster*) AND AB=(parent* OR child* OR famil* OR home* OR care*)  *Indexes=SSCI Timespan=All years* |
| # 1 | 6,854 | TI=(adopt* OR foster*) AND TI=(parent* OR child* OR famil* OR home* OR care*)  *Indexes=SSCI Timespan=All years* |

*Sociological Abstracts*

Searched through ProQuest search interface. Search performed 14/11/2019.

| **Search** | **Terms** | **Results** |
| --- | --- | --- |
| S15 | S5 AND S8 AND S11 AND S14 | [1,202](https://search.proquest.com/recentsearches.recentsearchtabview.recentsearchesgridview.scrolledrecentsearchlist.checkdbssearchlink_0:rerunsearch/AE6AB898B3B9416CPQ/None?site=socabs&t:ac=RecentSearches) |
| S14 | S12 OR S13 | [178,909](https://search.proquest.com/recentsearches.recentsearchtabview.recentsearchesgridview.scrolledrecentsearchlist.checkdbssearchlink_0:rerunsearch/1A1B293900734967PQ/None?site=socabs&t:ac=RecentSearches) |
| S13 | MAINSUBJECT.EXACT("Treatment") OR MAINSUBJECT.EXACT("Treatment Methods") OR MAINSUBJECT.EXACT("Treatment Programs") OR MAINSUBJECT.EXACT("Intervention") | [24,788](https://search.proquest.com/recentsearches.recentsearchtabview.recentsearchesgridview.scrolledrecentsearchlist.checkdbssearchlink_0:rerunsearch/2345DBC3D7EC4F07PQ/None?site=socabs&t:ac=RecentSearches) |
| S12 | ti((treatmen* OR intervent* OR therap* OR program*) ) OR ab((treatmen* OR intervent* OR therap* OR program*) ) | [178,001](https://search.proquest.com/recentsearches.recentsearchtabview.recentsearchesgridview.scrolledrecentsearchlist.checkdbssearchlink_0:rerunsearch/AB7002CB5D8E4EBBPQ/None?site=socabs&t:ac=RecentSearches) |
| S11 | S9 OR S10 | [532,960](https://search.proquest.com/recentsearches.recentsearchtabview.recentsearchesgridview.scrolledrecentsearchlist.checkdbssearchlink_0:rerunsearch/DCF6658E9D764A3CPQ/None?site=socabs&t:ac=RecentSearches) |
| S10 | MAINSUBJECT.EXACT("Effects") OR MAINSUBJECT.EXACT("Treatment Outcomes") OR MAINSUBJECT.EXACT("Effectiveness") OR MAINSUBJECT.EXACT("Experiments") | [7,620](https://search.proquest.com/recentsearches.recentsearchtabview.recentsearchesgridview.scrolledrecentsearchlist.checkdbssearchlink_0:rerunsearch/6250A769174248D8PQ/None?site=socabs&t:ac=RecentSearches) |
| S9 | ti((effect* OR trial* OR experiment* OR control* OR random* OR impact* OR compar*) ) OR ab((effect* OR trial* OR experiment* OR control* OR random* OR impact* OR compar*) ) | [531,407](https://search.proquest.com/recentsearches.recentsearchtabview.recentsearchesgridview.scrolledrecentsearchlist.checkdbssearchlink_0:rerunsearch/B024D609547B45C0PQ/None?site=socabs&t:ac=RecentSearches) |
| S8 | S6 OR S7 | [536,823](https://search.proquest.com/recentsearches.recentsearchtabview.recentsearchesgridview.scrolledrecentsearchlist.checkdbssearchlink_0:rerunsearch/310E4080DD174A97PQ/None?site=socabs&t:ac=RecentSearches) |
| S7 | MAINSUBJECT.EXACT.EXPLODE("Attachment") OR MAINSUBJECT.EXACT.EXPLODE("Intimacy") OR MAINSUBJECT.EXACT.EXPLODE("Behavior") OR MAINSUBJECT.EXACT.EXPLODE("Disorders") OR MAINSUBJECT.EXACT.EXPLODE("Child Neglect") OR MAINSUBJECT.EXACT("Child Abuse") OR MAINSUBJECT.EXACT.EXPLODE("Child Development") OR MAINSUBJECT.EXACT.EXPLODE("Child Sexual Abuse") OR MAINSUBJECT.EXACT.EXPLODE("Anxiety") OR MAINSUBJECT.EXACT.EXPLODE("Affective Illness") OR MAINSUBJECT.EXACT("Psychological Development") | [219,063](https://search.proquest.com/recentsearches.recentsearchtabview.recentsearchesgridview.scrolledrecentsearchlist.checkdbssearchlink_0:rerunsearch/999C31FA40924093PQ/None?site=socabs&t:ac=RecentSearches) |
| S6 | ti((sensitiv* OR emoti* OR dyadic* OR attach* OR relation*) ) OR ab((sensitiv* OR emoti* OR dyadic* OR attach* OR relation*) ) | [389,484](https://search.proquest.com/recentsearches.recentsearchtabview.recentsearchesgridview.scrolledrecentsearchlist.checkdbssearchlink_0:rerunsearch/D4B02F610B254DE8PQ/None?site=socabs&t:ac=RecentSearches) |
| S5 | S1 OR S2 OR S3 OR S4 | [18,696](https://search.proquest.com/recentsearches.recentsearchtabview.recentsearchesgridview.scrolledrecentsearchlist.checkdbssearchlink_0:rerunsearch/511A943CD0A147B9PQ/None?site=socabs&t:ac=RecentSearches) |
| S4 | MAINSUBJECT.EXACT("Foster Children") OR MAINSUBJECT.EXACT("Foster Care") OR MAINSUBJECT.EXACT("Surrogate Parents") OR MAINSUBJECT.EXACT("Adoption") OR MAINSUBJECT.EXACT("Transracial Adoption") OR MAINSUBJECT.EXACT("Adopted Children") OR MAINSUBJECT.EXACT("International Adoption") OR MAINSUBJECT.EXACT("Placement") OR MAINSUBJECT.EXACT("Permanency Planning") OR MAINSUBJECT.EXACT("Adoptive Parents") | [4,603](https://search.proquest.com/recentsearches.recentsearchtabview.recentsearchesgridview.scrolledrecentsearchlist.checkdbssearchlink_0:rerunsearch/D6644FAF50D84E89PQ/None?site=socabs&t:ac=RecentSearches) |
| S3 | ti("out-of-home" OR "out of home") OR ab("out-of-home" OR "out of home") | [444](https://search.proquest.com/recentsearches.recentsearchtabview.recentsearchesgridview.scrolledrecentsearchlist.checkdbssearchlink_0:rerunsearch/9969114BF0F141B9PQ/None?site=socabs&t:ac=RecentSearches) |
| S2 | ab((adopt* OR foster*) ) AND ab((parent* OR child* OR famil* OR home* OR care*) ) | [16,380](https://search.proquest.com/recentsearches.recentsearchtabview.recentsearchesgridview.scrolledrecentsearchlist.checkdbssearchlink_0:rerunsearch/D7848C2B6DAE435FPQ/None?site=socabs&t:ac=RecentSearches) |
| S1 | ti((adopt* OR foster*) ) AND ti((parent* OR child* OR famil* OR home* OR care*) ) | [1,775](https://search.proquest.com/recentsearches.recentsearchtabview.recentsearchesgridview.scrolledrecentsearchlist.checkdbssearchlink_0:rerunsearch/EBCAC5651BED46BEPQ/None?site=socabs&t:ac=RecentSearches) |

**Searches on other web resources**

*ProQuest Dissertations and Theses*

Searched through ProQuest search interface. Search performed 18/11/2019.

| **Search** | **Terms** | **Results** |
| --- | --- | --- |
| S15 | S5 AND S8 AND S11 AND S14 | [1,214](https://search.proquest.com/recentsearches.recentsearchtabview.recentsearchesgridview.scrolledrecentsearchlist.checkdbssearchlink_0:rerunsearch/F575240218AE481APQ/None?site=pqdtglobal&t:ac=RecentSearches) |
| S14 | [S12 OR S13](https://search.proquest.com/recentsearches.recentsearchtabview.recentsearchesgridview.scrolledrecentsearchlist.checkdbssearchlink:rerunsearch/49E612E2B62D470APQ/None?site=pqdtglobal&t:ac=RecentSearches) | [814,854](https://search.proquest.com/recentsearches.recentsearchtabview.recentsearchesgridview.scrolledrecentsearchlist.checkdbssearchlink_0:rerunsearch/49E612E2B62D470APQ/None?site=pqdtglobal&t:ac=RecentSearches) |
| S13 | [ti((treatmen* OR intervent* OR therap* OR program*)) OR ab((treatmen* OR intervent* OR therap* OR program*))](https://search.proquest.com/recentsearches.recentsearchtabview.recentsearchesgridview.scrolledrecentsearchlist.checkdbssearchlink:rerunsearch/41DC9C1C8966459FPQ/None?site=pqdtglobal&t:ac=RecentSearches) | [814,854](https://search.proquest.com/recentsearches.recentsearchtabview.recentsearchesgridview.scrolledrecentsearchlist.checkdbssearchlink_0:rerunsearch/41DC9C1C8966459FPQ/None?site=pqdtglobal&t:ac=RecentSearches) |
| S12 | [ti((treatmen* OR intervent* OR therap* OR program*)) OR ab((treatmen* OR intervent* OR therap* OR program*))](https://search.proquest.com/recentsearches.recentsearchtabview.recentsearchesgridview.scrolledrecentsearchlist.checkdbssearchlink:rerunsearch/229E8A92879E4181PQ/None?site=pqdtglobal&t:ac=RecentSearches) | [814,854](https://search.proquest.com/recentsearches.recentsearchtabview.recentsearchesgridview.scrolledrecentsearchlist.checkdbssearchlink_0:rerunsearch/229E8A92879E4181PQ/None?site=pqdtglobal&t:ac=RecentSearches) |
| S11 | [S9 OR S10](https://search.proquest.com/recentsearches.recentsearchtabview.recentsearchesgridview.scrolledrecentsearchlist.checkdbssearchlink:rerunsearch/4593AE2121D5459DPQ/None?site=pqdtglobal&t:ac=RecentSearches) | [2,433,732](https://search.proquest.com/recentsearches.recentsearchtabview.recentsearchesgridview.scrolledrecentsearchlist.checkdbssearchlink_0:rerunsearch/4593AE2121D5459DPQ/None?site=pqdtglobal&t:ac=RecentSearches) |
| S10 | [mainsubject("effectiveness") OR mainsubject("effectiveness studies") OR mainsubject("effects")](https://search.proquest.com/recentsearches.recentsearchtabview.recentsearchesgridview.scrolledrecentsearchlist.checkdbssearchlink:rerunsearch/28ACFBEF9B00427DPQ/None?site=pqdtglobal&t:ac=RecentSearches) | [2,414](https://search.proquest.com/recentsearches.recentsearchtabview.recentsearchesgridview.scrolledrecentsearchlist.checkdbssearchlink_0:rerunsearch/28ACFBEF9B00427DPQ/None?site=pqdtglobal&t:ac=RecentSearches) |
| S9 | [ti((effect* OR trial* OR experiment* OR control* OR random* OR impact* OR compar*)) OR ab((effect* OR trial* OR experiment* OR control* OR random* OR impact* OR compar*))](https://search.proquest.com/recentsearches.recentsearchtabview.recentsearchesgridview.scrolledrecentsearchlist.checkdbssearchlink:rerunsearch/74B01BE50DBE4F99PQ/None?site=pqdtglobal&t:ac=RecentSearches) | [2,433,541](https://search.proquest.com/recentsearches.recentsearchtabview.recentsearchesgridview.scrolledrecentsearchlist.checkdbssearchlink_0:rerunsearch/74B01BE50DBE4F99PQ/None?site=pqdtglobal&t:ac=RecentSearches) |
| S8 | [S6 OR S7](https://search.proquest.com/recentsearches.recentsearchtabview.recentsearchesgridview.scrolledrecentsearchlist.checkdbssearchlink:rerunsearch/9BC53B1C3EC34E7EPQ/None?site=pqdtglobal&t:ac=RecentSearches) | [1,085,189](https://search.proquest.com/recentsearches.recentsearchtabview.recentsearchesgridview.scrolledrecentsearchlist.checkdbssearchlink_0:rerunsearch/9BC53B1C3EC34E7EPQ/None?site=pqdtglobal&t:ac=RecentSearches) |
| S7 | [mainsubject("behavior") OR mainsubject("behavior disorders") OR mainsubject("behavioral psychology") OR mainsubject("child & adolescent psychiatry") OR mainsubject("child abuse & neglect") OR mainsubject("child development") OR mainsubject("child placement") OR mainsubject("child psychology") OR mainsubject("child support") OR mainsubject("child trauma") OR mainsubject("child welfare") OR mainsubject("emotional abuse") OR mainsubject("emotional disorders") OR mainsubject("emotions") OR mainsubject("dyadic interaction") OR mainsubject("sensitivity")](https://search.proquest.com/recentsearches.recentsearchtabview.recentsearchesgridview.scrolledrecentsearchlist.checkdbssearchlink:rerunsearch/E212671B63364085PQ/None?site=pqdtglobal&t:ac=RecentSearches) | [35,085](https://search.proquest.com/recentsearches.recentsearchtabview.recentsearchesgridview.scrolledrecentsearchlist.checkdbssearchlink_0:rerunsearch/E212671B63364085PQ/None?site=pqdtglobal&t:ac=RecentSearches) |
| S6 | [ti((sensitiv* OR emoti* OR dyadic* OR attach* OR relation*)) OR ab((sensitiv* OR emoti* OR dyadic* OR attach* OR relation*))](https://search.proquest.com/recentsearches.recentsearchtabview.recentsearchesgridview.scrolledrecentsearchlist.checkdbssearchlink:rerunsearch/ADFF8E8573F94DBFPQ/None?site=pqdtglobal&t:ac=RecentSearches) | [1,067,503](https://search.proquest.com/recentsearches.recentsearchtabview.recentsearchesgridview.scrolledrecentsearchlist.checkdbssearchlink_0:rerunsearch/ADFF8E8573F94DBFPQ/None?site=pqdtglobal&t:ac=RecentSearches) |
| S5 | [S1 OR S2 OR S3 OR S4](https://search.proquest.com/recentsearches.recentsearchtabview.recentsearchesgridview.scrolledrecentsearchlist.checkdbssearchlink:rerunsearch/6BB99185D91349D3PQ/None?site=pqdtglobal&t:ac=RecentSearches) | [10,142](https://search.proquest.com/recentsearches.recentsearchtabview.recentsearchesgridview.scrolledrecentsearchlist.checkdbssearchlink_0:rerunsearch/6BB99185D91349D3PQ/None?site=pqdtglobal&t:ac=RecentSearches) |
| S4 | [mainsubject("foster care") OR mainsubject("Kinship foster care") OR mainsubject("Adoption”)](https://search.proquest.com/recentsearches.recentsearchtabview.recentsearchesgridview.scrolledrecentsearchlist.checkdbssearchlink:rerunsearch/F019F380237F4190PQ/None?site=pqdtglobal&t:ac=RecentSearches) | [299](https://search.proquest.com/recentsearches.recentsearchtabview.recentsearchesgridview.scrolledrecentsearchlist.checkdbssearchlink_0:rerunsearch/F019F380237F4190PQ/None?site=pqdtglobal&t:ac=RecentSearches) |
| S3 | [ti("out-of-home" OR "out of home") OR ab("out-of-home" OR "out of home")](https://search.proquest.com/recentsearches.recentsearchtabview.recentsearchesgridview.scrolledrecentsearchlist.checkdbssearchlink:rerunsearch/DF7F65C753EC4586PQ/None?site=pqdtglobal&t:ac=RecentSearches) | [647](https://search.proquest.com/recentsearches.recentsearchtabview.recentsearchesgridview.scrolledrecentsearchlist.checkdbssearchlink_0:rerunsearch/DF7F65C753EC4586PQ/None?site=pqdtglobal&t:ac=RecentSearches) |
| S2 | [ab((adopt* OR foster*)) NEAR/6 ab((parent* OR child* OR famil* OR home* OR care*))](https://search.proquest.com/recentsearches.recentsearchtabview.recentsearchesgridview.scrolledrecentsearchlist.checkdbssearchlink:rerunsearch/7BDB9D2E595B46B5PQ/None?site=pqdtglobal&t:ac=RecentSearches) | [8,754](https://search.proquest.com/recentsearches.recentsearchtabview.recentsearchesgridview.scrolledrecentsearchlist.checkdbssearchlink_0:rerunsearch/7BDB9D2E595B46B5PQ/None?site=pqdtglobal&t:ac=RecentSearches) |
| S1 | [ti((adopt* OR foster*) ) AND ti((parent* OR child* OR famil* OR home* OR care*))](https://search.proquest.com/recentsearches.recentsearchtabview.recentsearchesgridview.scrolledrecentsearchlist.checkdbssearchlink:rerunsearch/F58441D0A2294DE8PQ/None?site=pqdtglobal&t:ac=RecentSearches) | [2,934](https://search.proquest.com/recentsearches.recentsearchtabview.recentsearchesgridview.scrolledrecentsearchlist.checkdbssearchlink_0:rerunsearch/F58441D0A2294DE8PQ/None?site=pqdtglobal&t:ac=RecentSearches) |

*EBSCO Open Dissertations*

*Searched through EBSCO-host. Search performed 18/11/2019.*

| **Search** | **Terms** | **Results** |
| --- | --- | --- |
| S17 | S4 AND S8 AND S12 AND S16 | 309 |
| S16 | S13 OR S14 OR S15 | 180,652 |
| S15 | SU (treatmen* OR intervent* OR therap* OR program*) | 20,485 |
| S14 | AB (treatmen* OR intervent* OR therap* OR program*) | 154,791 |
| S13 | TI (treatmen* OR intervent* OR therap* OR program*) | 41,495 |
| S12 | S9 OR S10 OR S11 | 521,410 |
| S11 | SU (effect* OR trial* OR experiment* OR control* OR random* OR impact* OR compar*) | 25,693 |
| S10 | AB (effect* OR trial* OR experiment* OR control* OR random* OR impact* OR compar*) | 448,141 |
| S9 | TI (effect* OR trial* OR experiment* OR control* OR random* OR impact* OR compar*) | 146,108 |
| S8 | S5 OR S6 OR S7 | 224,448 |
| S7 | SU (sensitiv* OR emoti* OR dyadic* OR attach* OR relation*) | 13,848 |
| S6 | AB (sensitiv* OR emoti* OR dyadic* OR attach* OR relation*) | 197,350 |
| S5 | TI (sensitiv* OR emoti* OR dyadic* OR attach* OR relation*) | 42,265 |
| S4 | S1 OR S2 OR S3 | 2,762 |
| S3 | SU (adopt* OR foster* OR “out-of-home”) | 882 |
| S2 | AB (adopt* OR foster*) N6 (parent* OR child* OR famil* OR home* OR care*) | 1,998 |
| S1 | TI (adopt* OR foster*) AND TI (parent* OR child* OR famil* OR home* OR care*) | 582 |

*OpenGrey*

<http://www.opengrey.eu/>

Searches were performed in the standard search field. The searches were performed in October 2020.

We used a combination of search terms for the population (such as foster care, variations of adoptive) and terms for either the study type/method (such as intervention, effect, trial), the type of publication (such as report, working paper, dissertation).

328 results were screened using the inclusion criteria stated in the protocol.

*Google Scholar*

<https://scholar.google.com/>

Searches were performed in the standard search field. Searches were performed as title searches, using the limiter “allintitle:”.  The searches were performed in October 2020.

We used a combination of search terms for the population (such as foster care, variations of adoptive) and terms for either the study type/method (such as intervention, effect, trial), the type of publication (such as report, working paper, dissertation) or the term “attachment”.

648 results were screened using the inclusion criteria stated in the protocol.

We also searched Google Scholar for Danish, Swedish and Norwegian reports separately. We searched with terms in Danish (familiepleje, plejefamilie, adoptivbørn and tilknytning), Swedish (fostervård, fosterhem and anslutning) and Norwegian (fosterhjem, adopsjon and intervensjon).

1128 results were screened using the inclusion criteria stated in the protocol.

*Google*

<https://www.google.dk/>

Searches were performed in the standard search field. Searches were performed as title searches, using the limiter “allintitle:”.  The searches were performed in October 2020.

We used a combination of search terms for the population (such as foster care, variations of adoptive) and terms for either the study type/method (such as intervention, effect, trial), the type of publication (such as report, working paper, dissertation) or the term “attachment”.

201 results were screened using the inclusion criteria stated in the protocol.

*Social Care Online*

<https://www.scie-socialcareonline.org.uk/>

Searches were performed in the standard search field. The searches were performed in October 2020.

We used a combination of search terms for the population (such as foster care, variations of adoptive) and terms for either the study type/method (such as intervention, effect, trial), the type of publication (such as report, working paper, dissertation) or the term “attachment”.

476 results were screened using the inclusion criteria stated in the protocol.

*Social Science Research Network*

[www.ssm.com/index.cfm/en/](http://www.ssm.com/index.cfm/en/)

Searches were performed in the Advanced Search search field. Searches were limited to “Title, Abstract & Keywords”.  The searches were performed in October 2020.

We used a combination of search terms for the population (such as foster care, variations of adoptive) and terms for either the study type/method (such as intervention, effect, trial), the type of publication (such as report, working paper, dissertation) or the term “attachment”.

242 results were screened using the inclusion criteria stated in the protocol.

*Danish National Research Database*

<https://www.forskningsdatabasen.dk/>

The database is no longer in service as of 01/01/2021.

Searches were performed in the main search field. The searches were performed in October 2020.

We used a combination of search terms for the population (such as foster care, variations of adoptive) and terms for either the study type/method (such as intervention, effect, trial), the type of publication (such as report, working paper, dissertation) or the term “attachment”.

165 results were screened using the inclusion criteria stated in the protocol.

*DIVA*

<https://www.diva-portal.org/smash/search.jsf?dswid=4201&searchType=RESEARCH&faces-redirect=true&query=&af=%5B%5D&aq=%5B%5B%5D%5D&aqe=%5B%5D&aq2=%5B%5B%5D%5D>

Searches were performed in the “Advanced Search – Research Publications” section, in the free-text search field. The searches were performed in January 2021.

We used a combination of search terms for the population (such as foster care, variations of adoptive) and terms for either the study type/method (such as intervention, effect, trial), the type of publication (such as report, working paper, dissertation) or the term “attachment”.

314 results were screened using the inclusion criteria stated in the protocol.

*NORA*

<https://nora.openaccess.no/?siteLanguage=nor>

Searches were performed in the main search field. The searches were performed in January 2021.

We used a combination of search terms for the population (such as foster care, variations of adoptive) and terms for either the study type/method (such as intervention, effect, trial), the type of publication (such as report, working paper, dissertation) or the term “attachment”.

We also searched with Norwegian search terms for the different aspects, such as: fosterhjem, adopsjon, tilknytning and intervensjon.

227 results were screened using the inclusion criteria stated in the protocol.

*SocArXiv*

<https://osf.io/preprints/socarxiv/>

Searches were performed in the standard search field. The searches were performed in October 2020.

We used a combination of search terms for the population (such as foster care, variations of adoptive) and terms for either the study type/method (such as intervention, effect, trial), the type of publication (such as report, working paper, dissertation).

78 results were screened using the inclusion criteria stated in the protocol.

*Citation tracking*

We checked the references of the following previous systematic reviews: [Schoemaker 2019](#REF-Schoemaker-2019), [Kerr 2014](#REF-Kerr-2014), [Dozier 2008](#REF-Dozier-2008), [Barlow 2015](#REF-Barlow-2015), [Dozier 2001](#REF-Dozier-2001) and of all of the included studies.
